# Supplementary material for: hdWGCNA identifies co-expression networks in high-dimensional transcriptomics data
Source: Cell Rep Methods. 2023 Jun 12;3(6):100498. doi: 10.1016/j.crmeth.2023.100498 (PMC10326379; doi:10.1016/j.crmeth.2023.100498)
Supplement: Document S1. Figures S1–S11 [file mmc1.pdf]

Cell Reports Methods, Volume *3*

Supplemental information

hdWGCNA identifies co-expression networks in high-dimensional transcriptomics data

Samuel Morabito, Fairlie Reese, Negin Rahimzadeh, Emily Miyoshi, and Vivek Swarup

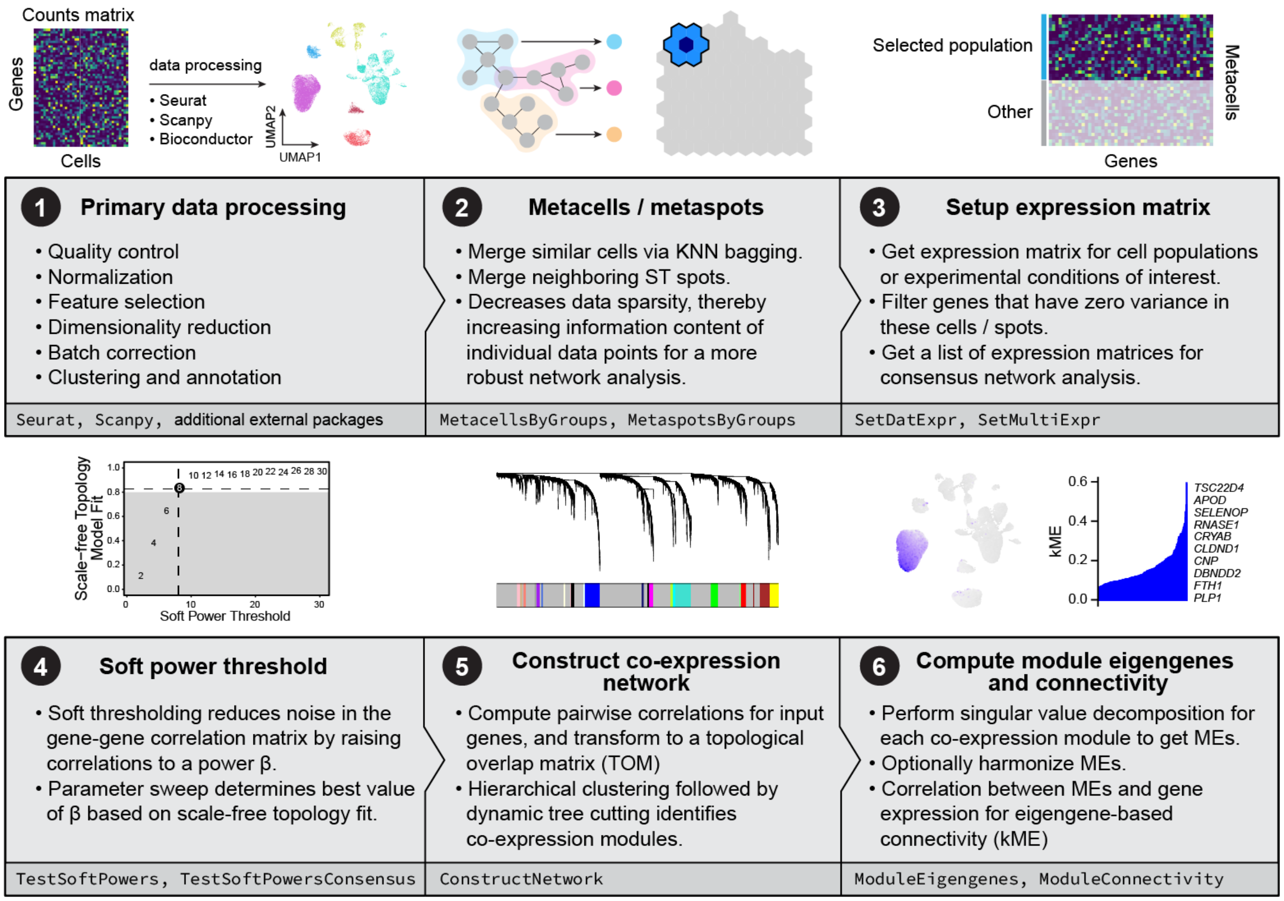

Figure S1. Schematic of the hdWGCNA workflow, related to Figure 1. 1. Prior to analysis with the hdWGCNA R package, the input single-cell or spatial dataset must be fully processed. This includes quality control, data normalization, feature selection, dimensionality reduction, batch correction (if needed), and clustering. These steps can be done using popular packages such as Seurat[1–3] or SCANPY[4]. Regardless of the pipeline used, the dataset must be formatted as a Seurat object prior to running hdWGCNA. **2.** The functions `MetacellsByGroups` and `MetaspotsByGroups` are used to aggregate transcriptomically similar cells into metacells and spatially proximal spots into metaspots respectively. **3.** hdWGCNA requires the user to explicitly specify the expression matrix that will be used for network analysis using the function `SetDatExpr`. For consensus network analysis, a list of expression matrices for each dataset or condition is specified with `SetMultiExpr`. **4.** Different values for the soft-power threshold β are tested using the functions `TestSoftPowers` and `TestSoftPowersConsensus`. The gene-gene correlation adjacency matrix is computed and raised to a power β as a soft threshold, and the degree distribution of this augmented is fit to a power law distribution to assess the scale-free topology. **5.** Co-expression network computation and gene module detection is performed in one step using the function `ConstructNetwork`. **6.** Module eigengenes (MEs) are computed using the `ModuleEigengene` function, optionally allowing for regression and harmonization of covariates such as sequencing batch. Eigengene-based connectivity (kME) is computed for each gene using the `ModuleConnectivity` function.

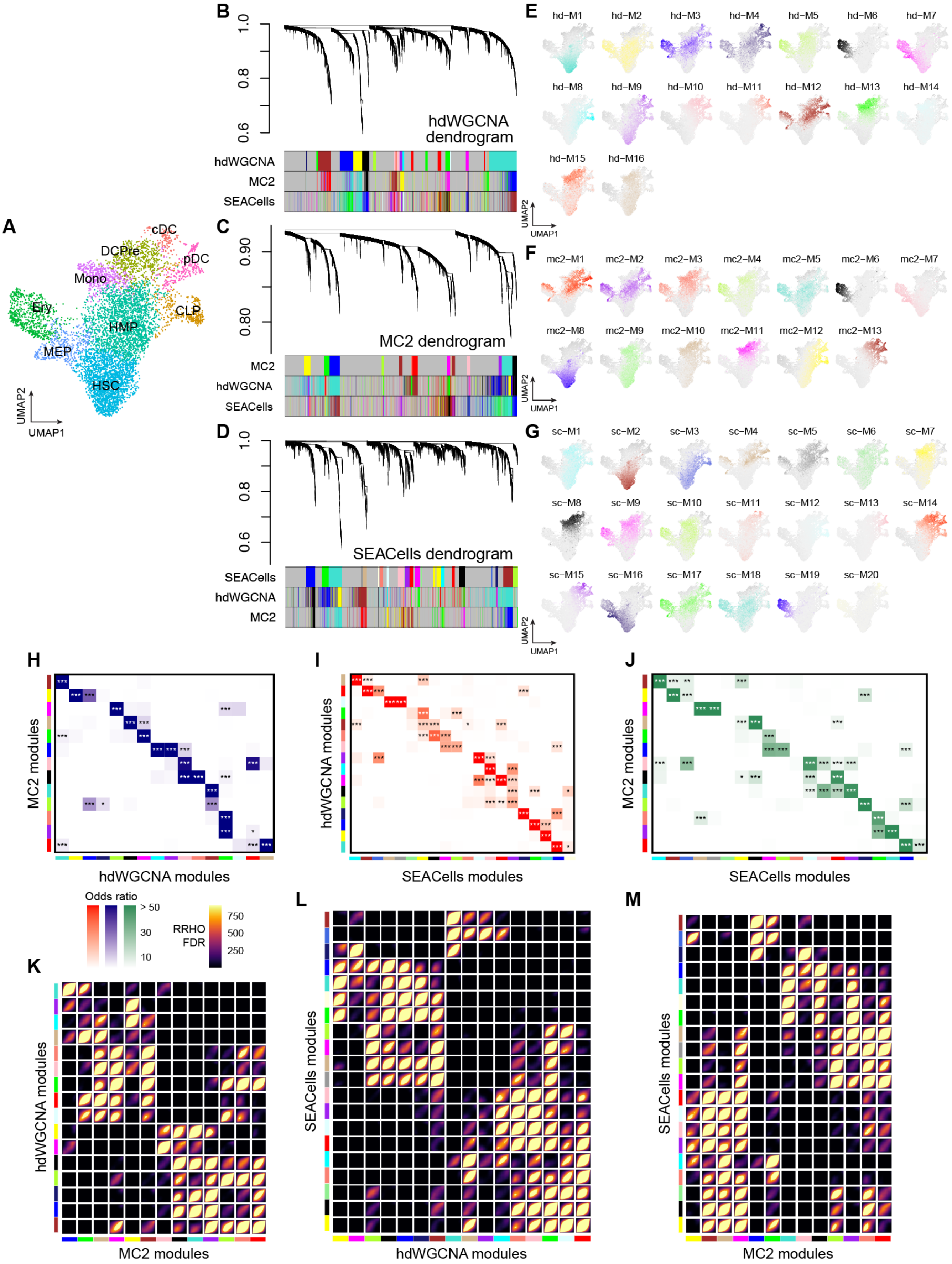

Figure S2. Comparison of metacell algorithms for co-expression network analysis. Caption on the next page ⟶

Figure S2. Comparison of metacell algorithms for co-expression network analysis, related to Figure 1. A. UMAP plot of the 6,800 CD34+ hematopoietic stem and progenitor stem cells scRNA-seq dataset[5] colored by annotations from the original study. **B-D.** hdWGCNA dendrograms for the co-expression networks constructed with the hdWGCNA (**B**), MC2 (**C**), and SEACells (**D**) algorithms. Module assignments are shown below the dendrograms **E-G.** UMAP plots as in (**A**) colored by MEs for the co-expression modules derived from the different metacell approaches. **H-J.** Module overlap comparisons between the different methods. Test was performed using Fisher's exact test, and we report the odds ratio and FDR corrected p-values. **K-M.** Rank-rank hypergeometric overlap (RRHO)[6] heatmaps comparing the ranks of kMEs for pairs of modules derived from the expression matrices from the different metacell algorithms.

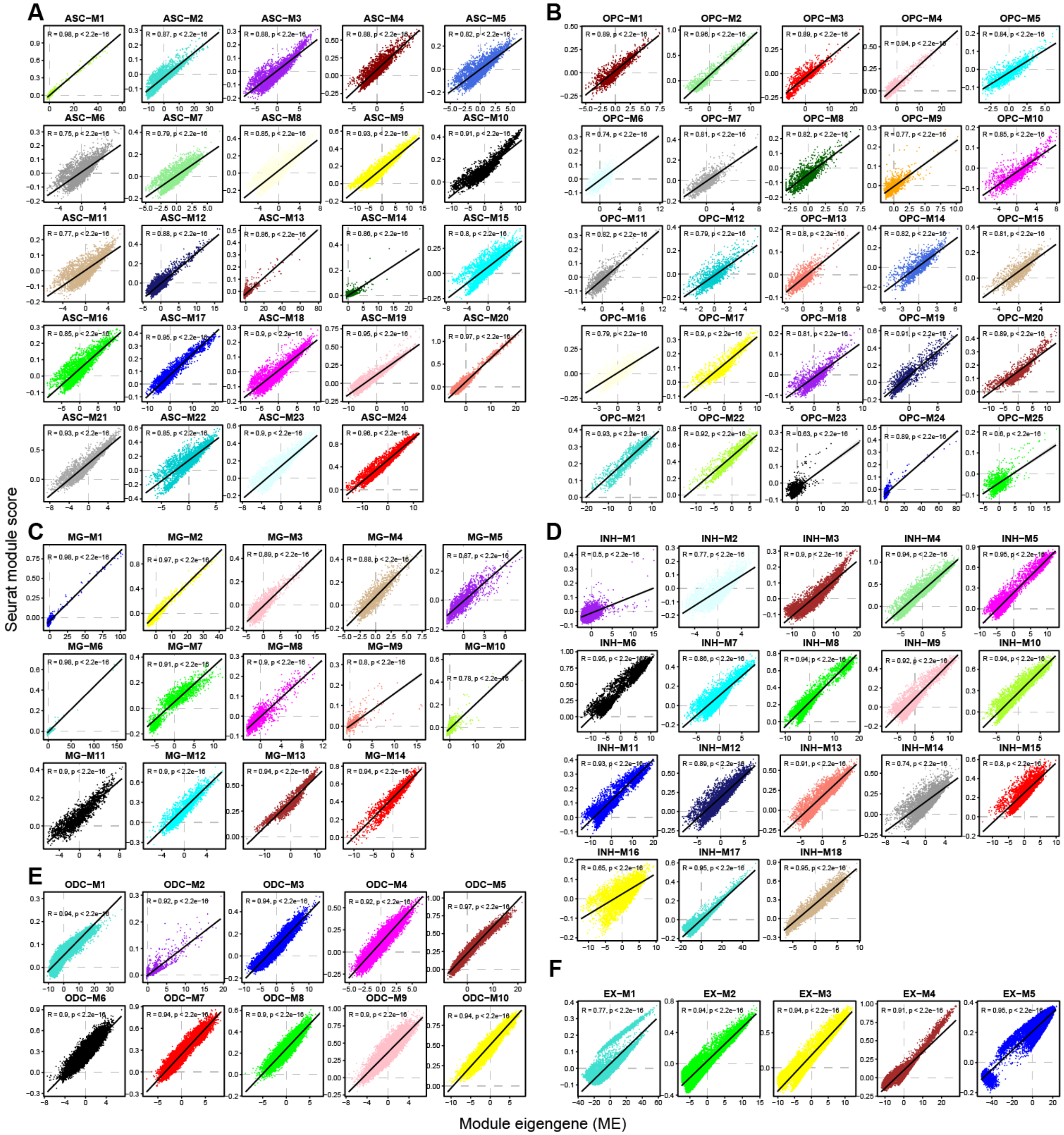

Figure S3. Correlation of module eigengenes and Seurat module scores, related to Figure 1. For each module in the human prefrontal cortex (PFC) snRNA-seq dataset[7], we computed Seurat module scores using the *AddModuleScore* function, and correlated with module eigengenes (MEs). We visualized the results as scatter plots with linear regression lines (95% confidence interval shown in grey) for ASC (**A**), OPC (**B**), MG (**C**), INH (**D**), ODC (**E**), and EX (**F**).

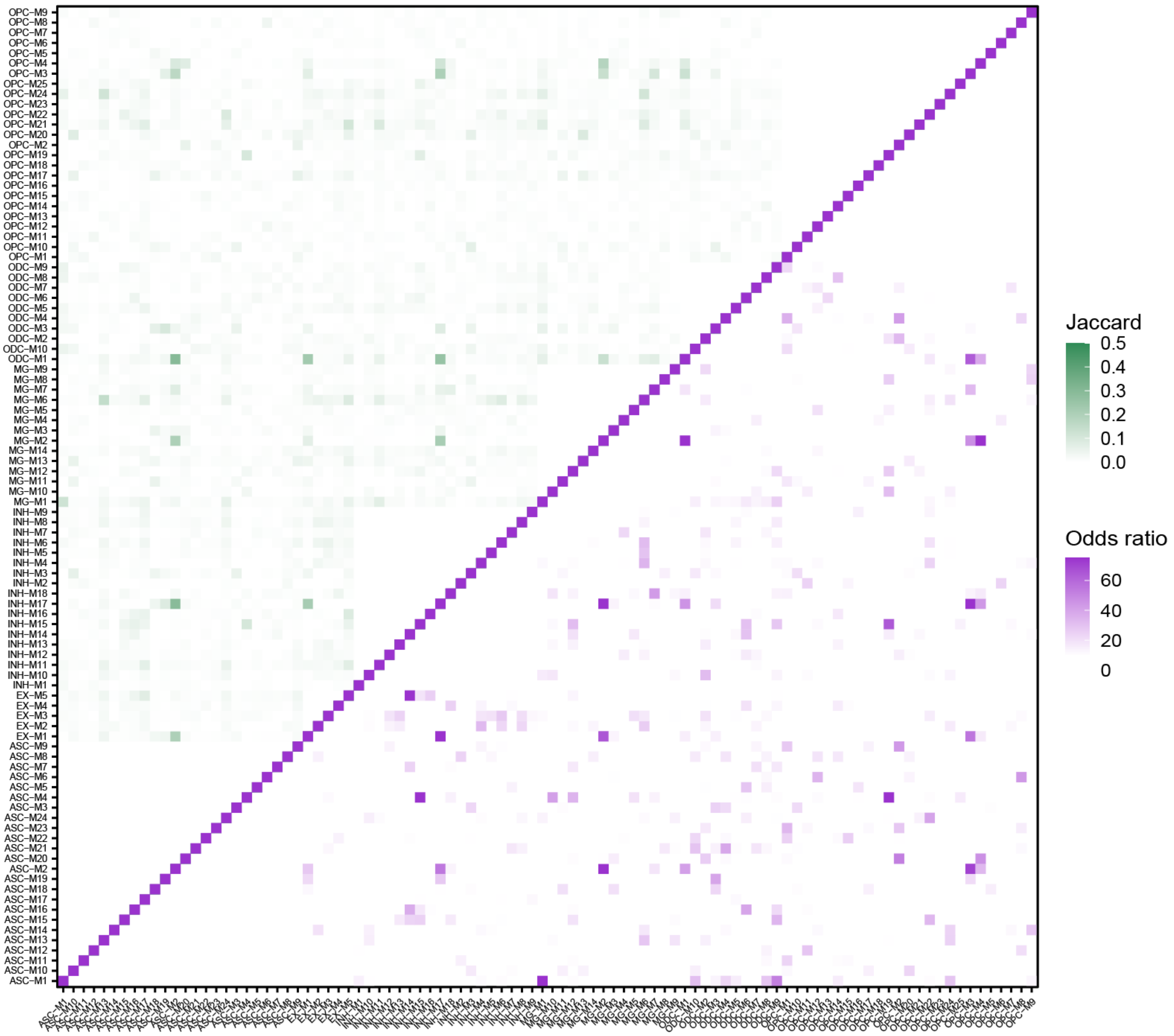

Figure S4. Module overlap analysis of cell-type-specific human PFC co-expression modules, related to Figure 1. We used Fisher's exact test to perform pairwise comparisons of gene sets from the 96 cell-type-specific co-expression modules from the Zhou *et al.* 2019 human prefrontal cortex (PFC) dataset[7]. We report the results of these module overlap tests using the Jaccard index in the upper triangle of the heatmap, and the odds ratio in the lower triangle. The diagonal of the heatmap represents the module overlapping with itself.

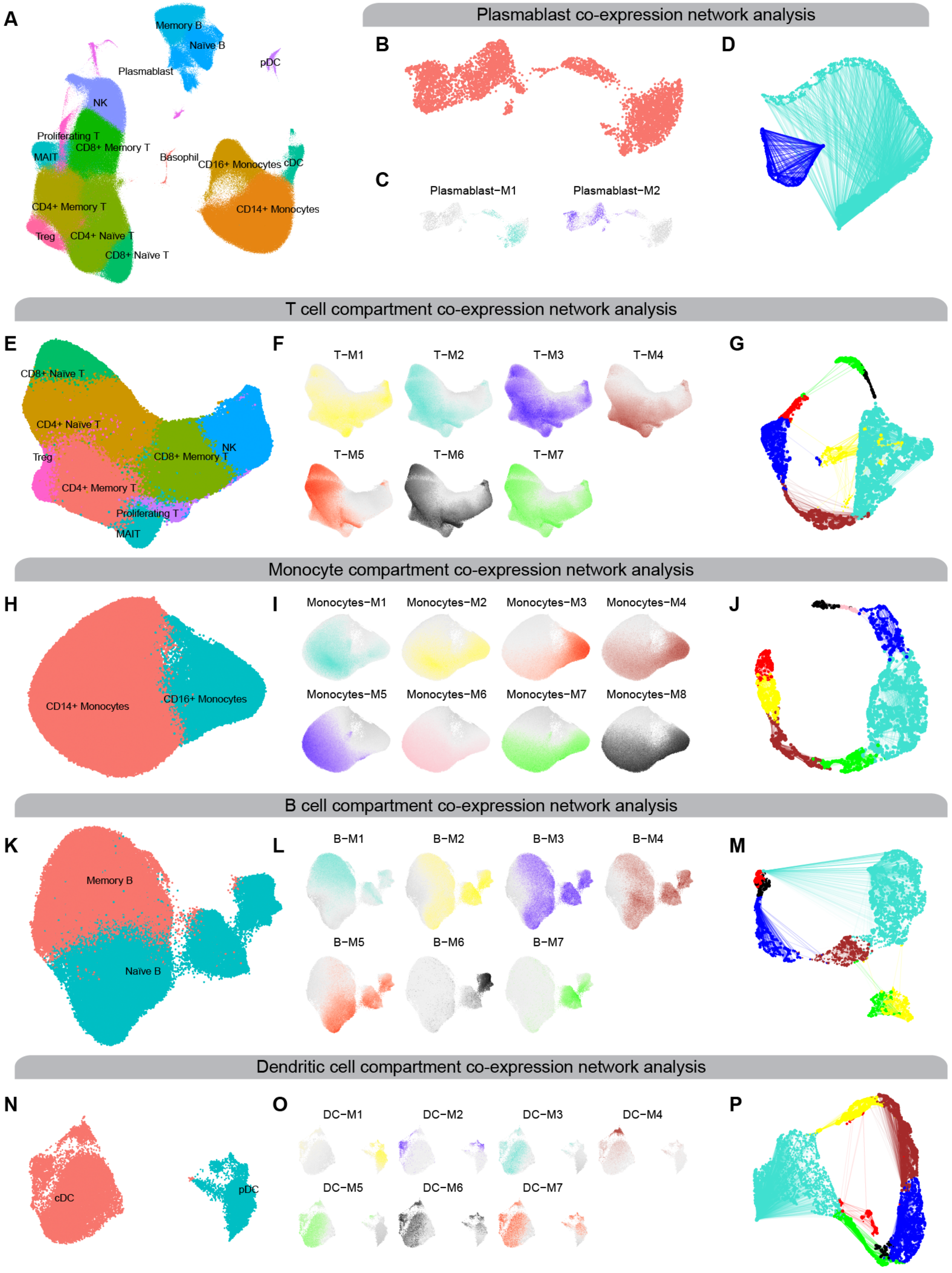

Figure S5. Iterative co-expression network analysis of major cell compartments in the Parse Biosciences 1M PBMC dataset, related to Figure 1. A. UMAP plot of 965,363 PBMCs from 12 healthy donors and 12 Type-1 diabetic donors profiled with the Parse Biosciences Evercode Whole Transcriptome Mega protocol. Cells are colored by cell-type annotations. **B,E,H,K,N.** Individual UMAP plots computed for each cell compartment separately for each cell compartment. **C,F,I,L,O.** UMAP plots for each cell compartment colored by module eigengenes (MEs). **D,G,J,M,P** UMAP plots of the co-expression networks for each cell compartment, colored by gene module assignment. Nodes represent genes and edges represent co-expression links. Network edges were downsampled for visual clarity.

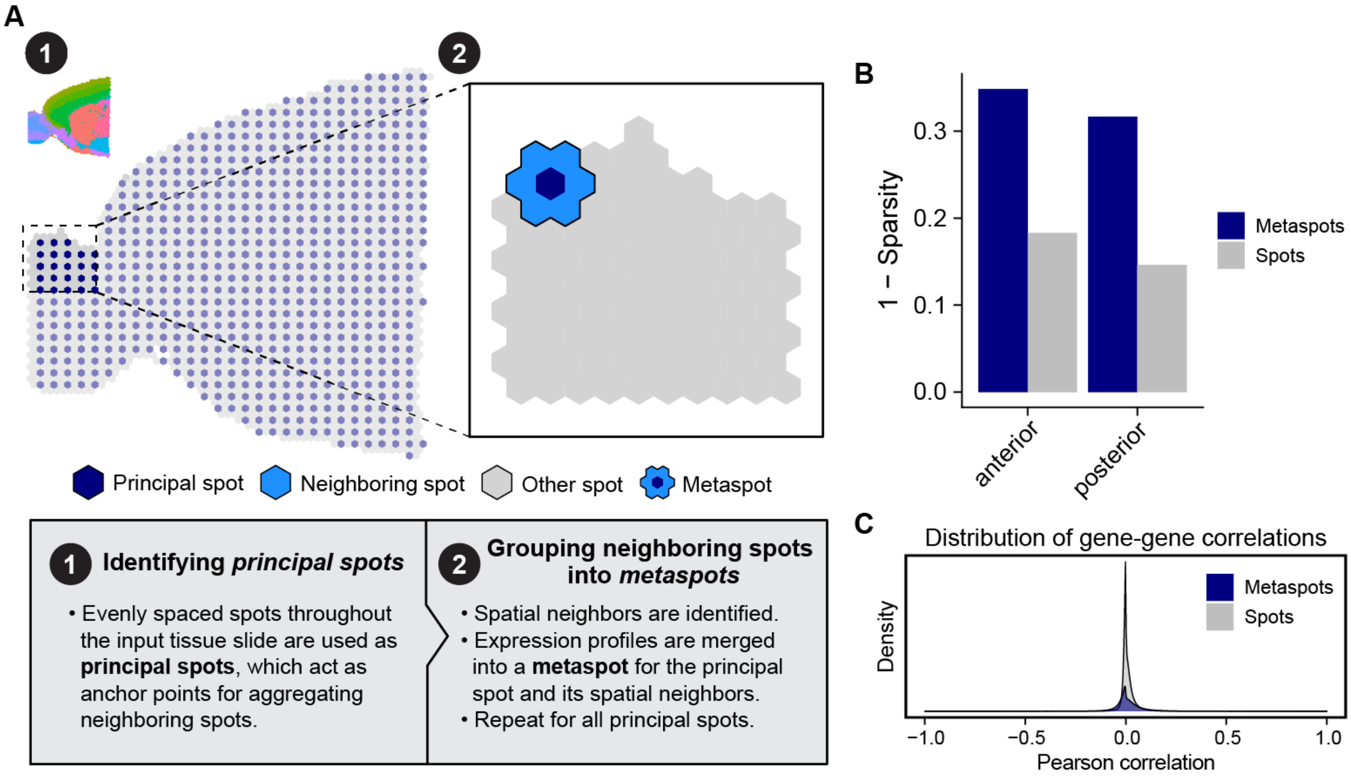

Figure S6. Metaspot aggregation for co-expression network analysis in spatial transcriptomics, related to Figure 3. A. Schematic representation of the metaspot construction process. A grid of evenly spaced *principal spots* are specified throughout the given input ST section. The expression values for each principal spot and its direct neighbors are merged into a single metaspot expression profile. This procedure yields a metaspot expression matrix for the given input ST section. **B.** Expression matrix density (1 - sparsity) for the ST and metaspot expression matrices in the anterior and posterior mouse brain samples. **C.** Density plot showing the distribution of pairwise Pearson correlations between genes from the ST expression matrix and the metaspot expression matrix.

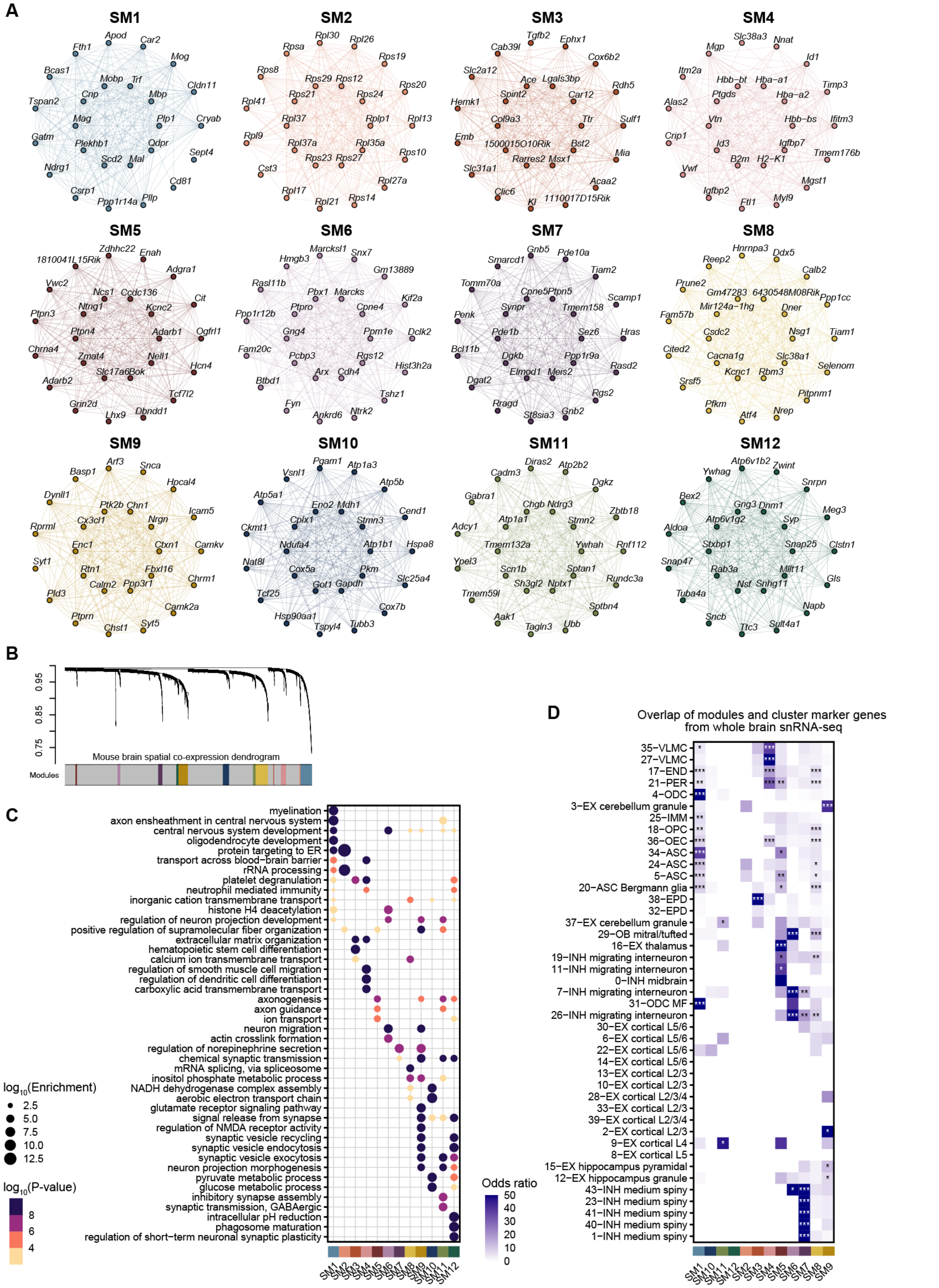

Figure S7. Mouse brain spatial transcriptomics co-expression network analysis, related to Figure 3. Caption on the next page ⟶ **A.** Hub gene networks for each spatial co-expression module. The top 25 hub genes ranked by kME are visualized. Nodes represent genes, and edges represent co-expression links. **B.** Dendrogram showing the hierarchical clustering of genes into co-expression modules based on the topological overlap matrix (TOM). **C.** Dot plot showing selected GO term enrichment results for each co-expression module. **D.** Heatmap showing gene overlap tests (Fisher's exact test) comparing the spatial co-expression modules to differentially expressed genes (DEGs) in each cluster from a whole mouse brain snRNA-seq dataset [8].

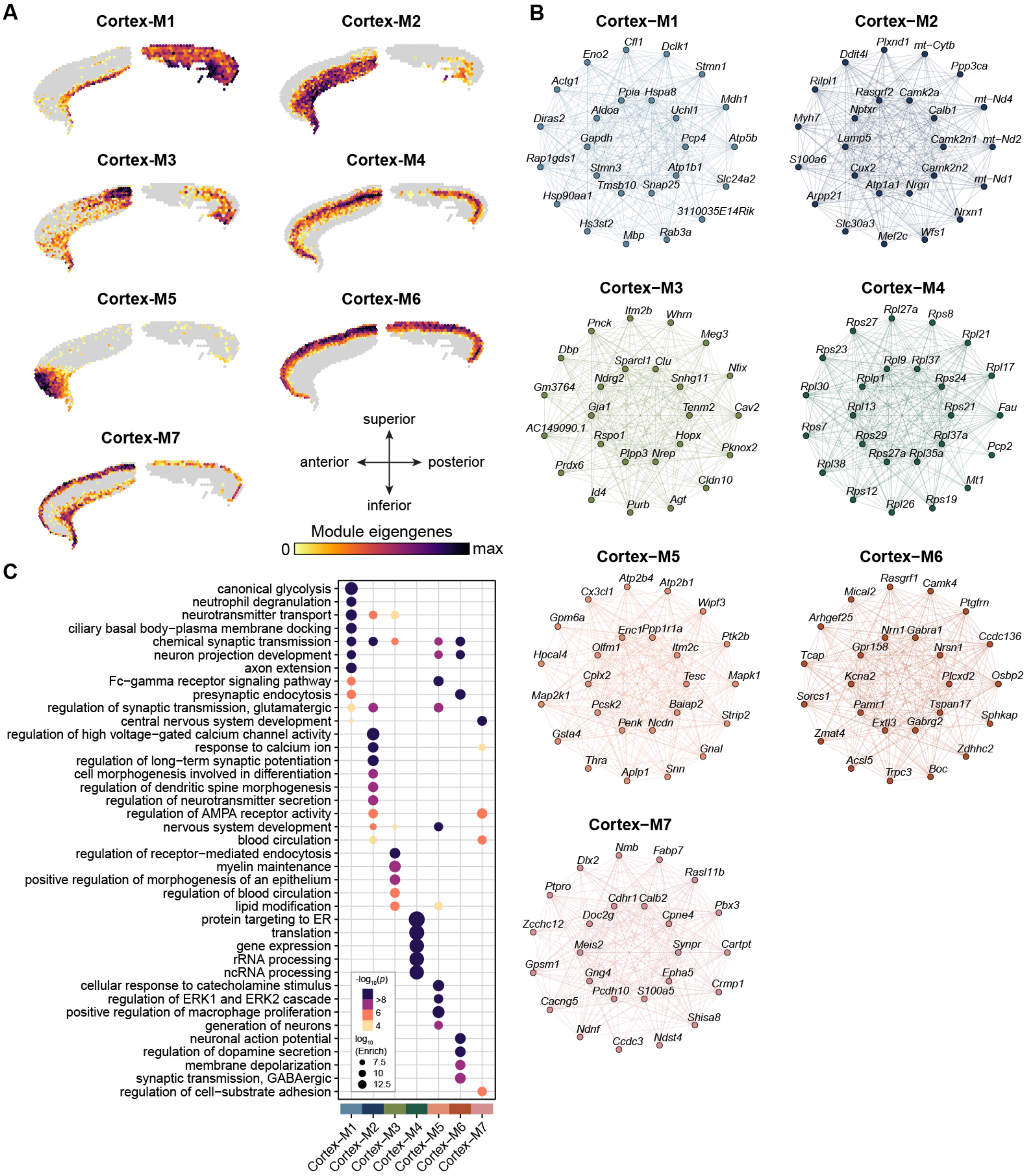

Figure S8. Mouse brain spatial transcriptomics co-expression network in cortical layers 2-6, related to Figure 3. A. ST samples colored by module eigengenes (MEs) for the seven cortical spatial co-expression modules. Grey color indicates a ME values less than zero. **B.** Hub gene networks for each cortical co-expression module. The top 25 hub genes ranked by kME are visualized. Nodes represent genes, and edges represent co-expression links. **C.** Dot plot showing selected GO term enrichment results for each co-expression module.

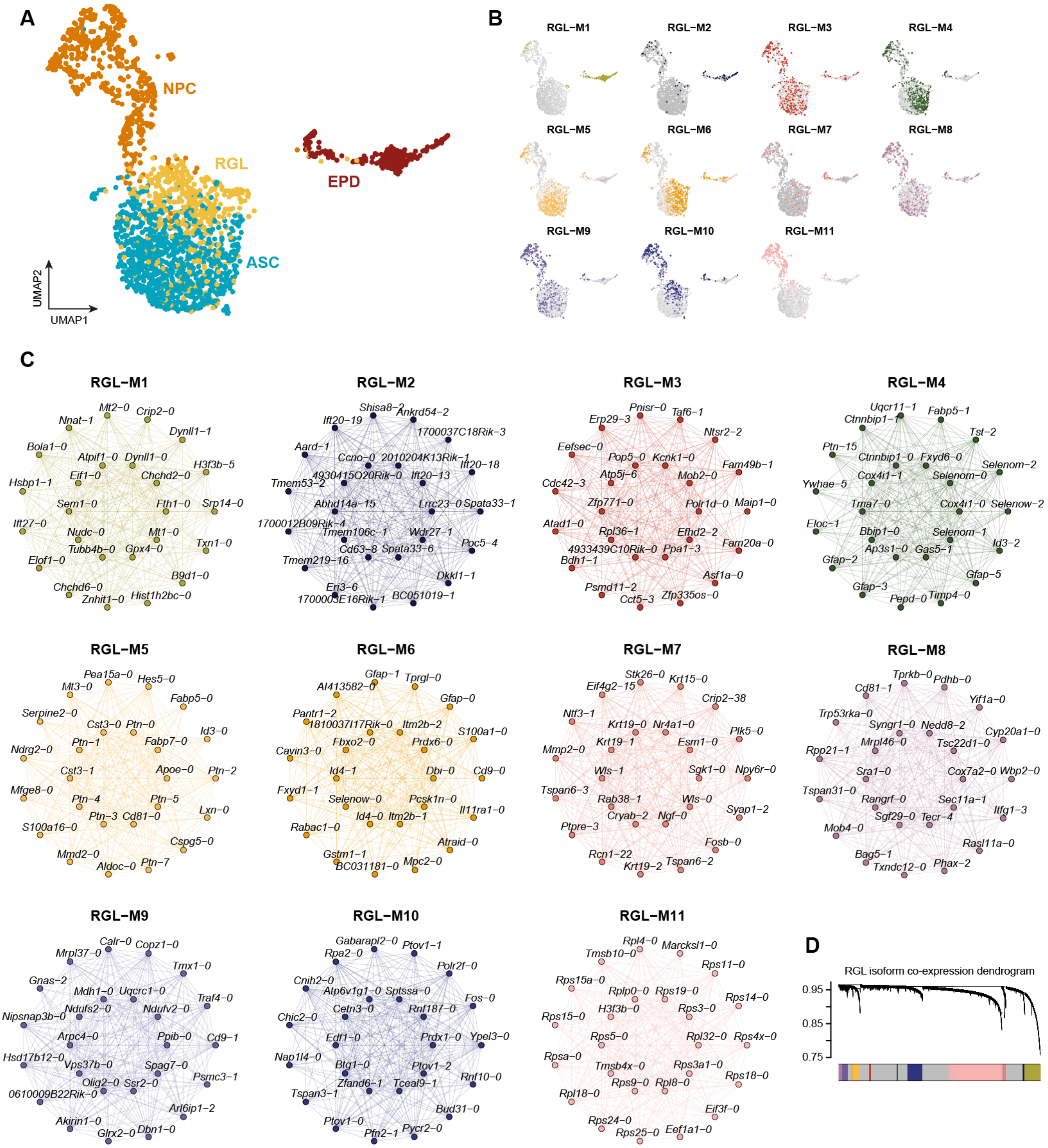

Figure S9. Isoform co-expression network analysis in the mouse hippocampus, related to Figure 4. A. UMAP plot of the radial glia lineage clusters from the mouse hippocampus ScISOrSeq dataset[9]. Cell type abbreviations are the following: ASC: astrocytes; EPD: ependymal cells; NPC: neuronal intermediate progenitor cells; RGL: radial glia. **B.** UMAP plots as in **a.** colored by MEiso for the eleven isoform co-expression modules. **C.** Hub isoform networks for each radial glia lineage co-expression modules. The top 25 hub genes ranked by kMEiso are visualized. Nodes represent isoforms, and edges represent co-expression links. **D.** Dendrogram showing the hierarchical clustering of isoforms into co-expression modules based on the topological overlap matrix (TOM).

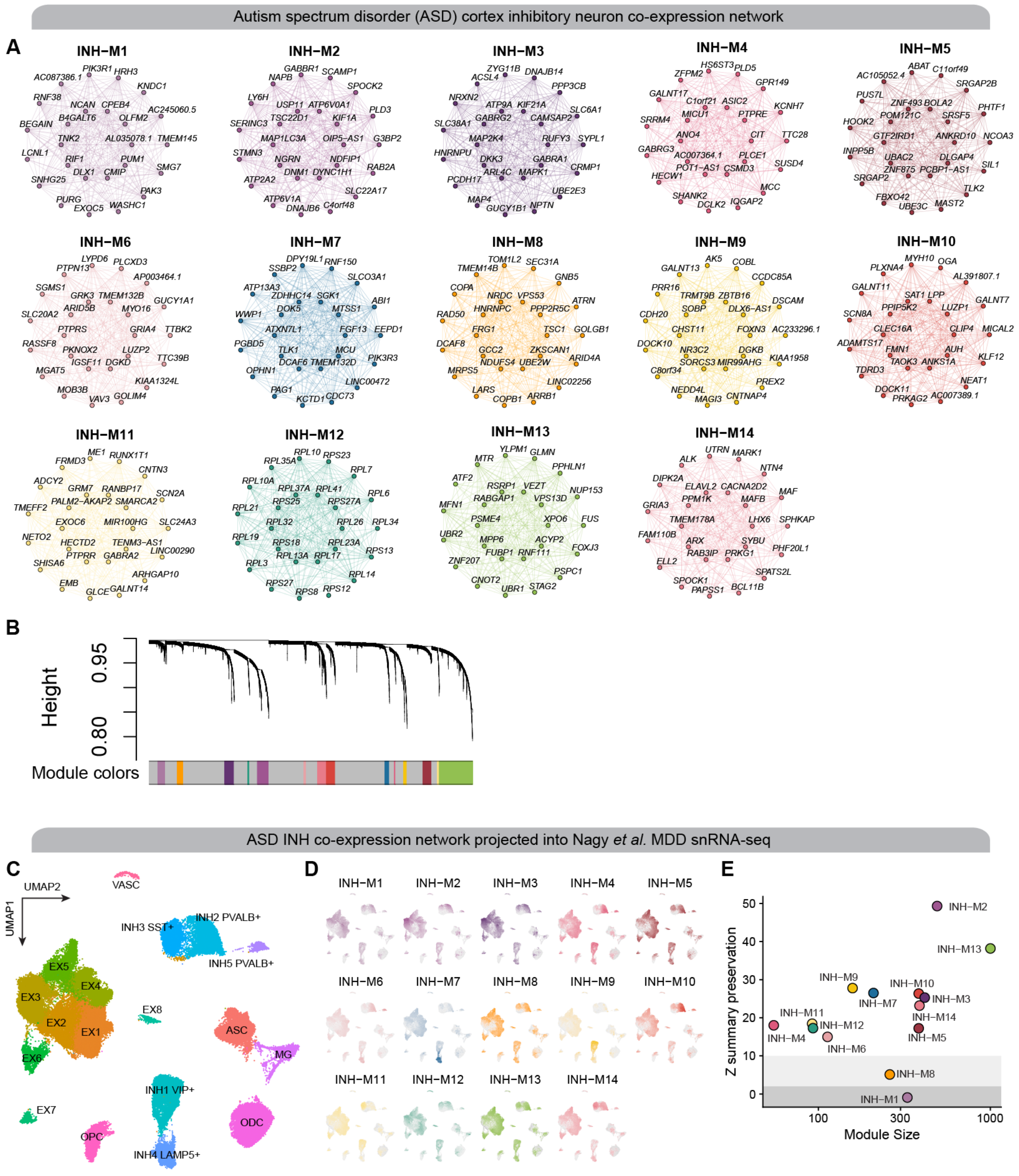

Figure S10. Co-expression network analysis of inhibitory neurons in Autism spectrum disorder, related to Figure 5. A. Hub gene networks for each inhibitory neuron co-expression modules. The top 25 hub genes ranked by kME are visualized. Nodes represent genes, and edges represent co-expression links. **B.** Dendrogram showing the hierarchical clustering of genes into co-expression modules based on the topological overlap matrix (TOM). **C.** UMAP plot of the snRNA-seq dataset of human major depressive disorder (MDD) [10]. Cells are colored by cluster annotations. Cell type abbreviations are the following: ASC: astrocytes; EX: excitatory neurons; INH: inhibitory neurons; MG: microglia; ODC: oligodendrocytes; OPC: oligodendrocyte progenitor cells; VASC: vascular cells. **D.** UMAP plots of the MDD dataset as in **C.** colored by the MEs projected from the ASD dataset. **E.** Module preservation statistics for the ASD inhibitory neuron modules in the inhibitory neuron population from the MDD dataset. Z-summary preservation < 2 indicates no evidence of module preservation, Z-summary preservation < 10 indicates moderate evidence of module preservation, and Z-summary preservation > 10 indicates high evidence of module preservation

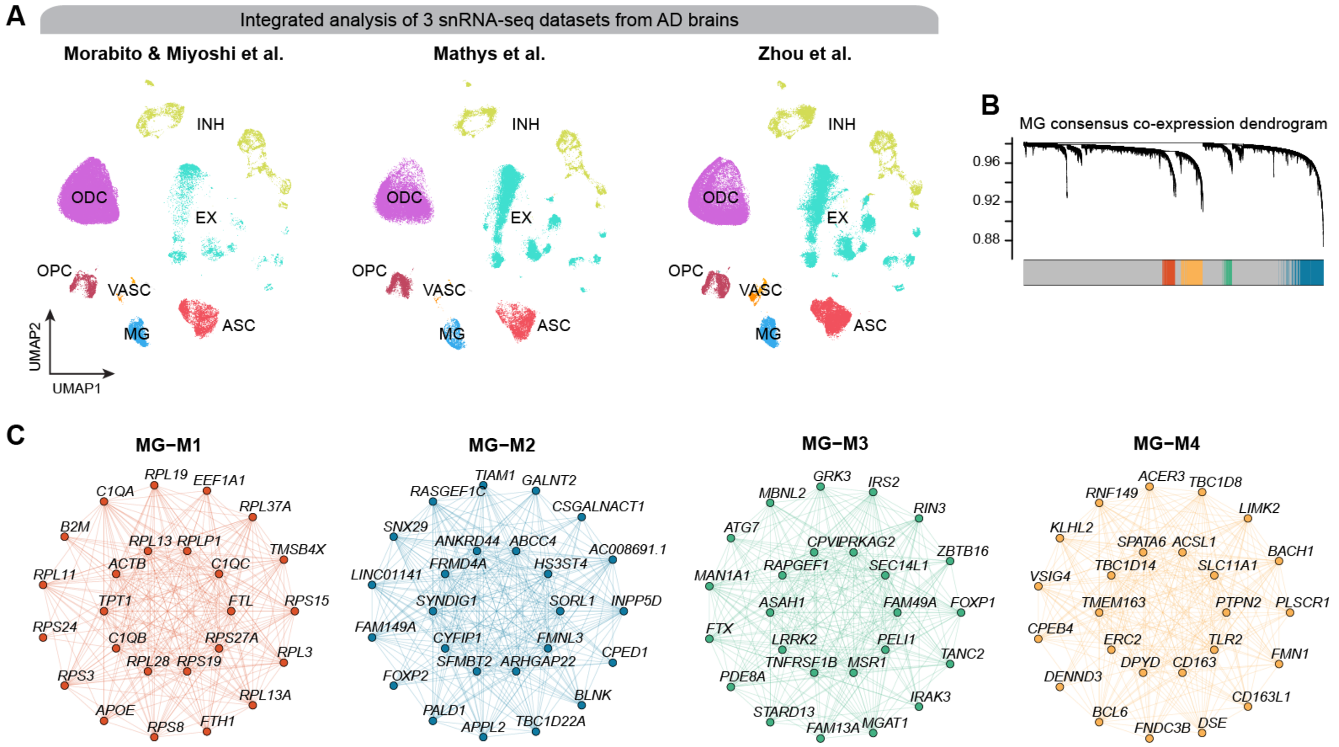

Figure S11. Consensus co-expression network analysis of microglia in Alzheimer's disease, related to Figure 6. A. UMAP plot of the integrated dataset from three AD snRNA-seq studies [7,11,12], colored by cell type assignment. Cell type abbreviations are the following: ASC: astrocytes; EX: excitatory neurons; INH: inhibitory neurons; MG: microglia; ODC: oligodendrocytes; OPC: oligodendrocyte progenitor cells; VASC: vascular cells. **B.** Dendrogram showing the hierarchical clustering of genes into co-expression modules based on the consensus topological overlap matrix (TOM) from the three snRNA-seq datasets. **C.** Hub gene networks for each microglia consensus co-expression modules. The top 25 hub genes ranked by kME are visualized. Nodes represent genes, and edges represent co-expression links.

References

1. Butler, A., Hoffman, P., Smibert, P., Papalexi, E., and Satija, R. (2018). Integrating single-cell transcriptomic data across different conditions, technologies, and species. Nature Biotechnology *36*, 411–420. doi:10.1038/nbt.4096.

2. Stuart, T., Butler, A., Hoffman, P., Hafemeister, C., Papalexi, E., Mauck, W. M., Hao, Y., Stoeckius, M., Smibert, P., and Satija, R. (2019). Comprehensive Integration of Single-Cell Data. Cell *177*, 1888–1902.e21. doi:10.1016/j.cell.2019.05.031.

3. Hao, Y., Hao, S., Andersen-Nissen, E., Mauck, W. M., Zheng, S., Butler, A., Lee, M. J., Wilk, A. J., Darby, C., Zager, M., Hoffman, P., Stoeckius, M., Papalexi, E., Mimitou, E. P., Jain, J., Srivastava, A., Stuart, T., Fleming, L. M., Yeung, B., Rogers, A. J., McElrath, J. M., Blish, C. A., Gottardo, R., Smibert, P., and Satija, R. (2021). Integrated analysis of multimodal single-cell data. Cell *184*, 3573–3587.e29. doi:10.1016/j.cell.2021.04.048.

4. Wolf, F. A., Angerer, P., and Theis, F. J. (2018). SCANPY: large-scale single-cell gene expression data analysis. Genome Biology *19*, 15. doi:10.1186/s13059-017-1382-0.

5. Persad, S., Choo, Z.-N., Dien, C., Sohail, N., Masilionis, I., Chaligné, R., Nawy, T., Brown, C. C., Sharma, R., Pe'er, I., Setty, M., and Pe'er, D. (2023). SEACells infers transcriptional and epigenomic cellular states from single-cell genomics data. Nature Biotechnology (1–12). doi:10.1038/s41587-023-01716-9.

6. Plaisier, S. B., Taschereau, R., Wong, J. A., and Graeber, T. G. (2010). Rank–rank hypergeometric overlap: identification of statistically significant overlap between gene-expression signatures. Nucleic Acids Research *38*, e169–e169. doi:10.1093/nar/gkq636.

7. Zhou, Y., Song, W. M., Andhey, P. S., Swain, A., Levy, T., Miller, K. R., Poliani, P. L., Cominelli, M., Grover, S., Gilfillan, S., Cella, M., Ulland, T. K., Zaitsev, K., Miyashita, A., Ikeuchi, T., Sainouchi, M., Kakita, A., Bennett, D. A., Schneider, J. A., Nichols, M. R., Beausoleil, S. A., Ulrich, J. D., Holtzman, D. M., Artyomov, M. N., and Colonna, M. (2020). Human and mouse single-nucleus transcriptomics reveal TREM2-dependent and TREM2-independent cellular responses in Alzheimer's disease. Nature Medicine *26*, 131–142. doi:10.1038/s41591-019-0695-9.

8. Shabestari, S. K., Morabito, S., Danhash, E. P., McQuade, A., Sanchez, J. R., Miyoshi, E., Chadarevian, J. P., Claes, C., Coburn, M. A., Hasselmann, J., Hidalgo, J., Tran, K. N., Martini, A. C., Rothermich, W. C., Pascual, J., Head, E., Hume, D. A., Pridans, C., Davtyan, H., Swarup, V., and Blurton-Jones, M. (2022). Absence of microglia promotes diverse pathologies and early lethality in Alzheimer's disease mice. Cell Reports *39*, 110961. doi:10.1016/j.celrep.2022.110961.

9. Joglekar, A., Prjibelski, A., Mahfouz, A., Collier, P., Lin, S., Schlusche, A. K., Marrocco, J., Williams, S. R., Haase, B., Hayes, A., Chew, J. G., Weisenfeld, N. I., Wong, M. Y., Stein, A. N., Hardwick, S. A., Hunt, T., Wang, Q., Dieterich, C., Bent, Z., Fedrigo, O., Sloan, S. A., Risso, D., Jarvis, E. D., Flicek, P., Luo, W., Pitt, G. S., Frankish, A., Smit, A. B., Ross, M. E., and Tilgner, H. U. (2021). A spatially resolved brain region- and cell type-specific isoform atlas of the postnatal mouse brain. Nature Communications *12*, 463. doi:10.1038/s41467-020-20343-5.

10. Nagy, C., Maitra, M., Tanti, A., Suderman, M., Théroux, J.-F., Davoli, M. A., Perlman, K., Yerko, V., Wang, Y. C., Tripathy, S. J., Pavlidis, P., Mechawar, N., Ragoussis, J., and Turecki, G. (2020). Single-nucleus transcriptomics of the prefrontal cortex in major depressive disorder implicates oligodendrocyte precursor cells and excitatory neurons. Nature Neuroscience *23*, 771–781. doi:10.1038/s41593-020-0621-y.

11. Morabito, S., Miyoshi, E., Michael, N., Shahin, S., Martini, A. C., Head, E., Silva, J., Leavy, K., Perez-Rosendahl, M., and Swarup, V. (2021). Single-nucleus chromatin accessibility and transcriptomic characterization of Alzheimer's disease. Nature Genetics *53*, 1143–1155. doi:10.1038/s41588-021-00894-z.

12. Mathys, H., Davila-Velderrain, J., Peng, Z., Gao, F., Mohammadi, S., Young, J. Z., Menon, M., He, L., Abdurrob, F., Jiang, X., Martorell, A. J., Ransohoff, R. M., Hafler, B. P., Bennett, D. A., Kellis, M., and Tsai, L.-H. (2019). Single-cell transcriptomic analysis of Alzheimer's disease. Nature *570*, 332–337. doi:10.1038/s41586-019-1195-2.
