# Supplementary material for: hdWGCNA identifies co-expression networks in high-dimensional transcriptomics data
Source: Cell Rep Methods. 2023 Jun 12;3(6):100498. doi: 10.1016/j.crmeth.2023.100498 (PMC10326379; doi:10.1016/j.crmeth.2023.100498)
Supplement: Document S2. Article plus supplemental information [file mmc5.pdf]

Cell Reports
Methods

Article

hdWGCNA identifies co-expression networks in high-dimensional transcriptomics data

Graphical abstract

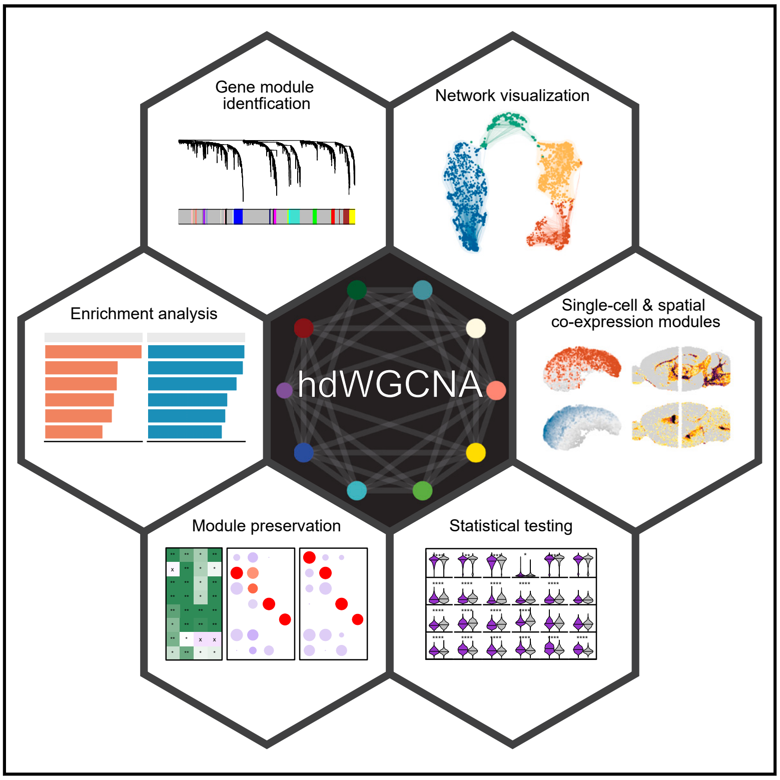

Authors

Samuel Morabito, Fairlie Reese, Negin Rahimzadeh, Emily Miyoshi, Vivek Swarup

Correspondence

vswarup@uci.edu

In brief

Morabito et al. present hdWGCNA, an open-source R package for gene co-expression network analysis in single-cell and spatial transcriptomics data. hdWGCNA builds networks of genes using correlation information in specific cell subpopulations and spatial domains. Applications of hdWGCNA in autism spectrum disorder and Alzheimer's disease revealed disease-associated gene networks.

Highlights

- hdWGCNA constructs co-expression networks in high-dimensional transcriptomics data
- hdWGCNA provides tools for statistics, visualization, and downstream interpretation
- hdWGCNA is an open-source R package that uses Seurat data structures
- hdWGCNA in human diseases demonstrates real-world analysis in complex datasets

Morabito et al., 2023, Cell Reports Methods *3*, 100498
June 26, 2023 © 2023 The Author(s).
https://doi.org/10.1016/j.crmeth.2023.100498

CellPress

Cell Reports Methods

CellPress
OPEN ACCESS

Article

hdWGCNA identifies co-expression networks in high-dimensional transcriptomics data

Samuel Morabito,[1,2,3] Fairlie Reese,[2,4] Negin Rahimzadeh,[1,2,3] Emily Miyoshi,[3,5] and Vivek Swarup[2,3,5,6,*]
[1]Mathematical, Computational, and Systems Biology (MCSB) Program, University of California, Irvine, Irvine, CA, USA
[2]Center for Complex Biological Systems (CCBS), University of California, Irvine, Irvine, CA, USA
[3]Institute for Memory Impairments and Neurological Disorders (MIND), University of California, Irvine, Irvine, CA, USA
[4]Department of Developmental and Cell Biology, University of California, Irvine, Irvine, CA, USA
[5]Department of Neurobiology and Behavior, University of California, Irvine, Irvine, CA, USA
[6]Lead contact
*Correspondence: vswarup@uci.edu
https://doi.org/10.1016/j.crmeth.2023.100498

MOTIVATION Single-cell and spatial transcriptomics assays are commonly used to profile the molecular signatures of biological systems, yielding high-dimensional datasets that can be used to model gene regulation across cell types, cell states, and spatial niches. Many statistical tools for high-dimensional transcriptomics data analysis focus on individual features rather than the underlying network structure, ignoring potential interactions between transcripts or genes. Here, we introduce hdWGCNA, a comprehensive methodological framework for the inference, analysis, and interpretation of gene co-expression networks in high-dimensional transcriptomics data. hdWGCNA is implemented as an open-source R package that extends the Seurat ecosystem of data analysis tools.

SUMMARY

Biological systems are immensely complex, organized into a multi-scale hierarchy of functional units based on tightly regulated interactions between distinct molecules, cells, organs, and organisms. While experimental methods enable transcriptome-wide measurements across millions of cells, popular bioinformatic tools do not support systems-level analysis. Here we present hdWGCNA, a comprehensive framework for analyzing co-expression networks in high-dimensional transcriptomics data such as single-cell and spatial RNA sequencing (RNA-seq). hdWGCNA provides functions for network inference, gene module identification, gene enrichment analysis, statistical tests, and data visualization. Beyond conventional single-cell RNA-seq, hdWGCNA is capable of performing isoform-level network analysis using long-read single-cell data. We showcase hdWGCNA using data from autism spectrum disorder and Alzheimer's disease brain samples, identifying disease-relevant co-expression network modules. hdWGCNA is directly compatible with Seurat, a widely used R package for single-cell and spatial transcriptomics analysis, and we demonstrate the scalability of hdWGCNA by analyzing a dataset containing nearly 1 million cells.

INTRODUCTION

The development and widespread adoption of single-cell and spatial genomics approaches has led to routine generation of high-dimensional datasets in a variety of biological systems. These technologies are frequently used to study developmental stages, evolutionary trajectories, disease states, drug perturbations, and other experimental conditions. Despite the inherent complexity and interconnectedness of biological systems, studies leveraging single-cell and spatial genomics typically analyze individual features (genes, isoforms, proteins, etc.) one by one, greatly oversimplifying the underlying biology. These datasets provide an opportunity for investigating and quantifying the relationships between these features to further contextualize their roles across biological conditions of interest.

Here we developed hdWGCNA, a framework for co-expression network analysis[1] in single-cell and spatial transcriptomics data. Co-expression networks are based on transformed pairwise correlations of input features, resulting in a quantitative measure of relatedness between genes.[1,2] Hierarchical clustering on the network structure allows us to uncover functional modules of genes whose expression profiles are tightly intertwined,[3,4] which typically correspond to specific biological processes and disease states. Considering that unique cell types and cell states have distinct gene expression programs, we designed hdWGCNA to facilitate multi-scale analysis of cellular and

© 2023 The Author(s).
This is an open access article under the CC BY license (http://creativecommons.org/licenses/by/4.0/).

spatial hierarchies. hdWGCNA provides a rich suite of functions for data analysis and visualization, providing biological context for co-expression networks by leveraging a variety of biological knowledge databases. To maximize usability among the genomics community, the hdWGCNA R package extends the data structures and functionality of the widely used Seurat package,[5–7] and we developed an extensive documentation website for hdWGCNA demonstrating its use on new datasets. Further, we used hdWGCNA to analyze a single-cell RNA sequencing (scRNA-seq) dataset consisting of 1 million cells, showcasing the scalability of hdWGCNA in large datasets.

In this study, we applied hdWGCNA in a variety of high-dimensional transcriptomics datasets from different technologies and biological conditions. As a common use case, we first performed iterative network analysis of the major cell types in the human prefrontal cortex (PFC), identifying shared and specific network modules in each cell type. We constructed co-expression networks in anterior and posterior mouse brain sections profiled with 10× Genomics Visium spatial transcriptomics (ST), and found distinct spatial patterns of these gene expression programs. Using long-read scRNA-seq data from the mouse hippocampus,[8] we uncovered splicing isoform co-expression networks in the radial glia lineage involved in cell fate specification. Network analysis of inhibitory neurons from published single-nucleus RNA sequencing (snRNA-seq) in autism spectrum disorder (ASD) donors[9] revealed modules disrupted in ASD containing key genetic risk genes such as *SCN2A*, *TSC1*, and *SHANK2*. We performed consensus co-expression network analysis of microglia from three Alzheimer's disease (AD) snRNA-seq studies,[10–12] yielding multiple gene modules corresponding to disease-associated microglia and polygenic risk of AD. Finally, we used hdWGCNA to project gene modules from two bulk RNA-seq studies of AD patients into an snRNA-seq dataset of the AD brain, showing that our approach allows for interrogation of gene modules and networks that have been previously identified.

RESULTS

Constructing co-expression networks from high-dimensional transcriptomics data

Here we describe hdWGCNA, a comprehensive framework for constructing and analyzing co-expression networks in high-dimensional transcriptomic data (Figure 1A). Given a gene expression dataset as input, co-expression network analysis typically consists of the following analysis steps: computing pairwise correlations of input features, weighting correlations with a soft-power threshold (β), computing the topological overlap between features, and unsupervised clustering via the Dynamic Tree Cut algorithm[3] (Figure S1 and STAR Methods). The sparsity and noise inherent in single-cell data can lead to spurious gene-gene correlations, thereby complicating co-expression network analysis. Additionally, the correlation structure of single-cell or spatial transcriptomic data varies greatly for different subsets (cell types, cell states, anatomical regions). A typical hdWGCNA workflow in scRNA-seq data accounts for these considerations by collapsing highly similar cells into "metacells" to reduce sparsity while retaining cellular heterogeneity and by allowing for a modular design to perform separate network analyses in specified cell populations.

Metacells are defined as small groups of transcriptomically similar cells representing distinctive cell states. There are several approaches to identify metacells from single-cell genomics data.[13–16] We leverage a bootstrapped aggregation (bagging) algorithm for constructing metacell transcriptomic profiles from single-cell datasets by applying K-nearest neighbors (KNN) to a dimensionality-reduced representation of the input dataset (STAR Methods, Algorithm 1). This approach can be performed for each biological replicate to ensure that critical information about each sample (age, sex, disease status, etc.) is retained for downstream analysis. We computed gene-gene correlations in the normalized gene expression matrix from the single-cell dataset and metacell expression matrices while varying the number of cells to collapse into a single metacell (the KNN *K* parameter). The distribution of these gene-gene correlations displays a spike at zero for the single-cell expression matrix, with flattened distributions corresponding to more non-zero correlations in the metacell matrices, indicating that metacell expression profiles are less prone to noisy gene-gene correlations compared with the single-cell matrix (Figure 1B) (STAR Methods). We note that sparsity (defined in Equation 1) is greatly reduced in the metacell matrices for each cell type compared with the single-cell matrices, with over a 10-fold reduction in some cases (Figure 1C). We applied hdWGCNA to a dataset of $CD34^+$ hematopoietic stem and progenitor stem cells[16] using two additional metacell approaches[14,16] and found that all approaches were suitable for downstream network analysis (Figure S2; Data S1). Metacell algorithms strive to retain biologically meaningful signals spanning a spectrum of cell states in a tissue of interest; therefore, it is necessary to carefully apply these approaches to avoid obscuring these cell states. For example, the hdWGCNA metacell algorithm requires a dimensional reduction of the input expression matrix, but these reductions often contain technical artifacts. The choice of dimensionality reduction method and handling of technical artifacts would then influence the effectiveness of metacell construction. Further, the optimal number of cells to merge together to form a single metacell may differ across cell types and tissues, attempting to balance between increasing information content of the aggregated group while avoiding merging of dissimilar cells. Aside from metacell approaches, pseudo-bulk aggregation of all cells in a given population have yielded favorable results in benchmarks of differential gene expression tests,[17] suggesting that, given a sufficient sample size, pseudo-bulk expression profiles are likely suitable for co-expression network analysis.

While co-expression modules consist of many genes, it is convenient to summarize the expression of the entire module into a single metric. Module eigengenes (MEs), defined as the first principal component of the module's gene expression matrix (STAR Methods, Algorithm 2), describe the expression patterns of entire co-expression modules. hdWGCNA computes MEs using specific accommodations for high-dimensional data, allowing for batch correction and regression of continuous covariates (STAR Methods, Algorithm 2). Optionally, hdWGCNA can use alternative gene scoring methods such as or UCell[18] or

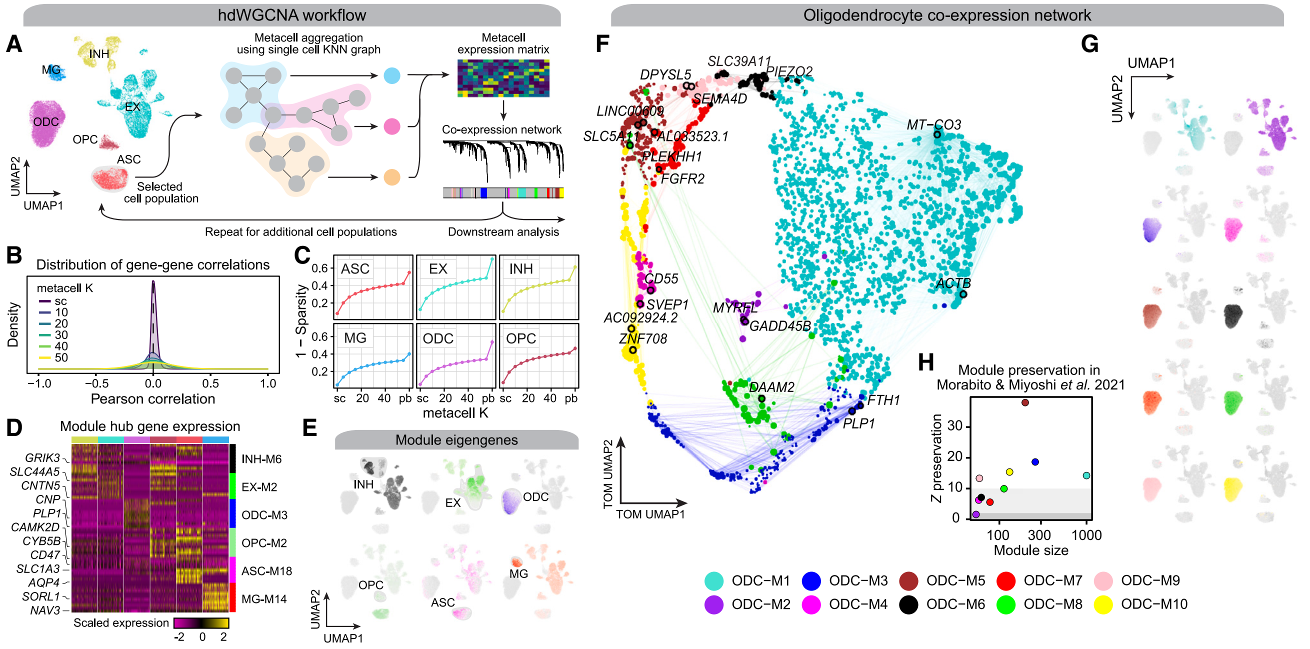

Figure 1. Overview of the hdWGCNA workflow and application in the human prefrontal cortex

(A) Schematic overview of the standard hdWGCNA workflow on a scRNA-seq dataset. UMAP plot shows 36,671 cells from 11 cognitively normal donors in the Zhou et al. human prefrontal cortex (PFC) dataset. ASC, astrocytes; EX, excitatory neurons; INH, inhibitory neurons; MG, microglia; ODC, oligodendrocytes; OPC, oligodendrocyte progenitor cells.

(B) Density plot showing the distribution of pairwise Pearson correlations between genes from the single-cell (sc) expression matrix and metacell expression matrices with varying values of the *K*-nearest neighbors parameter *K*.

(C) Expression matrix density (1, sparsity) for the sc, pseudo-bulk (pb), and metacell matrices with varying values of *K* in each cell type.

(D) Heatmap of scaled gene expression for the top five hub genes by kME in INH-M6, EX-M2, ODC-M3, OPC-M2, ASC-M18, and MG-M14.

(E) snRNA-seq UMAP colored by module eigengene (ME) for selected modules as in (D).

(F) UMAP plot of the ODC co-expression network. Each node represents a single gene, and edges represent co-expression links between genes and module hub genes. Point size is scaled by kME. Nodes are colored by co-expression module assignment. The top two hub genes per module are labeled. Network edges were downsampled for visual clarity.

(G) snRNA-seq UMAP as in (A) colored by MEs for the 10 ODC co-expression modules as in (F).

(H) Module preservation analysis of the ODC modules in the Morabito et al.[12] human PFC dataset. The module's size versus the preservation statistic (*Z* preservation) is shown for each module. $Z < 5$, not preserved; $10 > Z \geq 5$, moderately preserved; $Z \geq 10$, highly preserved.

Seurat's AddModuleScore function, and we show that these scores are correlated with MEs (Figure S3).

We demonstrate hdWGCNA in single-cell transcriptomic data through an iterative network analysis of six major cell types in the Zhou et al. human PFC snRNA-seq dataset of 11 cognitively normal donors (Figure 1A).[11] We constructed metacells and performed co-expression network analysis for each major cell type in the human PFC dataset[11] using the standard hdWGCNA workflow, yielding distinct network structures and sets of gene modules (Data S2 and S3). Networks were constructed using metacell expression matrices for each cell type separately, but we computed MEs for each module using the entire snRNA-seq dataset, allowing us to interrogate the cell-type specificity of these modules' expression programs across all cell types. This iterative network analysis revealed 96 co-expression modules across the six major cell types. Through differential module eigengene (DME) analysis, we found shared and distinct module expression patterns across different cell types (Data S2; STAR Methods), and we highlight specific modules from each cell type (Figures 1D and 1E). Further, we performed a pairwise gene set overlap analysis of the 96 co-expression modules, and, while we did find that some modules had significant overlaps across the different cell types, the gene sets comprising these modules were overall quite distinct, with a maximum Jaccard index between two modules of 0.297 and a median of 0.005 (STAR Methods and Figure S4). The expression of module hub genes, which are highly connected members of the co-expression network ranked by eigengene-based connectivity (kME), tend to display cell-type-specific patterns, such as the myelination genes *CNP* and *PLP1* in oligodendrocyte (ODC) module ODC-M3 (Figure 1D). However, some co-expression modules may correspond to cellular processes common to multiple cell types, in which case the hub genes may be widely expressed. We inspected the MEs of selected cell-type-specific modules and found that the overall expression patterns were similar to that of their constituent hub genes (Figures 1D and 1E).

We showcase some of the downstream functionalities of hdWGCNA using the ODC co-expression network (Figures 1F–1H). For network visualization, we used Uniform Manifold Approximation and Projection (UMAP)[19] to embed the co-expression

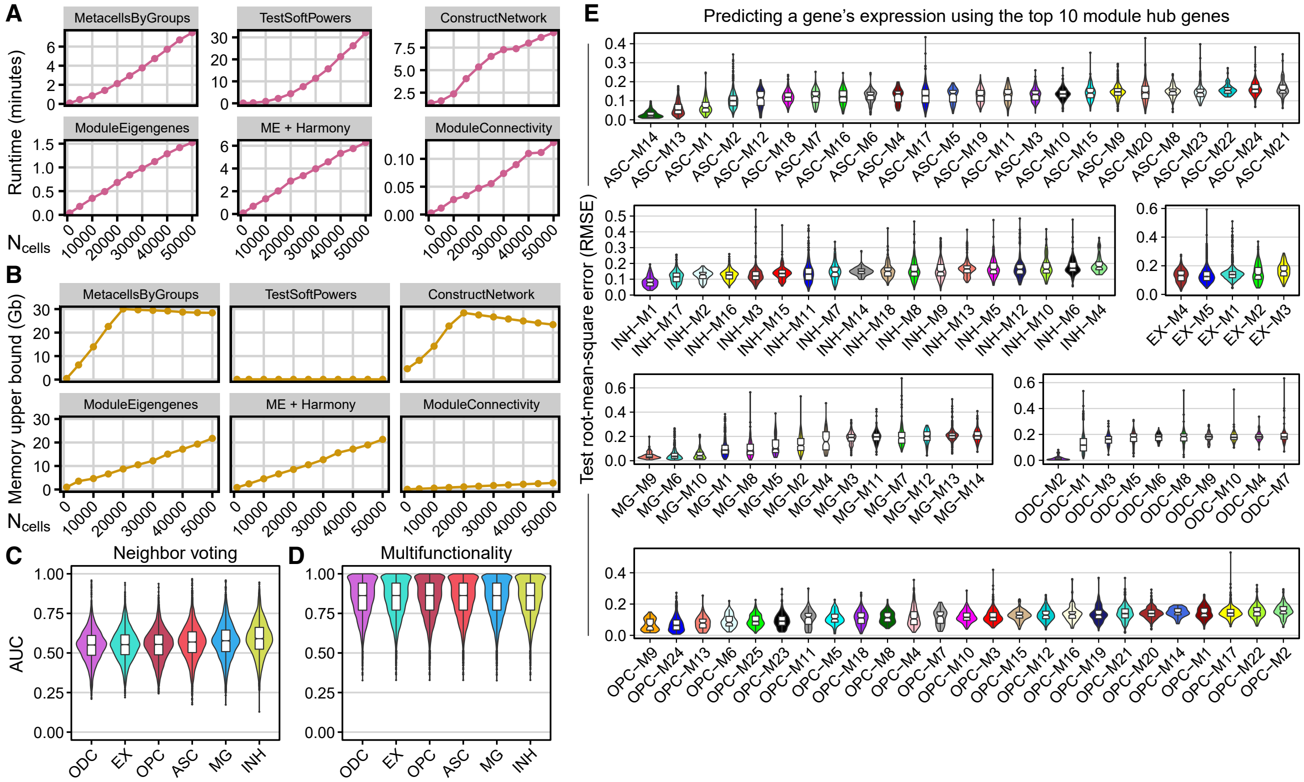

Figure 2. Runtime, memory usage, and performance of hdWGCNA

(A and B) We ran the main co-expression network analysis functions of the hdWGCNA R package on 65,415 neuronal cells in a human brain dataset[9] from 54 samples, and tracked the runtime (A) and memory usage upper bound (B) for different-sized subsets of the data ranging from 1,000 through 50,000 cells.

(C) Violin plots showing distributions of EGAD[23] neighbor-voting area under the receiver operating characteristic curve (AUC) scores in each of the cell-type-specific co-expression networks from the human PFC dataset.[11]

(D) Violin plots showing distributions of multifunctionality AUC scores in each of the cell-type-specific co-expression networks from the human PFC dataset. ASC, astrocytes; EX, excitatory neurons; INH, inhibitory neurons; MG, microglia; ODC, oligodendrocytes; OPC, oligodendrocyte progenitor cells.

(E) Performance of the XGBoost regularized regression models used to predict gene expression based on the expression of the top 10 module hub genes for all 96 co-expression modules from the Zhou et al.[11] human PFC dataset. Violin plots showing the test set root-mean-square error (RMSE) comparing the predicted expression with observed for each gene, split by each co-expression module. Modules are ordered within each cell type from lowest mean RMSE to highest.

network topological overlap matrix (TOM) into a two-dimensional manifold, using the topological overlap of each gene with the top hub genes from each module as input features (STAR Methods; Figure 1F). We found that eight of the 10 ODC modules were specifically expressed in ODC cells based on their MEs (Figure 1G; Wilcoxon rank-sum test Bonferroni-adjusted p <0.05). Finally, we performed module preservation analysis[20] to test the reproducibility of these modules in an independent dataset[12] and found that all of the ODC-specific modules were significantly preserved (Z summary preservation ≥ 5). In sum, these network analyses in the human PFC dataset shows the core capabilities of the hdWGCNA workflow (Figure S1). Finally, we performed a similar iterative network analysis on a peripheral blood mononuclear cell (PBMC) scRNA-seq dataset of nearly 1M cells, highlighting the scalability of hdWGCNA in large datasets (Figure S5; Data S1).

Runtime, memory usage, and evaluation of hdWGCNA

We measured the runtime and memory usage of hdWGCNA as a function of the number of input cells. Using the 65,415 neuronal cells from the Velmeshev et al.[9] human PFC snRNA-seq dataset (54 samples), we ran hdWGCNA on different-sized subsets ranging from 1,000 to 50,000 cells to test the runtime and memory consumption of the main network analysis steps (Figures 2A and 2B). We report the memory upper bound in gigabytes measured throughout the duration of each function. The runtime of the MetacellsByGroups function increased steadily with the number of cells, but the memory usage plateaus. This function attempts to construct a target number of metacells within each biological replicate and each cell population, and the algorithm terminates early if this target is reached, thus explaining the plateau in the memory usage graph. While TestSoftPowers generally had a low memory footprint, it was the slowest individual function based on these tests. Importantly, TestSoftPowers can be sped up by using a subset of the data, or by testing fewer soft-power thresholds than the default. The efficiency of ConstructNetwork varies both with the number of input cells and features, where this calculation will slow down as more cells and features are included. ModuleEigengenes uses the implicitly restarted Lanczos bidiagonalization algorithm (IRLBA)[21] for fast

singular value decomposition (SVD) of sparse matrices, and the runtime and memory usage of this function both linearly increase with the number of cells in the dataset. Optionally, ModuleEigengenes can employ the harmony[22] algorithm following SVD, which increases runtime but not memory usage. Further, the efficiency of this function varies with the number of co-expression modules detected and with the number of features in each modules. Finally, ModuleConnectivity computes eigengene-based connectivity as product-moment correlation coefficients between the sparse gene expression matrix and the MEs matrix, which resulted in fast calculations with low memory usage.

We next sought to evaluate the co-expression networks identified by hdWGCNA using a functional coherence analysis. We used the EGAD neighbor-voting algorithm[23] to predict known biological pathway associations of genes based on the co-expression network structure using the cell-type-specific co-expression networks from the Zhou et al.[11] human PFC dataset. In principle, we expect that co-expressed genes are involved in similar biological processes, and therefore co-expression network structures should be predictive of biological pathway membership. Briefly, EGAD performs a 3-fold cross-validation classification by occluding the pathway labels of a subset of genes and then attempting to predict the pathway membership of those occluded genes based on their labeled neighbors in the network. We used EGAD to test the functional coherence of our hdWGCNA co-expression networks for a set of Gene Ontology (GO) terms, reporting area under the receiver operating characteristic curve (AUC) value for each term (Figure 2C). We report a similar level of functional coherence in these co-expression networks to a previous study that evaluated co-expression networks derived from scRNA-seq data with different measures of gene-gene association.[24] The inhibitory neuron network performed the best for functional coherence with a median neighbor-voting AUC of 0.592, while the lowest-performing network was from the oligodendrocytes with a median AUC of 0.549. We tested whether there was a bias toward genes that were multifunctional based on the frequency that they appeared in the annotated set of GO terms, and we found that multifunctional genes did not bias the co-expression functional coherence results (Figure 2D).

In principle, genes within the same co-expression modules derived from specific cell types should be functionally related or co-regulated. The expression of module hub genes, which exhibit the highest intramodular connectivity, may be predictive of the expression of other module member genes if the network is well defined and contains meaningful structures. For each of the 96 cell-type PFC co-expression modules, we sought to predict the expression of each gene using the top 10 module hub genes as the input features to a XGBoost[25] regularized regression model. In this analysis, we performed 5-fold cross-validation, and we report the performance as root-mean-square error (RMSE) of the test set averaged over each fold (Figure 2E). Overall, we found that module hub gene expression was generally predictive of module member gene expression across all modules in the six cell-type co-expression networks, where the module with the best performance had an average test set RMSE of 0.0159 and the module with the worst performance had an average test set RMSE of 0.209 (Figure 2E). This analysis and our functional coherence analysis provide support that hdWGCNA co-expression networks and gene modules capture biologically relevant information in specific cell types.

Spatial co-expression networks represent regional expression patterns in the mouse brain

ST enables the investigation of biological patterns that might otherwise be hidden in other -omics technologies, such as scRNA-seq or bulk RNA-seq.[26,27] We used hdWGCNA to identify spatial co-expression network modules in the murine brain using a publicly available Visium transcriptomics dataset from 10× Genomics (Figure 3A). This ST dataset consists of one posterior and one anterior slice originating from a sagittal brain section from a single male mouse at 8 weeks of age. Sequencing-based ST approaches such as Visium yield transcriptome-wide gene expression profiles localized to individual "spots" where a single spot likely contains multiple cells, and this dataset is composed of 2,696 spots in the anterior slice and 3,353 spots in the posterior slice. Data sparsity is also inherent to the current generation of these technologies, therefore we propose a metaspot aggregation approach prior to network analysis (Figure S6). Evenly spaced spots throughout the input ST slide are used as principal spots, with at least one other spot in between two principal spots. The transcriptomes of the principal spots and their direct neighbors are aggregated into metaspot expression profiles, containing at most seven ST spots (Figure S6A). Similar to metacells in scRNA-seq, the sparsity of the metaspot expression matrix was reduced compared with the original ST matrix (Figure S6B), and the distribution of gene-gene correlations in the metaspot expression matrix was less concentrated at zero (Figure S6C). hdWGCNA is capable of processing any number of ST samples in the same co-expression network analysis by constructing metaspots separately for each sample.

We applied hdWGCNA in the mouse brain Visium dataset, identifying 12 spatial modules (SM1-12; Figure S7; Data S1), and we embedded the co-expression network in two dimensions using UMAP (Figure 3B). DME analysis showed that spatial co-expression modules displayed distinct regional expression profiles based on their MEs (Figure 3C; Data S3), encompassing a wide array of cellular processes such as the myelination module SM1 in the white matter tracts, and synaptic transmission modules SM7, SM9, SM11, and SM12 (Figure S7C; Data S2). For example, DME analysis showed that expression of SM4 was localized to the ventricles and cortical layer 1 near the blood-brain barrier (Figure 3C). Further, the hub genes of SM4 include hemoglobin subunits (*Hba-a1*, *Hba-a2*, *Hbb-bt*), and we show that SM4 was enriched for biological processes associated with brain vasculature (Figures 3B and S7C). We compared these gene modules with cluster marker genes from a whole-mouse-brain snRNA-seq dataset[28] and found significant correspondences, such as the striatum module SM7 and medium spiny neurons (Fisher's exact test false discovery rate [FDR] <0.05; Figure S7D). Additionally, we performed network analysis on a subset of this dataset containing cortical layers 2–6 (Figure S8), identifying additional fine-grained spatial co-expression modules localized to specific cortical layers (Data S1 and S2).

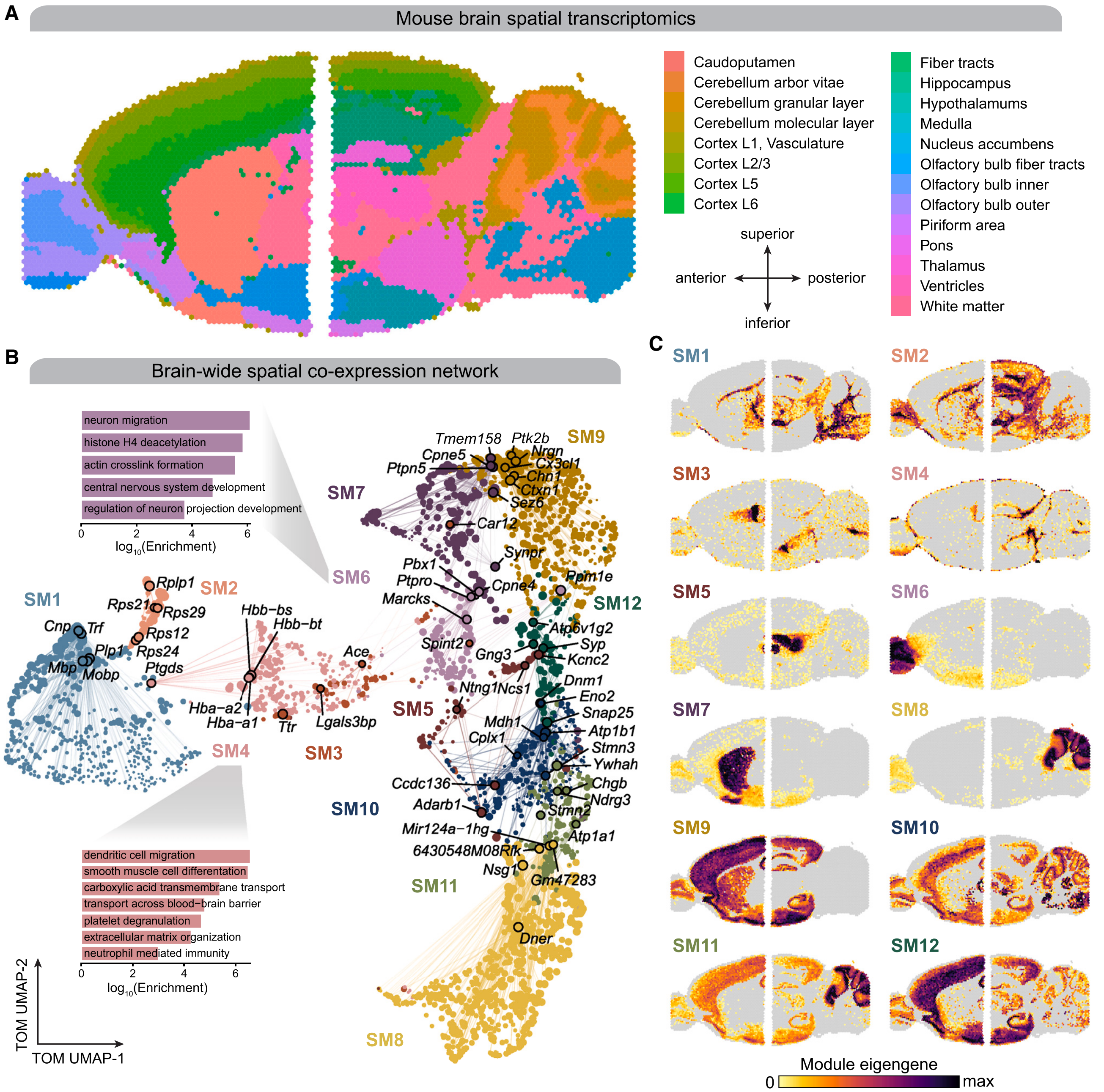

Figure 3. Spatial co-expression networks represent regional expression patterns in the mouse brain

(A) Visium spatial transcriptomics (ST) in anterior (left, 2,696 spots) and posterior (right, 3,353 spots) mouse brain sections, colored by Louvain clusters annotated by anatomical regions.

(B) UMAP plot of the mouse brain ST co-expression network. Each node represents a single gene, and edges represent co-expression links between genes and module hub genes. Point size is scaled by kME. Nodes are colored by co-expression module assignment. The top five hub genes per module are labeled. Network edges were downsampled for visual clarity.

(C) ST samples colored by MEs for the 12 spatial co-expression modules. Gray color indicates an ME value less than zero.

Isoform-level co-expression networks reveal cell fate decisions in the radial glia developmental lineage

Different isoforms of the same gene are often involved in distinct biological processes.[29] Conventional single-cell transcriptomics assays capture information at the gene level, thereby missing much of the biological diversity and regulatory mechanisms that occurs at the isoform level.[30] Emerging long-read sequencing approaches enable us to profile cellular transcriptomes at isoform

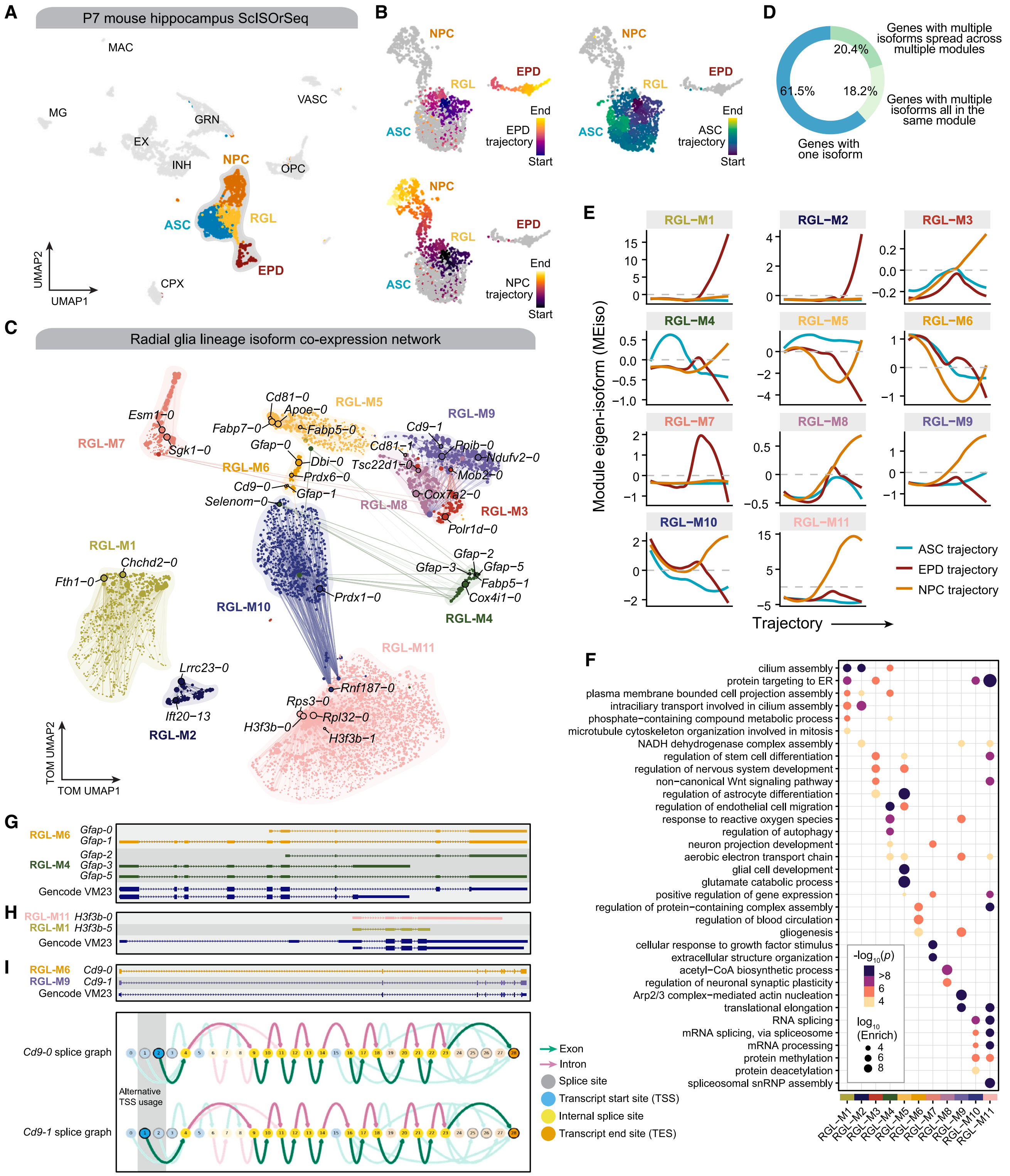

Figure 4. Isoform co-expression network analysis reveals fate-specific expression programs in the hippocampal radial glia lineage

(A) UMAP plot of cells from the mouse hippocampus ScISOrSeq dataset.[8] Major cell types are labeled and the cells used for co-expression network analysis are colored. This dataset contains expression information for 96,093 isoforms and 31,053 genes in 6,832 cells from one mouse brain sample. ASC, astrocytes; CPX,

(legend continued on next page)

resolution,[8,31–33] thus providing new opportunities to model the relationships between isoforms using co-expression network analysis.

We used hdWGCNA to perform isoform co-expression network analysis in radial glia lineage cells from the mouse hippocampus at postnatal day 7 (P7) profiled with single-cell isoform RNA sequencing (ScISOrSeq)[8] (Figure 4A; STAR Methods). This dataset contains isoform-level and gene-level expression data from 6,832 nuclei derived from a single mouse hippocampus sample. Radial glia, which share transcriptomic similarities with mature astrocytes, are progenitor cells that give rise to numerous distinct cell fates, including neuronal cells, astrocytes, oligodendrocytes, and ependymal cells.[34,35] To model this developmental process, we applied Monocle3[36] pseudotime to 2,190 radial glia lineage cells (Figure 4B). We identified three trajectories corresponding to distinct cell fates, termed the ependymal (EPD) trajectory, astrocyte (ASC) trajectory, and the neural intermediate progenitor cell (NPC) trajectory.

Isoform co-expression network analysis revealed 11 modules in the radial glia lineage (Figure 4C; Data S1). Of the genes retained for network analysis, 61.5% had a single isoform, 18.2% had multiple isoforms that were all assigned to the same module, and 20.4% had multiple isoforms spread across several modules (Figure 4D). Thus, these network modules capture information about the roles of different isoforms of the same gene in distinct biological processes. We inspected module eigenisoform (MEiso) patterns throughout the developmental lineage, thereby uncovering isoform modules critical for cell fate decisions (Figure 4E; Data S2 and S3). Increased expression of modules RGL-M1 and RGL-M2, which were enriched cilium assembly genes (Figure 4F), was associated with the transition from a radial glia to an ependymal cell state. A steady expression level of module RGL-M5 (glial development, astrocyte differentiation) was found in the transition from radial glia to astrocytes, while a decreased expression of RGL-M5 led to alternative fates. Four modules (RGL-M3, RGL-M8, RGL-M9, and RGL-M11) displayed an increase in expression in the neuronal trajectory, containing genes associated with cellular processes such as non-canonical *Wnt* signaling, neuronal synaptic plasticity, and RNA splicing (Figure 4F).

We inspected the isoforms of three selected genes that had hub isoforms in different co-expression modules: *Gfap*, *H3f3b*, and *Cd9* (Figures 4G–4I). *Gfap* encodes a key intermediate filament protein in astrocytes that is involved in astrocytic reactivity during central nervous system (CNS) injuries or neurodegeneration,[39] and we found that modules RGL-M4 and RGL-M6 contained hub isoforms of *Gfap* featuring alternative splicing, alternative transcription start site (TSS) usage, and alternative transcription end site (TES) usage (Figure 4G). Different isoforms of the histone H3.3 subunit gene *H3f3b* were hubs for modules RGL-M1 and RGL-M11, which were associated with ependymal and neuronal cell fates respectively, suggesting that alternative TES usage in *H3f3b* plays a role in regulating epigenetic factors in murine hippocampal development (Figure 4F). *Cd9* encodes a transmembrane protein and is a known glioblastoma biomarker,[40] and we found subtle differences in the TSS between hub isoforms in modules RGL-M6 and RGL-M9 that we show as a splicing summary graph[38] (Figure 4I), supporting functional changes mediated by small isoform differences.

Co-expression network analysis of inhibitory neurons in ASD

Co-expression networks can be interrogated to further understand the molecular phenotypes of complex polygenic diseases in primary human tissue samples. We applied hdWGCNA to 20,249 inhibitory neurons (INHs) from an snRNA-seq dataset of the human PFC in 22 ASD patients, 24 age-matched controls, and eight epilepsy patients[9] (Figures 5A and S10; Data S1). The INH network contained 14 modules, and we show hub genes that have a known association with ASD in the SFARI database on the co-expression UMAP (Figure 5B). The MEs showed that some modules were primarily confined to a single INH cluster (INH-M3, INH-M1) while others were spread across multiple neuronal groups (Figure 5C). Furthermore, DME analysis revealed significant differences between MEs in ASD and control samples for all modules except INH-M4 in at least one INH subpopulation (Figure 5D; Data S3; Wilcoxon rank-sum test Bonferroni-adjusted $p < 0.05$). However, by focusing on the DME results with an absolute average $\log_2$ (fold change) ≥ 0.5, we note that many of the largest differences were found in the *SST*$^+$ inhibitory neuron clusters. Furthermore, three co-expression modules (INH-M11, INH-M13, and INH-M3) were significantly enriched in ASD-associated genes from the SFARI database and the

choroid plexus epithelial cells; EPD, ependymal cells; EX, excitatory neurons; GRN, granule neurons; INH, inhibitory neurons; MAC, macrophages; NPC, neuronal intermediate progenitor cells; MG, microglia; OPC, oligodendrocyte progenitor cells; RGL, radial glia; VASC, vasculature cells.

(B) UMAP plot of the radial glia lineage, colored by Monocle 3[37] pseudotime assignment. Top left, ependymal (EPD) trajectory; top right, astrocyte (ASC) trajectory; bottom left, neuronal intermediate progenitor cell (NPC) trajectory.

(C) UMAP plot of the radial glia lineage isoform co-expression network. Each node represents a single isoform, and edges represent co-expression links between isoforms and module hub isoforms. Point size is scaled by kMEiso. Nodes are colored by co-expression module assignment. Network edges were downsampled for visual clarity.

(D) Donut chart showing the percentage of genes with one isoform, with multiple isoforms that are all assigned to the same module, and with multiple isoforms that are spread across more than one module.

(E) Module eigenisoforms (MEiso) as a function of pseudotime for each co-expression module. For each module, a separate locally estimated scatterplot smoothing (LOESS) regression line is shown for each developmental trajectory.

(F) Dot plot showing selected GO term enrichment results for each co-expression module.

(G) Gene models for selected isoforms of *Gfap*, colored by co-expression module assignment.

(H) Gene models for selected isoforms of *H3f3b*, colored by co-expression module assignment.

(I) Top: gene models for selected isoforms of *Cd9*, colored by co-expression module assignment. Bottom: Swan[38] graphical representation of *Cd9* alternative splicing isoforms. Splice sites and transcript start/end sites are represented as nodes; introns and exons are represented as connections between nodes. These two isoforms are distinguished by alternative TSS usage. Gene models from the GENCODE VM23 comprehensive transcript set are shown below transcripts in panels (G)–(I).

CellPress
OPEN ACCESS

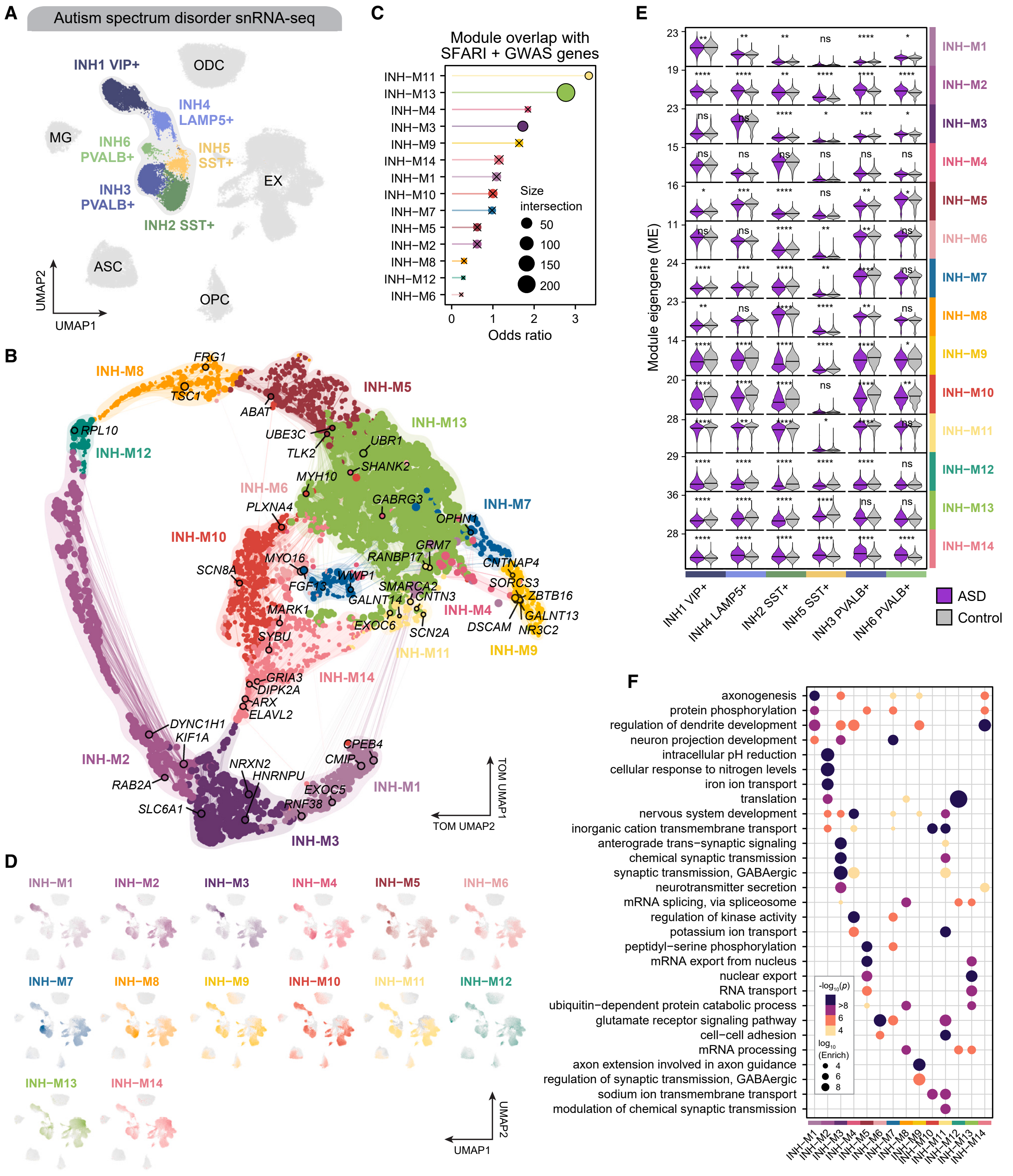

Figure 5. Co-expression network analysis of inhibitory neurons in Autism spectrum disorder

(A) UMAP plot of 121,451 nuclei from the cortex of 22 ASD donors, 24 controls, and eight epilepsy donors profiled with snRNA-seq. Inhibitory neuron subtypes are highlighted. ASC, astrocytes; EX, excitatory neurons; INH, inhibitory neurons; MG, microglia; ODC, oligodendrocytes; OPC, oligodendrocyte progenitor cells.

(legend continued on next page)

latest genome-wide association study (GWAS) of ASD[41] (Figure 5E), but we note that all of these modules contained several ASD-associated SFARI genes.

INH-M11 was enriched for genes associated with synaptic transmission, ion transport, glutamate receptor signaling, and nervous system development (Figure 5F; Data S2), and this module was downregulated in ASD for five of the six INH subtypes (Figure 5D). Similarly, INH-M13 was associated with RNA processing (Figure 5F) and was downregulated in ASD in all INH subtypes except *PVALB*+ neurons (Figure 5D). One of the INH-M13 hub genes is *CHD2*, whose *de novo* variants have been identified in individuals with ASD.[42,43] CHD2 is part of the CHD family of chromatin-modifying proteins and can alter gene expression by modification of chromatin structure. Similarly, rare loss-of-function mutations have been reported in the *SCN2A* gene, a hub gene of the INH-M11 module.[44] We also find enrichment of several ASD-associated genes such as *TSC1* (INH-M8), *SMARCA4* (INH-M8), *SHANK2* (INH-M4), and *CPEB4* (INH-M1), highlighting that these modules are functional and provide new insights into the role of inhibitory neurons in ASD. Finally, we tested for the preservation of these modules in 19,425 inhibitory neurons from an snRNA-seq dataset of the PFC from donors with major depressive disorder (MDD) and controls[45] (34 samples), and we found substantial evidence of preservation across all modules except INH-M1 (Figures S10C–S10E).

Consensus network analysis of microglia in AD

Microglia, the resident immune cells of the brain, are implicated in the pathology and genetic risk of several CNS diseases, including AD.[46–49] Transcriptomic and epigenomic studies in human tissue and AD mouse models have identified multiple cell states of microglia, representing a spectrum between homeostatic and disease-associated microglia (DAMs).[12,50,51] Our previous study defined a set of transcription factors, genes, and *cis*-regulatory elements involved in the shift between homeostatic and DAM cell states in human AD, identifying shared and distinct signatures compared with the DAM signature from 5xFAD mice.[12] Here we sought to expand on previous work by providing a systems-level analysis of gene expression throughout the spectrum of microglia cell states.

We modeled the cell-state continuum between homeostatic and DAM-like microglia by employing a pseudotime analysis of microglia from three human AD snRNA-seq datasets[10–12] (Figures 6A, 6B, and S11). Next, we performed consensus co-expression network analysis using microglia integrated from three human AD snRNA-seq datasets,[10–12] identifying four consensus modules (Figure 6C; Data S1). Consensus network analysis is an approach that performs network analysis separately for each dataset, followed by a procedure to retain structures common across the individual networks, and thus it is well suited for analyzing microglia co-expression from these different sources (STAR Methods).

Classical markers of homeostatic microglia, such as *CSF1R*, *CX3CR1*, and *P2RY12*, were members of MG-M2, while known DAM genes, including *APOE*, *TYROBP*, and *B2M*, were members of MG-M1. GO term enrichment analysis associated MG-M2 with homeostatic microglia functions such as cell migration, synapse organization, and response to colony-stimulating factor, contrasting disease-related processes enriched in MG-M1 including amyloid fibril formation, microglial activation, maintenance of blood-brain barrier, and cytokine production (Figure 6D; Data S2). Together, this suggests that MG-M1 comprises the gene network underlying DAM activation in AD, while MG-M2 represents the network of homeostatic microglia genes. The MEs for MG-M1 and MG-M2 display opposing patterns throughout the microglia pseudotime trajectory, contextualizing this trajectory as the transcriptional shift from homeostatic microglia (start) to a DAM-like cell state (end) (Figures 6E and 6F). Furthermore, DME analysis revealed significant changes in these modules between AD and control brains in evenly spaced windows throughout the microglia trajectory (Figure 6G; Wilcoxon rank-sum test Bonferroni-adjusted $p < 0.05$; Data S3). Co-expression networks behave as functional biological units; therefore, we reason that the hub genes and other members of MG-M1 represent candidates for an expanded set of human DAM genes including *ACTB*, *TPT1*, and *EEF1A1*.

Aside from modules MG-M1 and MG-M2, which contained well-known microglia gene signatures, we also identified modules MG-M3 and MG-M4 containing genes associated with key microglial processes such as axon guidance, phagocytosis, and myeloid cell differentiation (Figures 6C and 6D). *CD163*, a hub gene of MG-M4, is known to be involved in the breakdown of the blood-brain barrier.[53,54] The trajectory of MG-M4, containing *CD163* as a hub gene, was consistent with that of DAM-like module MG-M1, and was enriched for processes including phagocytosis, myeloid cell differentiation, and neutrophil activation (Figure 6D); therefore, it is possible that MG-M4 represents an alternative microglial activation module.[55] We performed single-cell polygenic risk enrichment for AD risk in the microglia trajectory,[46,52] and identified a significant increase throughout the trajectory, revealing an enrichment of AD genetic risk single-nucleotide polymorphisms (SNPs) in DAMs (Figure 6H; STAR Methods; Data S3). We show that expression of these modules was significantly correlated with AD genetic risk (Pearson correlation $p < 0.05$), with the strongest correlation in alternative activation module MG-M4 (Figure 6I).

(B) Gene co-expression network derived from inhibitory neurons, represented as a two-dimensional UMAP embedding of the TOM. Nodes represent genes, colored by module assignment. Module hub genes with prior evidence of ASD association from SFARI are labeled. Edges represent co-expression relationships between genes and module hub genes. Network edges were downsampled for visual clarity.

(C) Gene overlap analysis comparing ASD-associated genes from SFARI and INH co-expression modules, using Fisher's exact test. × indicates that the overlap was not significant (FDR > 0.05).

(D) snRNA-seq UMAP plots as in (A) colored by MEs for INH co-expression modules.

(E) Violin plots showing MEs in each INH cluster. Two-sided Wilcoxon test was used to compare ASD versus control samples. Nuclei from epilepsy donors were excluded in this comparison. Not significant (ns), $p > 0.05$; *$p \leq 0.05$; **$p \leq 0.01$; ***$p \leq 0.001$; ****$p \leq 0.0001$.

(F) Selected GO enrichment results for each co-expression module.

CellPress
OPEN ACCESS

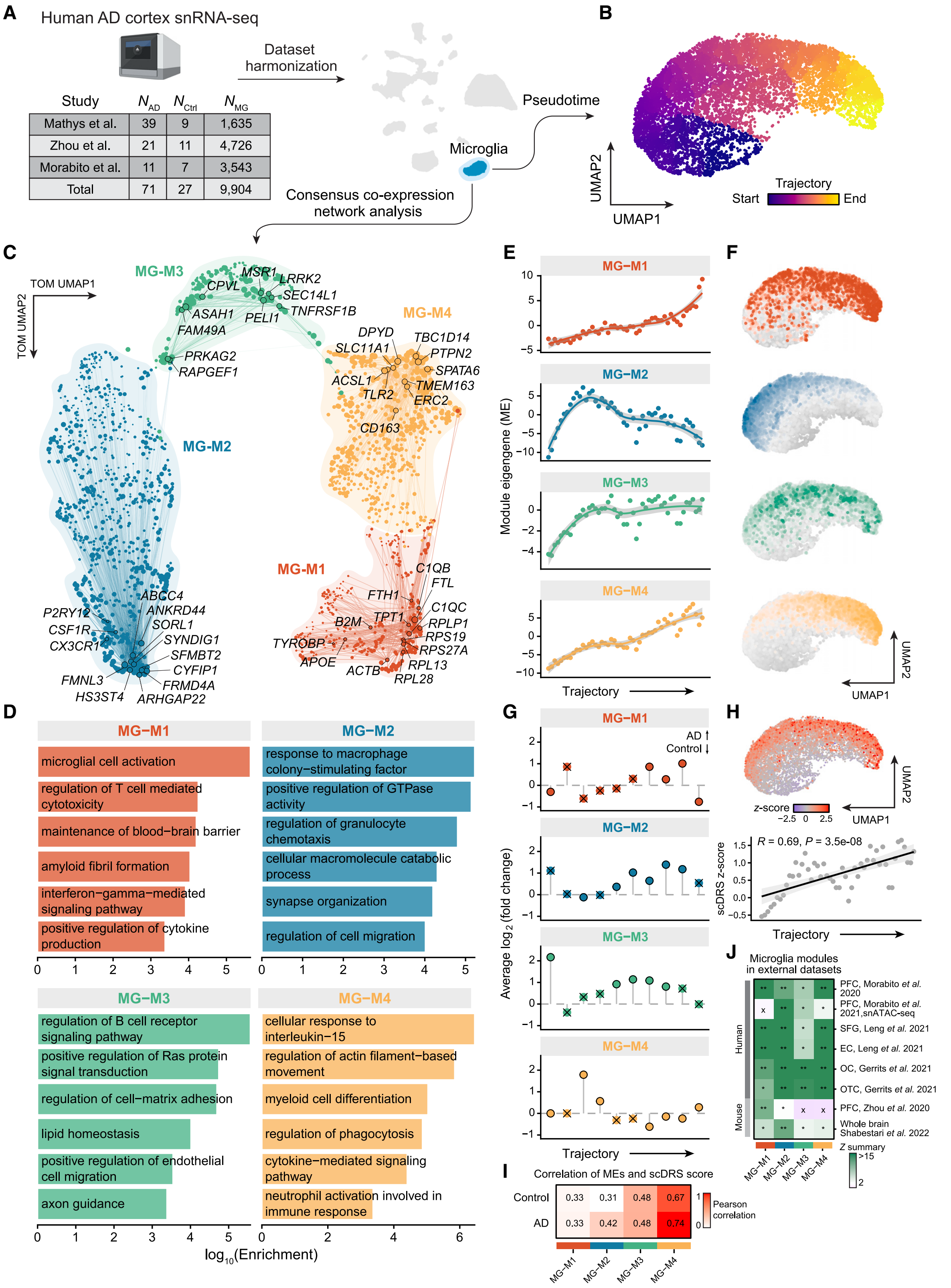

(legend on next page)

To ensure that these microglial modules were reproducible across other datasets and in mouse models of AD, we performed module preservation analysis[20] (STAR Methods; Figure 6J). We projected the microglial consensus modules into a dataset of the PFC in aged human samples,[56] the superior frontal gyrus (SFG) and entorhinal cortex (EC) in AD samples,[57] the occipital cortex (OC) and the occipitotemporal cortex (OTC) in human AD samples,[58] the PFC from 5xFAD mice,[11] and whole-brain samples from 5xFAD mice[28] (Figure 6H). Additionally, we projected these modules into an snATAC-seq dataset of the PFC in human AD,[12] using gene activity[59] as a proxy for gene expression from chromatin accessibility data. These module preservation tests showed the microglia consensus modules were broadly preserved and reproducible across brain regions and in mouse models of AD, providing further support that this network is relevant in AD biology and microglial activation.

Projecting network modules from bulk RNA-seq cohorts into relevant single-cell datasets

hdWGCNA allows for interrogating co-expression modules inferred from a given reference dataset in a query dataset. Modules can be projected across datasets by computing MEs in the query dataset, and preservation of the network structure can be assessed via statistical testing.[20] For example, modules can be projected between different species to link transcriptomic changes between mouse models and human disease patients, or modules can be projected across data modalities from single-cell to spatial transcriptomics to provide regional context to cellular niches.

To date, it remains cost-prohibitive for most researchers to perform high-dimensional -omics studies of large patient cohorts, but there are numerous large-scale disease-relevant bulk RNA-seq datasets containing thousands of samples from consortia such as the Encyclopedia of DNA Elements (ENCODE),[60] the Genotype-Tissue Expression (GTEx) project,[61] and The Cancer Genome Atlas (TCGA).[62] By projecting co-expression modules derived from bulk RNA-seq patient cohorts into single-cell datasets, we can layer disease-related information onto the single-cell dataset and attribute cell-state-specific expression patterns to the bulk RNA-seq data. We demonstrate projecting modules in this manner using co-expression modules from two bulk RNA-seq studies of AD[56,63] as the references and a human AD snRNA-seq dataset[12] (57,950 nuclei from AD 11 samples and seven control samples of the PFC) as the query. These studies both used AD samples and controls from the same patient cohorts (Religious Orders Study and Memory and Aging Project, Mayo Clinic, Mount Sinai School of Medicine),[64–66] but they took unique approaches for co-expression network analysis. The AMP-AD study from Wan et al.[63] performed network analysis separately from each brain region, while, in our previous study,[56] we performed consensus network analysis across the different brain regions. We projected these modules into a snRNA-seq dataset of AD and control samples from the PFC (Figure 7A), and we found distinct cell-type-specific expression patterns based on their MEs (Figures 7B and 7C). This analysis demonstrates hdWGCNA's ability to transfer co-expression information across datasets to uncover otherwise unseen biological insights.

DISCUSSION

Classical bioinformatic approaches for transcriptomics analysis such as differential gene expression are useful for finding individual genes that are altered in a particular disease or condition of interest, but they do not provide information about the broader context of these genes in specific pathways or regulatory regimes. For example, biological processes such as development or regeneration require coordination of distinct sets of genes in certain cell types with spatial specificity. Therefore, to understand these complex processes, we must look beyond individual genes. We developed hdWGCNA to provide a succinct methodology for investigating systems-level changes in the transcriptome in single-cell or ST datasets. We designed hdWGCNA to be highly modular, allowing for multi-scale analyses of different cellular or spatial hierarchies in a technology-agnostic manner.

In this study, we demonstrated that hdWGCNA is compatible with single-cell and ST datasets and can be easily adapted

Figure 6. Consensus network analysis of microglia in AD

(A) Left: table showing the number of samples and the number of microglia nuclei from published AD snRNA-seq datasets used for co-expression network analysis. Right: integrated UMAP plot of nuclei from three snRNA-seq datasets.

(B) UMAP plot of microglia, colored by Monocle 3[37] pseudotime assignment.

(C) UMAP plot of the microglia co-expression network. Each node represents a single gene, and edges represent co-expression links between genes and module hub genes. Point size is scaled by kME. Nodes are colored by co-expression module assignment. The top 10 hub genes per module are labeled, as well as additional genes of interest. Network edges were downsampled for visual clarity.

(D) Selected Gene Ontology (GO) terms enriched in co-expression modules. Bar plots show the log-scaled enrichment of each term.

(E) MEs as a function of pseudotime; points are averaged MEs in 50 pseudotime bins of equal size. Line represents LOESS regression with a 95% confidence interval.

(F) Microglia UMAP colored by ME.

(G) Differential module eigengene (DME) results in 10 pseudotime bins of equal size. For each pseudotime bin, we performed DME analysis between cells from AD (positive fold change) and control samples. × symbol indicates that the test did not reach significance (Wilcoxon rank-sum test Bonferroni-adjusted p value > 0.05).

(H) Top: microglia UMAP colored by AD single-cell disease relevance score (scDRS)[52] Z score. Bottom: scDRS Z score as a function of pseudotime, points are averaged scDRS Z scores in 50 pseudotime bins of equal size. Line represents linear regression with a 95% confidence interval.

(I) Heatmap of Pearson correlations of MEs and scDRS Z scores, split by cells from AD and control samples.

(J) Abbreviations denote the following brain regions: SFG, superior frontal gyrus; EC, entorhinal cortex; OC, occipital cortex; OTC, occipitotemporal cortex. **Highly preserved ($Z \geq 10$); *moderately preserved ($10 > Z \geq 5$); x, not preserved ($Z < 5$).

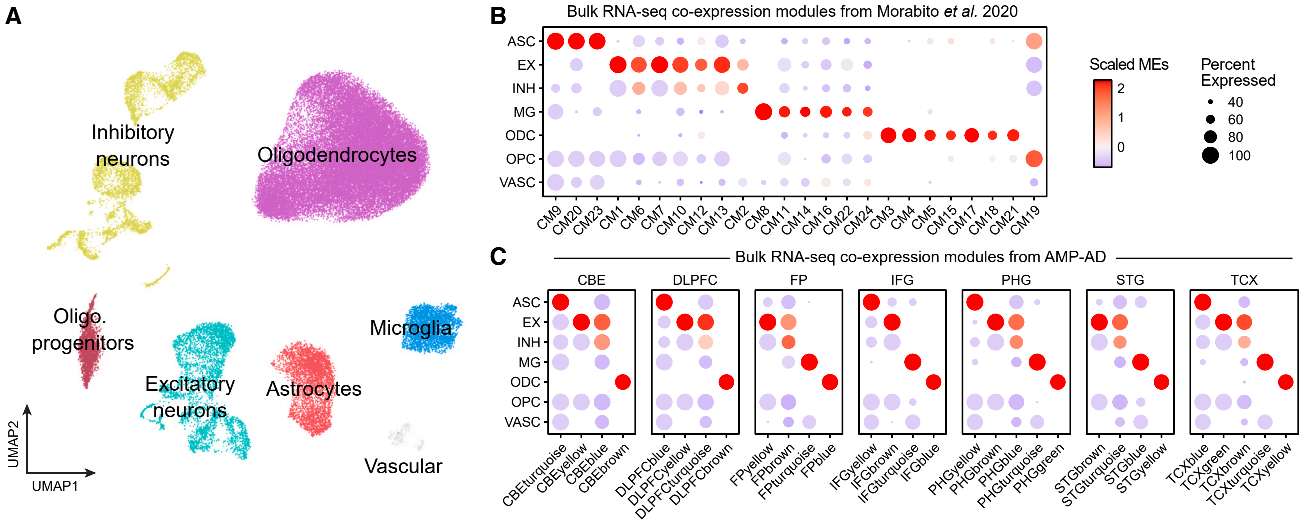

Figure 7. Projecting bulk RNA-seq co-expression modules into a single-cell dataset

(A) UMAP plot of 57,950 nuclei from an snRNA-seq dataset of the human PFC from AD (N = 11) and control (N = 7) PFC samples.[12] Cells are colored by major cell type assignment.

(B) Multi-region consensus co-expression modules from Morabito et al.[56] bulk RNA-seq analysis projected into the snRNA-seq dataset as in (A).

(C) Co-expression modules from the AMP-AD bulk RNA-seq dataset[63] projected into the snRNA-seq dataset as in (A). CBE, cerebellum; DLPFC, dorsolateral PFC; FP, frontal pole; IFG, inferior frontal gyrus; PHG, parahippocampal gyrus; STG, superior temporal gyrus; TCX, temporal cortex; ASC, astrocytes; EX, excitatory neurons; INH, inhibitory neurons; MG, microglia; ODC, oligodendrocytes; OPC, oligodendrocyte progenitor cells; VASC, vascular cells.

for novel transcriptomics approaches such as ScISOrSeq. Co-expression networks have been successful for analyzing bulk proteomics datasets in human disease samples,[67,68] and we expect that hdWGCNA could be swiftly adapted for single-cell and spatial proteomics datasets as the technology matures and becomes more widely available.[69] hdWGCNA includes built-in functions to leverage external biological knowledge sources to provide insight for co-expression networks, for example by comparing gene modules with functional gene sets such as disease-associated genes from GWAS expression quantitative trait loci (eQTLs), or transcription factor target genes. Unlike other network analysis pipelines such as single-cell regulatory network inference and clustering (SCENIC)[70] or CellChat,[71] hdWGCNA is a purely unsupervised approach and does not require prior knowledge or databases in the inference procedure. The co-expression information computed by hdWGCNA can be easily retrieved from the Seurat object to facilitate custom downstream analyses beyond the hdWGCNA package. hdWGCNA allows for comparisons between experimental groups via DME testing and module preservation analysis, which allowed us to identity inhibitory neuron modules that were dysregulated in ASD and enriched for ASD genetic risk genes, and microglial modules that were dysregulated in AD and enriched for DAM genes. Our network analyses of the ASD and AD datasets shows that hdWGCNA is capable of uncovering expanded disease-relevant gene sets via the interaction partners of known disease-associated genes such as the ASD SFARI genes or the AD DAM genes. We showed that the co-expression networks inferred by hdWGCNA were highly reproducible in unseen datasets, indicating that this is a robust methodology that reflects the underlying biology of the system of interest rather than picking up on technical artifacts. Further, hdWGCNA sheds new light on previously identified co-expression networks and gene modules by allowing modules to be projected from a reference dataset to a query dataset. The hdWGCNA R package directly extends the familiar Seurat pipeline and the SeuratObject data structure, enabling researchers to rapidly incorporate network analysis into their own workflows, going beyond cell clustering and differential gene expression analysis toward systems-level insights.

Limitations of the study

Transcriptomic measurements of single cells are generally noisy, imposing challenges and limitations in the analysis of these datasets. Technical noise may arise from dropout events or from various steps in the experimental protocols, potentially making downstream data analysis and interpretation more difficult. hdWGCNA explicitly tries to handle the issues of technical dropouts and data sparsity by constructing networks in metacell or metaspot transcriptomic profiles rather than directly using the single-cell data. Furthermore, we show that module preservation statistical testing can assess the reproducibility of a co-expression network in external validation datasets, giving additional confidence in the results from hdWGCNA.

STAR★METHODS

Detailed methods are provided in the online version of this paper and include the following:

- KEY RESOURCES TABLE

- RESOURCE AVAILABILITY
  - Lead contact
  - Materials availability
  - Data and code availability
- METHOD DETAILS
  - Bootstrapped aggregation of single cell transcriptomes to form metacells
  - Aggregation of neighboring spatial transcriptomic spots to form metaspots
  - Computing co-expression networks
  - Computing module eigengenes
  - Projecting co-expression modules in unseen data
  - Implementation of the hdWGCNA R package
- QUANTIFICATION AND STATISTICAL ANALYSIS
  - Reprocessing published datasets
  - Iterative network analysis of major cell types in the human cortex
  - Comparison of hdWGCNA with alternative metacell approaches
  - Application of hdWGCNA to a one million cell scRNA-seq dataset
  - Runtime and memory usage of hdWGCNA
  - Evaluating performance of hdWGCNA co-expression networks
  - Spatial co-expression network analysis in the mouse brain
  - Isoform co-expression network analysis in the mouse hippocampus
  - Co-expression analysis network of inhibitory neurons in autism spectrum disorder
  - Consensus co-expression network analysis of microglia in Alzheimer's disease
  - Analysis of bulk RNA-seq co-expression modules in single-cell data

SUPPLEMENTAL INFORMATION

Supplemental information can be found online at https://doi.org/10.1016/j.crmeth.2023.100498.

ACKNOWLEDGMENTS

Funding for this work was provided by National Institutes on Aging, Neurological Disorders and Stroke, and Drug Abuse grants 1RF1AG071683, P01NS084974- 06A1, 1U01DA053826, U54 AG054349-06 (MODEL-AD), and 3U19AG068054-02S, Adelson Medical Research Foundation funds to V.S. This work utilized the infrastructure for high-performance and high-throughput computing, research data storage and analysis, and scientific software tool integration built, operated, and updated by the Research Cyberinfrastructure Center (RCIC) at the University of California, Irvine (UCI). We thank Anoushka Joglekar for providing the ScISOrSeq dataset.

AUTHOR CONTRIBUTIONS

S.M. and V.S. conceptualized this study. The manuscript was written by S.M., F.R., and E.M. with assistance and approval from all authors. S.M. developed the hdWGCNA R package. S.M. and F.R. designed the structure of the hdWGCNA R package. S.M. collected, processed, and performed network analysis on publicly available sequencing datasets. F.R. performed bioinformatics analysis of the ScISOrSeq dataset. N.R. performed polygenic risk analysis of the integrated microglia snRNA-seq dataset.

DECLARATION OF INTERESTS

The authors declare no competing interests.

Received: October 5, 2022
Revised: February 13, 2023
Accepted: May 16, 2023
Published: June 12, 2023

REFERENCES

1. Langfelder, P., and Horvath, S. (2008). WGCNA: an R package for weighted correlation network analysis. BMC Bioinf. *9*, 559. https://doi.org/10.1186/1471-2105-9-559.
2. Yip, A.M., and Horvath, S. (2007). Gene network interconnectedness and the generalized topological overlap measure. BMC Bioinf. *8*, 22. https://doi.org/10.1186/1471-2105-8-22.
3. Langfelder, P., Zhang, B., and Horvath, S. (2008). Defining clusters from a hierarchical cluster tree: the Dynamic Tree Cut package for R. Bioinformatics *24*, 719–720. https://doi.org/10.1093/bioinformatics/btm563.
4. Dong, J., and Horvath, S. (2007). Understanding network concepts in modules. BMC Syst. Biol. *1*, 24. https://doi.org/10.1186/1752-0509-1-24.
5. Butler, A., Hoffman, P., Smibert, P., Papalexi, E., and Satija, R. (2018). Integrating single-cell transcriptomic data across different conditions, technologies, and species. Nat. Biotechnol. *36*, 411–420. https://doi.org/10.1038/nbt.4096.
6. Stuart, T., Butler, A., Hoffman, P., Hafemeister, C., Papalexi, E., Mauck, W.M., Hao, Y., Stoeckius, M., Smibert, P., and Satija, R. (2019). Comprehensive integration of single-cell data. Cell *177*, 1888–1902.e21. https://doi.org/10.1016/j.cell.2019.05.031.
7. Hao, Y., Hao, S., Andersen-Nissen, E., Mauck, W.M., Zheng, S., Butler, A., Lee, M.J., Wilk, A.J., Darby, C., et al. (2021). Integrated analysis of multimodal single-cell data. Cell *184*, 3573–3587.e29. https://doi.org/10.1016/j.cell.2021.04.048.
8. Joglekar, A., Prjibelski, A., Mahfouz, A., Collier, P., Lin, S., Schlusche, A.K., Marrocco, J., Williams, S.R., Haase, B., Hayes, A., et al. (2021). A spatially resolved brain region- and cell type-specific isoform atlas of the postnatal mouse brain. Nat. Commun. *12*, 463. https://doi.org/10.1038/s41467-020-20343-5.
9. Velmeshev, D., Schirmer, L., Jung, D., Haeussler, M., Perez, Y., Mayer, S., Bhaduri, A., Goyal, N., Rowitch, D.H., and Kriegstein, A.R. (2019). Single-cell genomics identifies cell type–specific molecular changes in autism. Science *364*, 685–689. https://doi.org/10.1126/science.aav8130.
10. Mathys, H., Davila-Velderrain, J., Peng, Z., Gao, F., Mohammadi, S., Young, J.Z., Menon, M., He, L., Abdurrob, F., Jiang, X., et al. (2019). Single-cell transcriptomic analysis of Alzheimer's disease. Nature *570*, 332–337. https://doi.org/10.1038/s41586-019-1195-2.
11. Zhou, Y., Song, W.M., Andhey, P.S., Swain, A., Levy, T., Miller, K.R., Poliani, P.L., Cominelli, M., Grover, S., Gilfillan, S., et al. (2020). Human and mouse single-nucleus transcriptomics reveal TREM2-dependent and TREM2-independent cellular responses in Alzheimer's disease. Nat. Med. *26*, 131–142. https://doi.org/10.1038/s41591-019-0695-9.
12. Morabito, S., Miyoshi, E., Michael, N., Shahin, S., Martini, A.C., Head, E., Silva, J., Leavy, K., Perez-Rosendahl, M., and Swarup, V. (2021). Single-nucleus chromatin accessibility and transcriptomic characterization of Alzheimer's disease. Nat. Genet. *53*, 1143–1155. https://doi.org/10.1038/s41588-021-00894-z.
13. Baran, Y., Bercovich, A., Sebe-Pedros, A., Lubling, Y., Giladi, A., Chomsky, E., Meir, Z., Hoichman, M., Lifshitz, A., and Tanay, A. (2019). MetaCell: analysis of single-cell RNA-seq data using K-nn graph partitions. Genome Biol. *20*, 206. https://doi.org/10.1186/s13059-019-1812-2.
14. Ben-Kiki, O., Bercovich, A., Lifshitz, A., and Tanay, A. (2022). Metacell-2: a divide-and-conquer metacell algorithm for scalable scRNA-seq analysis. Genome Biol. *23*, 100. https://doi.org/10.1186/s13059-022-02667-1.

15. Pliner, H.A., Packer, J.S., McFaline-Figueroa, J.L., Cusanovich, D.A., Daza, R.M., Aghamirzaie, D., Srivatsan, S., Qiu, X., Jackson, D., Minkina, A., et al. (2018). Cicero predicts cis-regulatory DNA interactions from single-cell chromatin accessibility data. Mol. Cell *71*, 858–871.e8. https://doi.org/10.1016/j.molcel.2018.06.044.

16. Persad, S., Choo, Z.-N., Dien, C., Sohail, N., Masilionis, I., Chaligné, R., Nawy, T., Brown, C.C., Sharma, R., Pe'er, I., et al. (2023). SEACells infers transcriptional and epigenomic cellular states from single-cell genomics data. Nat. Biotechnol. https://doi.org/10.1038/s41587-023-01716-9.

17. Squair, J.W., Gautier, M., Kathe, C., Anderson, M.A., James, N.D., Hutson, T.H., Hudelle, R., Qaiser, T., Matson, K.J.E., Barraud, Q., et al. (2021). Confronting false discoveries in single-cell differential expression. Nat. Commun. *12*, 5692. https://doi.org/10.1038/s41467-021-25960-2.

18. Andreatta, M., and Carmona, S.J. (2021). UCell: robust and scalable single-cell gene signature scoring. Comput. Struct. Biotechnol. J. *19*, 3796–3798. https://doi.org/10.1016/j.csbj.2021.06.043.

19. McInnes, L., Healy, J., and Melville, J. (2018). UMAP: uniform manifold approximation and projection for dimension reduction. Preprint at arXiv. https://doi.org/10.48550/arXiv.1802.03426.

20. Langfelder, P., Luo, R., Oldham, M.C., and Horvath, S. (2011). Is my network module preserved and reproducible? PLoS Comput. Biol. *7*, e1001057. https://doi.org/10.1371/journal.pcbi.1001057.

21. Baglama, J., and Reichel, L. (2005). Augmented implicitly restarted Lanczos bidiagonalization methods. SIAM J. Sci. Comput. *27*, 19–42. https://doi.org/10.1137/04060593x.

22. Korsunsky, I., Millard, N., Fan, J., Slowikowski, K., Zhang, F., Wei, K., Baglaenko, Y., Brenner, M., Loh, P.-r., and Raychaudhuri, S. (2019). Fast, sensitive and accurate integration of single-cell data with Harmony. Nat. Methods *16*, 1289–1296. https://doi.org/10.1038/s41592-019-0619-0.

23. Ballouz, S., Weber, M., Pavlidis, P., and Gillis, J. (2017). EGAD: ultra-fast functional analysis of gene networks. Bioinformatics *33*, 612–614. https://doi.org/10.1093/bioinformatics/btw695.

24. Skinnider, M.A., Squair, J.W., and Foster, L.J. (2019). Evaluating measures of association for single-cell transcriptomics. Nat. Methods *16*, 381–386. https://doi.org/10.1038/s41592-019-0372-4.

25. Chen, T., and Guestrin, C. (2016). XGBoost: A scalable tree boosting system. In Proceedings of the 22nd ACM SIGKDD International Conference on Knowledge Discovery and Data Mining (KDD '16), pp. 785–794. https://doi.org/10.1145/2939672.2939785.

26. Moses, L., and Pachter, L. (2021). Museum of Spatial Transcriptomics. Preprint at bioRxiv. https://doi.org/10.1101/2021.05.11.443152.

27. Moffitt, J.R., Lundberg, E., and Heyn, H. (2022). The emerging landscape of spatial profiling technologies. Nat. Rev. Genet. *23*, 741–759. https://doi.org/10.1038/s41576-022-00515-3.

28. Kiani Shabestari, S., Morabito, S., Danhash, E.P., McQuade, A., Sanchez, J.R., Miyoshi, E., Chadarevian, J.P., Claes, C., Coburn, M.A., Hasselmann, J., et al. (2022). Absence of microglia promotes diverse pathologies and early lethality in Alzheimer's disease mice. Cell Rep. *39*, 110961. https://doi.org/10.1016/j.celrep.2022.110961.

29. Wright, C.J., Smith, C.W.J., and Jiggins, C.D. (2022). Alternative splicing as a source of phenotypic diversity. Nat. Rev. Genet. *23*, 697–710. https://doi.org/10.1038/s41576-022-00514-4.

30. Amarasinghe, S.L., Su, S., Dong, X., Zappia, L., Ritchie, M.E., and Gouil, Q. (2020). Opportunities and challenges in long-read sequencing data analysis. Genome Biol. *21*, 30. https://doi.org/10.1186/s13059-020-1935-5.

31. Rebboah, E., Reese, F., Williams, K., Balderrama-Gutierrez, G., McGill, C., Trout, D., Rodriguez, I., Liang, H., Wold, B.J., and Mortazavi, A. (2021). Mapping and modeling the genomic basis of differential RNA isoform expression at single-cell resolution with LR-Split-seq. Genome Biol. *22*, 286. https://doi.org/10.1186/s13059-021-02505-w.

32. Palmer, C.R., Liu, C.S., Romanow, W.J., Lee, M.-H., and Chun, J. (2021). Altered cell and RNA isoform diversity in aging Down syndrome brains. Proc. Natl. Acad. Sci. USA *118*, e2114326118. https://doi.org/10.1073/pnas.2114326118.

33. Hardwick, S.A., Hu, W., Joglekar, A., Fan, L., Collier, P.G., Foord, C., Balacco, J., Lanjewar, S., Sampson, M.M., Koopmans, F., et al. (2022). Single-nuclei isoform RNA sequencing unlocks barcoded exon connectivity in frozen brain tissue. Nat. Biotechnol. *40*, 1082–1092. https://doi.org/10.1038/s41587-022-01231-3.

34. Pollen, A.A., Nowakowski, T.J., Chen, J., Retallack, H., Sandoval-Espinosa, C., Nicholas, C.R., Shuga, J., Liu, S.J., Oldham, M.C., Diaz, A., et al. (2015). Molecular identity of human outer radial glia during cortical development. Cell *163*, 55–67. https://doi.org/10.1016/j.cell.2015.09.004.

35. Eze, U.C., Bhaduri, A., Haeussler, M., Nowakowski, T.J., and Kriegstein, A.R. (2021). Single-cell atlas of early human brain development highlights heterogeneity of human neuroepithelial cells and early radial glia. Nat. Neurosci. *24*, 584–594. https://doi.org/10.1038/s41593-020-00794-1.

36. Cao, J., Spielmann, M., Qiu, X., Huang, X., Ibrahim, D.M., Hill, A.J., Zhang, F., Mundlos, S., Christiansen, L., Steemers, F.J., et al. (2019). The single-cell transcriptional landscape of mammalian organogenesis. Nature *566*, 496–502. https://doi.org/10.1038/s41586-019-0969-x.

37. Trapnell, C., Cacchiarelli, D., Grimsby, J., Pokharel, P., Li, S., Morse, M., Lennon, N.J., Livak, K.J., Mikkelsen, T.S., and Rinn, J.L. (2014). The dynamics and regulators of cell fate decisions are revealed by pseudotemporal ordering of single cells. Nat. Biotechnol. *32*, 381–386. https://doi.org/10.1038/nbt.2859.

38. Reese, F., and Mortazavi, A. (2021). Swan: a library for the analysis and visualization of long-read transcriptomes. Bioinformatics *37*, 1322–1323. https://doi.org/10.1093/bioinformatics/btaa836.

39. Hol, E.M., and Pekny, M. (2015). Glial fibrillary acidic protein (GFAP) and the astrocyte intermediate filament system in diseases of the central nervous system. Curr. Opin. Cell Biol. *32*, 121–130. https://doi.org/10.1016/j.ceb.2015.02.004.

40. Podergajs, N., Motaln, H., Rajčević, U., Verbovšek, U., Koršič, M., Obad, N., Espedal, H., Vittori, M., Herold-Mende, C., Miletic, H., et al. (2016). Transmembrane protein CD9 is glioblastoma biomarker, relevant for maintenance of glioblastoma stem cells. Oncotarget *7*, 593–609. https://doi.org/10.18632/oncotarget.5477.

41. Grove, J., Ripke, S., Als, T.D., Mattheisen, M., Walters, R.K., Won, H., Pallesen, J., Agerbo, E., Andreassen, O.A., Anney, R., Awashti, S., et al. (2019). Identification of common genetic risk variants for autism spectrum disorder. Nat. Genet. *51*, 431–444. https://doi.org/10.1038/s41588-019-0344-8.

42. Carvill, G.L., Heavin, S.B., Yendle, S.C., McMahon, J.M., O'Roak, B.J., Cook, J., Khan, A., Dorschner, M.O., Weaver, M., Calvert, S., et al. (2013). Targeted resequencing in epileptic encephalopathies identifies de novo mutations in CHD2 and SYNGAP1. Nat. Genet. *45*, 825–830. https://doi.org/10.1038/ng.2646.

43. Neale, B.M., Kou, Y., Liu, L., Ma'ayan, A., Samocha, K.E., Sabo, A., Lin, C.-F., Stevens, C., Wang, L.-S., Makarov, V., et al. (2012). Patterns and rates of exonic de novo mutations in autism spectrum disorders. Nature *485*, 242–245. https://doi.org/10.1038/nature11011.

44. Carroll, L.S., Woolf, R., Ibrahim, Y., Williams, H.J., Dwyer, S., Walters, J., Kirov, G., O'Donovan, M.C., and Owen, M.J. (2016). Mutation screening of SCN2A in schizophrenia and identification of a novel loss-of-function mutation. Psychiatr. Genet. *26*, 60–65. https://doi.org/10.1097/ypg.0000000000000110.

45. Nagy, C., Maitra, M., Tanti, A., Suderman, M., Théroux, J.F., Davoli, M.A., Perlman, K., Yerko, V., Wang, Y.C., Tripathy, S.J., et al. (2020). Single-nucleus transcriptomics of the prefrontal cortex in major depressive disorder implicates oligodendrocyte precursor cells and excitatory neurons. Nat. Neurosci. *23*, 771–781. https://doi.org/10.1038/s41593-020-0621-y.

46. Jansen, I.E., Savage, J.E., Watanabe, K., Bryois, J., Williams, D.M., Steinberg, S., Sealock, J., Karlsson, I.K., Hägg, S., Athanasiu, L., et al. (2019). Genome-wide meta-analysis identifies new loci and functional pathways

influencing Alzheimer's disease risk. Nat. Genet. *51*, 404–413. https://doi.org/10.1038/s41588-018-0311-9.

47. Kunkle, B.W., Grenier-Boley, B., Sims, R., Bis, J.C., Damotte, V., Naj, A.C., Boland, A., Vronskaya, M., van der Lee, S.J., Amlie-Wolf, A., et al. (2019). Genetic meta-analysis of diagnosed Alzheimer's disease identifies new risk loci and implicates A, tau, immunity and lipid processing. Nat. Genet. *51*, 414–430. https://doi.org/10.1038/s41588-019-0358-2.

48. Schwartzentruber, J., Cooper, S., Liu, J.Z., Barrio-Hernandez, I., Bello, E., Kumasaka, N., Young, A.M.H., Franklin, R.J.M., Johnson, T., Estrada, K., et al. (2021). Genome-wide meta-analysis, fine-mapping and integrative prioritization implicate new Alzheimer's disease risk genes. Nat. Genet. *53*, 392–402. https://doi.org/10.1038/s41588-020-00776-w.

49. Bellenguez, C., Küçükali, F., Jansen, I.E., Kleineidam, L., Moreno-Grau, S., Amin, N., Naj, A.C., Campos-Martin, R., Grenier-Boley, B., Andrade, V., et al. (2022). New insights into the genetic etiology of Alzheimer's disease and related dementias. Nat. Genet. *54*, 412–436. https://doi.org/10.1038/s41588-022-01024-z.

50. Keren-Shaul, H., Spinrad, A., Weiner, A., Matcovitch-Natan, O., Dvir-Szternfeld, R., Ulland, T.K., David, E., Baruch, K., Lara-Astaiso, D., Toth, B., et al. (2017). A unique microglia type associated with restricting development of Alzheimer's disease. Cell *169*, 1276–1290.e17. https://doi.org/10.1016/j.cell.2017.05.018.

51. Sala Frigerio, C., Wolfs, L., Fattorelli, N., Thrupp, N., Voytyuk, I., Schmidt, I., Mancuso, R., Chen, W.-T., Woodbury, M.E., Srivastava, G., et al. (2019). The major risk factors for Alzheimer's disease: age, sex, and genes modulate the microglia response to A plaques. Cell Rep. *27*, 1293–1306.e6. https://doi.org/10.1016/j.celrep.2019.03.099.

52. Zhang, M.J., Hou, K., Dey, K.K., Sakaue, S., Jagadeesh, K.A., Weinand, K., Taychameekiatchai, A., Rao, P., Pisco, A.O., Zou, J., et al. (2022). Polygenic enrichment distinguishes disease associations of individual cells in single-cell RNA-seq data. Nat. Genet. *54*, 1572–1580. https://doi.org/10.1038/s41588-022-01167-z.

53. Borda, J.T., Alvarez, X., Mohan, M., Hasegawa, A., Bernardino, A., Jean, S., Aye, P., and Lackner, A.A. (2008). CD163, a marker of perivascular macrophages, is up-regulated by microglia in simian immunodeficiency virus encephalitis after haptoglobin-hemoglobin complex stimulation and is suggestive of breakdown of the blood-brain barrier. Am. J. Pathol. *172*, 725–737. https://doi.org/10.2353/ajpath.2008.070848.

54. Pey, P., Pearce, R.K.B., Kalaitzakis, M.E., Griffin, W.S.T., and Gentleman, S.M. (2014). Phenotypic profile of alternative activation marker CD163 is different in Alzheimer's and Parkinson's disease. Acta Neuropathol. Commun. *2*, 21. https://doi.org/10.1186/2051-5960-2-21.

55. Nguyen, A.T., Wang, K., Hu, G., Wang, X., Miao, Z., Azevedo, J.A., Suh, E., Van Deerlin, V.M., Choi, D., Roeder, K., et al. (2020). APOE and TREM2 regulate amyloid-responsive microglia in Alzheimer's disease. Acta Neuropathol. *140*, 477–493. https://doi.org/10.1007/s00401-020-02200-3.

56. Morabito, S., Miyoshi, E., Michael, N., and Swarup, V. (2020). Integrative genomics approach identifies conserved transcriptomic networks in Alzheimer's disease. Hum. Mol. Genet. *29*, 2899–2919. https://doi.org/10.1093/hmg/ddaa182.

57. Leng, K., Li, E., Eser, R., Piergies, A., Sit, R., Tan, M., Neff, N., Li, S.H., Rodriguez, R.D., Suemoto, C.K., et al. (2021). Molecular characterization of selectively vulnerable neurons in Alzheimer's disease. Nat. Neurosci. *24*, 276–287. https://doi.org/10.1038/s41593-020-00764-7.

58. Gerrits, E., Brouwer, N., Kooistra, S.M., Woodbury, M.E., Vermeiren, Y., Lambourne, M., Mulder, J., Kummer, M., Möller, T., Biber, K., et al. (2021). Distinct amyloid- and tau-associated microglia profiles in Alzheimer's disease. Acta Neuropathol. *141*, 681–696. https://doi.org/10.1007/s00401-021-02263-w.

59. Stuart, T., Srivastava, A., Madad, S., Lareau, C.A., and Satija, R. (2021). Single-cell chromatin state analysis with Signac. Nat. Methods *18*, 1333–1341. https://doi.org/10.1038/s41592-021-01282-5.

60. ENCODE Project Consortium; Moore, J.E., Purcaro, M.J., Pratt, H.E., Epstein, C.B., Shoresh, N., Adrian, J., Kawli, T., Davis, C.A., Dobin, A., et al. (2020). Expanded encyclopaedias of DNA elements in the human and mouse genomes. Nature *583*, 699–710. https://doi.org/10.1038/s41586-020-2493-4.

61. GTEx Consortium (2020). The GTEx Consortium atlas of genetic regulatory effects across human tissues. Science *369*, 1318–1330. https://doi.org/10.1126/science.aaz1776.

62. Hoadley, K.A., Yau, C., Hinoue, T., Wolf, D.M., Lazar, A.J., Drill, E., Shen, R., Taylor, A.M., Cherniack, A.D., Thorsson, V., et al. (2018). Cell-of-Origin patterns dominate the molecular classification of 10,000 tumors from 33 types of cancer. Cell *173*, 291–304.e6. https://doi.org/10.1016/j.cell.2018.03.022.

63. Wan, Y.-W., Al-Ouran, R., Mangleburg, C.G., Perumal, T.M., Lee, T.V., Allison, K., Swarup, V., Funk, C.C., Gaiteri, C., Allen, M., Wang, M., et al. (2020). Meta-analysis of the Alzheimer's disease human brain transcriptome and functional dissection in mouse models. Cell Rep. *32*, 107908. https://doi.org/10.1016/j.celrep.2020.107908.

64. Mostafavi, S., Gaiteri, C., Sullivan, S.E., White, C.C., Tasaki, S., Xu, J., Taga, M., Klein, H.-U., Patrick, E., Komashko, V., et al. (2018). A molecular network of the aging human brain provides insights into the pathology and cognitive decline of Alzheimer's disease. Nat. Neurosci. *21*, 811–819. https://doi.org/10.1038/s41593-018-0154-9.

65. Allen, M., Carrasquillo, M.M., Funk, C., Heavner, B.D., Zou, F., Younkin, C.S., Burgess, J.D., Chai, H.-S., Crook, J., Eddy, J.A., et al. (2016). Human whole genome genotype and transcriptome data for Alzheimer's and other neurodegenerative diseases. Sci. Data *3*, 160089. https://doi.org/10.1038/sdata.2016.89.

66. Wang, M., Beckmann, N.D., Roussos, P., Wang, E., Zhou, X., Wang, Q., Ming, C., Neff, R., Ma, W., Fullard, J.F., et al. (2018). The Mount Sinai cohort of large-scale genomic, transcriptomic and proteomic data in Alzheimer's disease. Sci. Data *5*, 180185. https://doi.org/10.1038/sdata.2018.185.

67. Swarup, V., Chang, T.S., Duong, D.M., Dammer, E.B., Dai, J., Lah, J.J., Johnson, E.C.B., Seyfried, N.T., Levey, A.I., and Geschwind, D.H. (2020). Identification of conserved proteomic networks in neurodegenerative dementia. Cell Rep. *31*, 107807. https://doi.org/10.1016/j.celrep.2020.107807.

68. Johnson, E.C.B., Dammer, E.B., Duong, D.M., Ping, L., Zhou, M., Yin, L., Higginbotham, L.A., Guajardo, A., White, B., Troncoso, J.C., et al. (2020). Large-scale proteomic analysis of Alzheimer's disease brain and cerebrospinal fluid reveals early changes in energy metabolism associated with microglia and astrocyte activation. Nat. Med. *26*, 769–780. https://doi.org/10.1038/s41591-020-0815-6.

69. Kelly, R.T. (2020). Single-cell proteomics: progress and prospects. Mol. Cell. Proteomics *19*, 1739–1748. https://doi.org/10.1074/mcp.r120.002234.

70. Aibar, S., González-Blas, C.B., Moerman, T., Huynh-Thu, V.A., Imrichova, H., Hulselmans, G., Rambow, F., Marine, J.-C., Geurts, P., Aerts, J., et al. (2017). SCENIC: single-cell regulatory network inference and clustering. Nat. Methods *14*, 1083–1086. https://doi.org/10.1038/nmeth.4463.

71. Jin, S., Guerrero-Juarez, C.F., Zhang, L., Chang, I., Ramos, R., Kuan, C.-H., Myung, P., Plikus, M.V., and Nie, Q. (2021). Inference and analysis of cell-cell communication using CellChat. Nat. Commun. *12*, 1088. https://doi.org/10.1038/s41467-021-21246-9.

72. Wolf, F.A., Angerer, P., and Theis, F.J. (2018). SCANPY: large-scale single-cell gene expression data analysis. Genome Biol. *19*, 15. https://doi.org/10.1186/s13059-017-1382-0.

73. Wolock, S.L., Lopez, R., and Klein, A.M. (2019). Scrublet: computational identification of cell doublets in single-cell transcriptomic data. Cell Syst. *8*, 281–291.e9. https://doi.org/10.1016/j.cels.2018.11.005.

74. Fleming, S.J., Marioni, J.C., and Babadi, M. (2019). CellBender remove-background: a deep generative model for unsupervised removal of background noise from scRNA-seq datasets. Preprint at bioRxiv. https://doi.org/10.1101/791699.

75. Jiang, R., Sun, T., Song, D., and Li, J.J. (2022). Statistics or biology: the zero-inflation controversy about scRNA-seq data. Genome Biol. *23*, 31. https://doi.org/10.1186/s13059-022-02601-5.

76. Beygelzimer, A., Kakadet, S., Langford, J., Arya, S., Mount, D., and Li, S. (2013). Fnn: fast nearest neighbor search algorithms and applications. R package version *1*, 1–17.

77. Zhang, B., and Horvath, S. (2005). A general framework for weighted gene Co-expression network analysis. Stat. Appl. Genet. Mol. Biol. *4*, Article17. https://doi.org/10.2202/1544-6115.1128.

78. Horvath, S., and Dong, J. (2008). Geometric interpretation of gene coexpression network analysis. PLoS Comput. Biol. *4*, e1000117. https://doi.org/10.1371/journal.pcbi.1000117.

79. Luecken, M.D., Büttner, M., Chaichoompu, K., Danese, A., Interlandi, M., Mueller, M.F., Strobl, D.C., Zappia, L., Dugas, M., Colomé-Tatché, M., and Theis, F.J. (2022). Benchmarking atlas-level data integration in single-cell genomics. Nat. Methods *19*, 41–50. https://doi.org/10.1038/s41592-021-01336-8.

80. Granja, J.M., Corces, M.R., Pierce, S.E., Bagdatli, S.T., Choudhry, H., Chang, H.Y., and Greenleaf, W.J. (2021). ArchR is a scalable software package for integrative single-cell chromatin accessibility analysis. Nat. Genet. *53*, 403–411. https://doi.org/10.1038/s41588-021-00790-6.

81. Melsted, P., Booeshaghi, A.S., Liu, L., Gao, F., Lu, L., Min, K.H.J., da Veiga Beltrame, E., Hjörleifsson, K.E., Gehring, J., and Pachter, L. (2021). Modular, efficient and constant-memory single-cell RNA-seq preprocessing. Nat. Biotechnol. *39*, 813–818. https://doi.org/10.1038/s41587-021-00870-2.

82. Traag, V.A., Waltman, L., and van Eck, N.J. (2019). From Louvain to Leiden: guaranteeing well-connected communities. Sci. Rep. *9*, 5233. https://doi.org/10.1038/s41598-019-41695-z. arXiv:1810.08473.

83. Plaisier, S.B., Taschereau, R., Wong, J.A., and Graeber, T.G. (2010). Rank–rank hypergeometric overlap: identification of statistically significant overlap between gene-expression signatures. Nucleic Acids Res. *38*, e169. https://doi.org/10.1093/nar/gkq636.

84. Blondel, V.D., Guillaume, J.-L., Lambiotte, R., and Lefebvre, E. (2008). Fast unfolding of communities in large networks. J. Stat. Mech. *2008*, P10008. https://doi.org/10.1088/1742-5468/2008/10/p10008.

85. Chen, E.Y., Tan, C.M., Kou, Y., Duan, Q., Wang, Z., Meirelles, G.V., Clark, N.R., and Ma'ayan, A. (2013). Enrichr: interactive and collaborative HTML5 gene list enrichment analysis tool. BMC Bioinf. *14*, 128. https://doi.org/10.1186/1471-2105-14-128.

STAR★METHODS

KEY RESOURCES TABLE

REAGENT or RESOURCE	SOURCE	IDENTIFIER
**Deposited data**		
Human Alzheimer's Disease snRNA-seq 2019	Mathys et al., 2019[10]	syn18485175
Human Autism spectrum disorder snRNA-seq	Velmeshev et al., 2019[9]	PRJNA434002
Human aging cortex snRNA-seq	authors	See Morabito et al., 2020[56]
Human Major depressive disorder snRNA-seq	Nagy et al., 2020[45]	GSE144136
Human Alzheimer's Disease snRNA-seq 2020	Zhou et al., 2020[11]	syn21670836
Mouse 5XFAD snRNA-seq 2020	Zhou et al., 2020[11]	syn21670836
Human Alzheimer's Disease Occipital Cortex snRNA-seq 2021	Gerrits et al., 2021[58]	GSE148822
Human Alzheimer's Disease Occipitotemporal Cortex snRNA-seq 2021	Gerrits et al., 2021[58]	GSE148822
Mouse hippocampus ScISOrSeq	authors	See Joglekar et al., 2021[8]
Human Alzheimer's Disease Entorhinal Cortex snRNA-seq 2021	Leng et al., 2021[57]	GSE147528
Human Alzheimer's Disease Superior Frontal Gyrus snRNA-seq 2021	Leng et al., 2021[57]	GSE147528
Mouse 5XFAD snRNA-seq 2022	authors	See Kiani-Shabestari et al., 2022[28]
10X Genomics mouse brain spatial transcriptomics	SeuratData R package	https://github.com/satijalab/seurat-data
Parse Biosciences PBMCs in Type 1 Diabetes	Parse Biosciences	https://resources.parsebiosciences.com/dataset-wt-mega-one-million-pbmc-type-1-diabetes
**Software and algorithms**		
hdWGCNA R package	this paper	https://doi.org/10.5281/zenodo.6835227
data analysis scripts	this paper	https://doi.org/10.5281/zenodo.7851151
WGCNA	CRAN	RRID:SCR_003302
Kallisto Bustools	https://github.com/pachterlab/kallistobustools	RRID:SCR_018213
Scanpy	Wolf et al., 2018[72]	RRID:SCR_018139
Seurat	CRAN	RRID:SCR_016341
Harmony	CRAN	RRID:SCR_022206
Scrublet	Wolock et al., 2019[73]	RRID:SCR_018098
Cellbender	Fleming et al., 2019[74]	https://github.com/broadinstitute/CellBender
SEACells	Persad et al., 2023[16]	https://github.com/dpeerlab/SEACells
Metacell-2	Ben-Kiki et al., 2022[14]	https://github.com/tanaylab/metacells
EGAD	Ballouz et al., 2016[23]	https://github.com/sarbal/EGAD
Enrichr	https://maayanlab.cloud/Enrichr/	RRID:SCR_001575
XGBoost	Chen and Guestrin, 2016[25]	https://xgboost.readthedocs.io/en/stable/
Monocle3	Cao et al., 2019[36]	https://cole-trapnell-lab.github.io/monocle3/
scDRS	Zhang et al., 2022[52]	https://github.com/martinjzhang/scDRS
SeuratData	Satija Lab	https://github.com/satijalab/seurat-data

RESOURCE AVAILABILITY

Lead contact

Requests for further information should be directed to the lead contact, Vivek Swarup (vswarup@uci.edu).

Materials availability

This study did not generate new unique reagents.

Data and code availability

- All of the sequencing data used in this paper were obtained from publicly available sources, and are listed in the key resources table.
- The hdWGCNA R package has been deposited at Zenodo (see key resources table). The R package code and full tutorials are available at https://swaruplab.bio.uci.edu/hdWGCNA. The data processing and analysis code has been deposited at Zenodo (see key resources table) and is available on GitHub at this repository: https://github.com/smorabit/hdWGCNA_paper.
- Any additional information required to reanalyze the data reported in this paper is available from the lead contact upon request.

METHOD DETAILS

Bootstrapped aggregation of single cell transcriptomes to form metacells

Single-cell gene expression datasets typically contain many more zero valued entries than non-zero valued entries, meaning that these datasets are sparse. We formally define the **sparsity** of a gene expression matrix in Equation 1. Given an un-normalized counts matrix X with genes and N_c cells, sparsity is the sum of all zero valued elements.

$$sparsity = \frac{\sum_{i=1}^{N_g}\sum_{j=1}^{N_c}\begin{cases}1 & \text{if } X_{i,j}=0\\ 0 & \text{else}\end{cases}}{N_g \times N_c} \qquad \text{(Equation 1)}$$

Complementing sparsity, the density of a single gene expression matrix is the sum of all non-zero valued elements divided by the total number of matrix elements, such that $density = 1 - sparsity$. A matrix is considered sparse if $sparsity > 0.5$. Conventional single-cell gene expression assays yield sparse gene expression matrices. In general, correlations of sparse vectors may lead to downstream conclusions that are not robust or reproducible. Thus, as part of the hdWGCNA workflow, we propose a bootstrapped aggregation (bagging) algorithm to construct a gene expression matrix M with considerably reduced sparsity prior to performing co-expression network analysis. Zero valued entries in a gene expression matrix have both biological and technical origins,[75] and it is important to prioritize preserving relevant biological signals while reducing technical noise. For example, a biological zero may be attributed to a gene that is only expressed in a given cell population, whereas a technical zero may arise from low sequencing depth.

We define the set of unique cell barcodes C and the set of unique genes G such that $\|C\| = N_c$ and $\|G\| = N_g$. Transcriptomically similar cells are identified in a dimensionally-reduced representation D of the gene expression matrix X using the k-nearest neighbors (KNN) algorithm,[76] yielding N_c sets of k cells. Inherently, there is overlap between these N_c sets of k neighboring cells, and we include a parameter m to control for the maximum allowable overlap. Cells are uniformly randomly sampled from C, and gene expression signatures from X are aggregated (sum or average) with their k nearest neighbors. A cell is skipped if its neighbors have too much overlap with the set of neighbors from previously selected cells, in order to reduce redundancy in the downstream metacell

Algorithm 1. ConstructMetacells

Require: X such that $\dim(X) = N_g, N_c$ ▷ gene expression matrix of N_g genes and N_c cells
Require: D such that $\dim(D) = c, d$ ▷ dimensional reduction of X, with N_c cells and d dimensions
Require: C ▷ the set of unique cell barcodes
Require: $k \geq 2$
Require: $m \geq 0$
Require: $t \geq 1$
$K \leftarrow \mathrm{KNN}(D,k)$ ▷ K is a matrix of N_c rows and k columns with the k nearest neighbors of each cell
$S \leftarrow [\varnothing]$ ▷ list containing barcodes of cells selected for aggregation, initialized as empty
$i \leftarrow 0$
while $i < N_c$ and $\|S\| < t$ **do**
$i \leftarrow i+1$
$c \leftarrow c \in_R C$ ▷ c is randomly sampled from C
$N_o \leftarrow \max(\|K_{c*} \cup K_{j*}\| \forall j \in S)$ ▷ the maximum number of overlapping neighbors between
c and barcodes in
S
if.
$N_o < m$ **then**
$S \leftarrow [S,c]$
end if.
$C \leftarrow C \backslash S$
end while.
$J \leftarrow [K_{s*} \forall s \in S]$ ▷ subset of K with the selected cells S
$M \leftarrow [\sum_{i=S_1}^{S}(X_{*s} \text{ where } s = J_{i*})]$. ▷ final metacell expression matrix

expression matrix. The cell sampling loop converges when there are no more cells that satisfy the m, or when the number of target metacells t has been reached, yielding a metacell gene expression matrix M. Sparsity of the input and output matrices X and M are computed to check that sparsity is reduced throughout this process. This metacell bagging algorithm is implemented as part of the hdWGCNA R package in the ConstructMetacells function, and the pseudocode for this algorithm is defined in Algorithm 1. We denote a vector containing the elements of the i-th row of a matrix as M_{i*} and a vector containing the elements of the i-th column as M_{*i}.

Aggregation of neighboring spatial transcriptomic spots to form metaspots

Sequencing-based ST approaches such as the 10× Genomics Visium platform also yield sparse transcriptomic profiles, thus introducing the same potential pitfalls as single-cell data for co-expression network analysis. To alleviate these issues, we sought to develop a data aggregation approach similar to our metacell algorithm. This approach leverages spatial coordinates rather than the dimensionality-reduced representation (Figure S6). For each ST spot, we obtain a list of physically neighboring spots. We then devise a grid of "principal spots", which are evenly spaced spots throughout the input tissue which serve as anchor points for aggregating neighboring spots. Each principal spot and its neighbors are aggregated into one metaspot, with at most seven spots merging into one metaspot and at most two overlapping spots between metaspots. We implemented this procedure as part of the hdWGCNA R package in the MetaspotsByGroups function. Similar to the MetacellsByGroups function, the user may specify groups within the Seurat object to perform the aggregation, such that metacells would only be grouped within the same tissue slice, anatomical region, or other annotation. For all downstream analysis with hdWGCNA, the metaspot expression dataset can be used in place of the metacell expression matrix.

Computing co-expression networks

Following metacell or metaspot construction, hdWGCNA constructs co-expression networks and identifies gene modules, building off of the WGCNA workflow.[1,4,77,78] The gene-gene adjacency matrix A is computed by taking the pairwise correlation of genes in G in the metacell expression matrix M, or in a subset of M for a specified cell population. Consider the gene expression vectors $x_i = M_{i*}$ and $x_j = M_{j*}$ for an arbitrary pair of genes $(i,j) \in G$, we compute the signed correlation as:

$$a_{i,j} = \frac{1+cor(x_i,x_j)}{2} \qquad \text{(Equation 2)}$$

Note that $a_{i,j}$ is a linear transformation that retains the sign of the correlation while satisfying $0 \le a_{i,j} \le 1$. We define A as a symmetric adjacency matrix of size $N_g \times N_g$ containing the signed correlations $a_{i,j}$ for all pairs $(i,j) \in G$ as in Equation 2. In order to emphasize strong correlations, we raise the elements of A to a power β, and we refer to this as soft power thresholding.

$$\begin{gathered} \alpha_{i,j} = (a_{i,j})^{\beta} \\ \tilde{\alpha}_{i,j} = \alpha_{i,j} \times \mathrm{sign}(\mathrm{cor}(x_i,x_j)) \end{gathered} \qquad \text{(Equation 3)}$$

Now we have the gene-gene correlation raised to a power β, and an alternative metric $\tilde{\alpha}_{i,j}$ which also retains the sign of the correlation between these genes. The final co-expression network is then computed as a signed topological overlap matrix (TOM). The TOM describes shared neighbors between the a pair of genes (i,j). We define the signed TOM as

$$\mathrm{TOM}_{i,j}^{\mathrm{signed}} = \frac{\left|\alpha_{i,j}+\sum_{u \ne i,j}\tilde{\alpha}_{i,u}\tilde{\alpha}_{u,j}\right|}{\min(k_i,k_j)+1 - |\alpha_{i,j}|} \qquad \text{(Equation 4)}$$

where k_i and k_j represent the connectivity between genes i and j

$$k_i = \sum_{u \ne i} |\tilde{a}_{u,i}| \qquad \text{(Equation 5)}$$

In the signed TOM, negative correlations serve to negatively reinforce the network connection, which is not the case in the unsigned TOM.

$$\mathrm{TOM}_{i,j}^{\mathrm{unsigned}} = \frac{|\alpha_{i,j}|+\sum_{u \ne i,j}|\tilde{\alpha}_{i,u}\tilde{\alpha}_{u,j}|}{\min(k_i,k_j)+1 - |\alpha_{i,j}|} \qquad \text{(Equation 6)}$$

Genes are then grouped into modules based on the TOM network representation using the Dynamic Tree Cut algorithm,[3] such that co-expression modules consist of genes with high topological overlap. Dynamic Tree Cut hierarchically clusters genes based on their dissimilarity in the TOM, denoted as DissTOM = 1 − TOM, thereby yielding a mapping between module assignments and gene names. The overall process transforming a metacell expression matrix M to a signed TOM co-expression network is implemented as part of the hdWGCNA R package in the ConstructNetwork function. Here we described the recommended workflow, using a signed adjacency matrix and a signed TOM, but ConstructNetwork can optionally construct unsigned or signed hybrid networks as well.

CellPress
OPEN ACCESS

Computing module eigengenes

Module eigengenes (MEs) are a convenient metric to summarize the gene expression of a given co-expression module. While the co-expression network was computed using the metacell expression matrix M, we compute MEs in the single-cell expression matrix X, thus yielding information about the activity of each module in each cell. The expression matrix for the l-th module consisting of genes $G^{(l)} \subset G$ is $X^{(l)} = X_{G^{(l)},*}$. The ME for module l is then computed by performing singular value decomposition (SVD), such that $X^{(l)} = UDV^T$. Prior to running SVD, $X^{(l)}$ must be scaled and centered, and we accomplish this using the Seurat function ScaleData. Importantly, ScaleData enables us to optionally perform regression to diminish the effects of selected technical covariates prior to computing MEs. The first column of V, containing the right-singular vectors $V^{(l)} = (v_1^{(l)}, v_2^{(l)}, v_3^{(l)}, \ldots)$, is the ME of module l.

$$\mathrm{ME}^{(l)} = v_1^{(l)} \qquad \text{(Equation 7)}$$

While SVD or other dimensionality reductions on a single-cell gene expression matrix contains critical biological information, technical artifacts are also present in these representations. There are many computational methods aiming to reduce technical effects in a reduced dimensional space, and these methods are often referred to as "batch-correction" or "integration" approaches.[79] In particular, Harmony[22] is an algorithm well suited for correcting batch effects that may be present in a dimensionality-reduced single-cell expression dataset,[79] and here we propose applying Harmony to MEs to maximize the biological information content of each ME. We implemented the ME computation algorithm, as defined in Algorithm 2, as part of the hdWGCNA R package in the function ModuleEigengenes.

Algorithm 2. ModuleEigengenes

Require: X such that $\dim(X) = N_g, N_c$ ▷ normalized gene expression matrix of N_g genes and N_c cells
Require: modules ▷ the table containing mappings between genes and modules.
Require: mods ▷ list of modules.
Require: covariates ▷ covariates to regress.
Require: batches ▷ batch identity to correct with Harmony, or null to ignore.
ME $\leftarrow [\varnothing]$
for l in mods **do**
modules $^{(l)} \leftarrow$ subset(modules, module = = l)
$G^{(l)} \leftarrow$ modules $^{(l)}$ [,gene]
$X^{(l)} \leftarrow X_{G^{(l)},*}$
$\tilde{X}^{(l)} \leftarrow$ ScaleData($X^{(l)}$, covariates)
$V^{(l)} \leftarrow$ SVD $(\tilde{X}^{(l)})$
ME $^{(l)} \leftarrow V_1^{(l)}$
if batches $\neq$ NULL **then**
$\tilde{V}^{(l)} \leftarrow$ Harmony($V^{(l)}$, batches)
ME $^{(l)} \leftarrow \tilde{V}_1^{(l)}$
end if.
ME $\leftarrow$ [ME, ME $^{(l)}$]
end for.

Projecting co-expression modules in unseen data

In a typical hdWGCNA workflow, we perform metacell bagging, co-expression network analysis, module identification, and ME computation using the same single-cell gene expression dataset, starting from the expression matrix X. Given the module-gene assignment table derived from a reference dataset X, we can run the ModuleEigengenes algorithm on a query dataset Y where the genes in Y must be contained in the set of genes in X such that $G_Y \subseteq G_X$. We implemented this process in the hdWGCNA R package as the ProjectModules function. Importantly, we designed ProjectModules to be agnostic towards the data modality or species used in the reference and query datasets, thereby allowing for a host of comparative analyses. ProjectModules can facilitate cross-species analysis leveraging a table that maps gene symbols between two genomes. Modules can be projected into epigenomic data modalities such as single-cell assay for transposase accessible chromatin with sequencing (scATAC-seq) provided a measure of gene expression estimated from chromatin accessibility, such as Signac[59] gene activity or ArchR[80] gene scores. This approach can also be used to project modules from bulk expression datasets into single-cell or spatial transcriptomics datasets.

Implementation of the hdWGCNA R package

hdWGCNA greatly extends upon scWGCNA,[12] our previous method for co-expression network analysis in single-cell transcriptomics data. scWGCNA was originally used to identify co-expression networks using bulk and single-cell RNA-seq together,[12] and in another study we showed that scWGCNA was suitable for network analysis using scRNA-seq alone.[28] Contrasting the hdWGCNA

package, the implementation of scWGCNA was an R package containing a single function for metacell construction, and a single tutorial to cover the basics of network analysis using the WGCNA package[1] with the metacell matrix. We implemented hdWGCNA as an open-source object-oriented R package that leverages the widely used SeuratObject data structure. The hdWGCNA R package includes all necessary functions for network inference, data visualization, statistical testing, and downstream analysis such as pathway enrichment. Further, hdWGCNA includes functions to extract the network data from the SeuratObject to easily facilitate custom analysis with external Bioconductor or R packages. In order for hdWGCNA to be widely useful across the genomics community, we developed a detailed documentation website containing tutorials for network analysis in single-cell and spatial transcriptomics data, as well as tutorials for advanced analysis like consensus network analysis and network preservation testing. Unlike scWGCNA, the metacell construction algorithm (Algorithm 1) in hdWGCNA includes new parameters to avoid redundant metacells, the module eigengene algorithm in hdWGCNA (Algorithm 2) accounts for batch effects and additional covariates in the input dataset, and hdWGCNA contains functions to handle spatial transcriptomics datasets. Several key steps in co-expression network analysis, like calculating module eigengenes and eigengene-based connectivity, have been re-implemented to operate on sparse matrices, greatly decreasing runtime and memory usage. hdWGNCA is completely technology agnostic, and can be adapted to handle high dimensional counts matrices from any single-cell or spatial transcriptomics platform. Additionally, hdWGCNA includes a novel approach for visualizing genes and the underlying network in a two-dimensional manifold of co-expression space using UMAP.[19] As shown throughout this manuscript, hdWGCNA includes functions for projecting co-expression networks into a variety of external datasets. The widespread adoption of single-cell genomics has led to many biologists running their own computational analysis, and we designed the hdWGCNA R package with these individuals in mind through our various step-by-step tutorials and detailed documentation.

QUANTIFICATION AND STATISTICAL ANALYSIS

Reprocessing published datasets

The key resources table details the different datasets used throughout this manuscript. We used several published datasets generated by our own group,[12,28,56] and sequencing data was not re-downloaded for these studies. For all human snRNA-seq datasets, we applied a uniform processing pipeline to process each dataset starting from the raw sequencing data and resulting in an anndata object[72] containing UMI counts, normalized gene expression, cluster identities, and cell type annotations. Parameters used throughout this processing pipeline vary slightly between different datasets, and all parameters are noted in the data processing scripts in our github repository. For each biological replicate, we used the kb count function from kallisto | bustools[81] to psuedoalign raw sequencing reads to the reference transcriptome and quantify gene expression attributed to each cell barcode. The human reference transcriptome (GRch38) was obtained from the 10× Genomics website (version 2020-A, July 2020), and was re-formatted for use with kallisto | bustools using the kb ref function. For each of the UMI counts matrices, we used the remove-background function from cellbender[74] to simultaneously identify which barcodes corresponded to cells and to remove counts attributed to ambient RNA. We then used scrublet[73] to compute ''doublet scores'', the likelihood of each barcode mapping to more than one cell. Counts matrices from each biological replicate in a given dataset are then merged into a single anndata object, and any relevant sample level meta-data (age, sex, disease status) was stored in the adata.obs table. We performed a percentile filtering of cells that were outliers from each dataset based on the number of UMI per cell the percentage of UMI attributed to mitochondrial genes per cell, and the doublet score. Filtering based on these criteria was performed in each sample, as well as dataset-wide. After filtering, downstream data processing steps were carried out with SCANPY.[72] The UMI counts matrix was normalized with $\ln(\mathrm{CPM})$ using the functions sc.pp.normalize_total and sc.pp.log1p. Highly variable genes were identified using the function sc.pp.highly_variable_genes, and these genes are used as the features for downstream analysis steps such as principal component analysis (PCA). The normalized expression matrix was then scaled to unit variance and centered at zero using the function sc.pp.scale. PCA was performed on the scaled expression matrix using the function sc.tl.pca. Harmony[22] was used to correct the PCA matrix for batch effects using the function sc.external.pp.harmony_integrate. The harmonized PCA matrix was then used to construct a cell neighborhood graph using the function sc.pp.neighbors. The cell neighborhood graph was then used to compute a two-dimensional representation of the data with uniform manifold approximation and projection[19] using the function sc.tl.umap, and to group cells into clusters with Leiden clustering[82] using the function sc.tl.leiden. We inspected the gene expression signatures in each Leiden cluster for a panel of canonical cell-type marker genes in order to assign a cell-type label to each cluster, and to identify additional doublet clusters that may have escaped the previous filtering steps. The distribution of quality control metrics was inspected in each cluster. We filtered out cells belonging to clusters that displayed conflicting expression of cell-type marker genes, or were outliers in their quality control metrics. After filtering these low-quality clusters, we ran UMAP and Leiden clustering again, resulting in the final processed dataset. We used a custom script to convert the datasets from *anndata* to SeuratObject by saving the individual components (counts matrix, cell metadata, gene meta-data, dimensionality reductions, etc.) in Python and then loading them back into R to create a SeuratObject.

Iterative network analysis of major cell types in the human cortex

We performed an iterative co-expression network analysis of the major cell types (ASC, EX, INH, MG, ODC, OPC) in the human PFC snRNA-seq dataset from Zhou et al.,[11] only including samples from control brains (36,671 cells and 36,601 genes). We retained genes that were expressed in at least 5% of cells for downstream analysis. Metacells were computed separately for each major

cell type and each sample using the hdWGCNA function MetacellsByGroups, aggregating 25 cells per metacell. Further, we ran MetacellsByGroups while varying the *K* parameter in order to asses the resulting metacell expression matrix sparsity. For each cell type, we applied the following hdWGCNA commands with default arguments to perform network analysis: TestSoftPowers, ConstructNetwork, ModuleEigengenes, ModuleConnectivity, and RunModuleUMAP. We performed module preservation analysis[20] of the ODC co-expression modules in an external snRNA-seq dataset of the human PFC.[12] Modules were projected from the reference to query dataset using the hdWGCNA function ProjectModules, and the module preservation test was performed using ModulePreservation with 100 permutations.

Comparison of hdWGCNA with alternative metacell approaches

For the purpose of co-expression network analysis, we compared our metacell aggregation approach (Algorithm 1) with two alternative approaches, namely Metacell2[14] and SEACells.[16] We ran the three metacell approaches using the recommended settings on the same dataset, and then ran hdWGCNA on each of the resulting metacell expression matrices. We used a scRNA-seq of 6,800 $CD34^+$ hematopoietic stem and progenitor stem cells included with the SEACells package, and we used the cluster annotations from the original study. Notably, SEACells and Metacell2 do not account for cell labels in their aggregation procedures, which may result in a number of metacells containing transcriptomes from differently labeled cells. For the hdWGCNA metacell algorithm, we aggregated 50 cells per metacell. For the three metacell expression matrices derived from the different algorithms, we performed co-expression network analysis with the standard hdWGCNA pipeline by sequentially running the following functions with default parameters: TestSoftPowers, ConstructNetwork, ModuleEigengenes, ModuleConnectivity, and RunModuleUMAP. With the same cluster settings, Dynamic Tree Cut recovered a different number of co-expression modules for the three methods (hdWGCNA: 16 modules; MC2: 13 modules; SEACells: 20 modules). We performed pairwise comparisons between the gene modules detected with each metacell approach using Fisher's exact test to test module overlaps. Additionally, we performed rank-rank hypergeometric overlap[83] (RRHO) tests using the RRHO function from the R package RRHO (version 1.13.0) to compare the kME ranking between modules across methods. To compare MEs and Seurat module scores, we ran the AddModuleScore function, and computed Pearson correlations between each ME and each module score.

Application of hdWGCNA to a one million cell scRNA-seq dataset

We obtained a publicly available scRNA-seq dataset from Parse Biosciences of 1M peripheral blood mononuclear cells (PBMCs) from twelve healthy donors and twelve Type-1 diabetic donors generated using the Evercode Whole Transcriptome Mega protocol. This analysis was performed on a compute cluster with 200 GB of memory and eight CPU cores. The UMI counts matrix and sample meta data was downloaded from Parse Biosciences' Website. We processed the counts matrix using SCANPY using a similar pipeline as described in the reprocessing published dataset section. For quality control, we excluded cells with greater than 25% mitochondrial reads, greater than 5,000 genes, and greater than 25,000 counts. After dimensionality reduction with PCA, Harmony[22] batch correction, and Leiden clustering[82] (resolution = 1), we annotated cell populations using PBMC marker genes obtained from Azimuth.[7] We excluded clusters with conflicting cell-type markers as potential doublet populations, retaining a total of 965,363 cells and 26,862 genes for downstream analysis. The major cell compartments recovered in this analysis were similar to those reported by Parse Biosciences in their analysis, including as T-cells, B-cells, monocytes, dendritic cells, basophils, and plasmablasts. Following the SCANPY data processing, we wrote the individual components (counts matrix, cell meta-data, gene meta-data, dimensionality reductions, etc.) to disk so they could be loaded into R and assembled into a Seurat object.

We performed co-expression network analysis iteratively for the plasmablast, T-cell, B-cell, monocyte, and dendritic cell compartments using an hdWGCNA pipeline for each group (Figure S5). Metacells were constructed separately for each sample and each cell cluster with the hdWGCNA function MetacellsByGroups, aggregating 50 cells per metacell. The metacell aggregation step had a runtime of 85 min and 59 s. For each cell population, we first subset the Seurat object for the cell population of interest and then performed the standard hdWGCNA pipeline by sequentially running the following functions with default parameters: TestSoftPowers, ConstructNetwork, ModuleEigengenes, ModuleConnectivity, and RunModuleUMAP. We note that for the largest cell population (T-cells, 555,417 cells), the runtime for the network construction step was 186 s.

Runtime and memory usage of hdWGCNA

We tested the runtime and memory usage of the primary co-expression network analysis functions in hdWGCNA using the Velmeshev et al. 2019[9] dataset. We selected the neuronal cell population from the dataset for network analysis, and downsampled the dataset at different sizes ranging from 1,000 to 50,000 cells to test the runtime and memory usage as a function of the number of cells in the input dataset. The following functions were tested: SetupForWGCNA, MetacellsByGroups, TestSoftPowers, ModuleEigengenes, and ModuleConnectivity. We tested ModuleEigengenes with and without Harmony correction. All of these tests were done using eight parallel threads, and hdWGCNA can be sped up further by increasing the number of parallel threads. Importantly, the number of input genes and other network analysis parameters also have an effect on runtime and memory usage.

Evaluating performance of hdWGCNA co-expression networks

We tested the functional coherence of hdWGCNA co-expression networks using the Extending 'Guilt-by-Association' by Degree (EGAD)[23] algorithm. Connected genes in biological networks are potentially involved in the same processes, and EGAD evaluates

this network property given a set of gene-process annotations. We performed functional coherence testing with the EGAD R package (version 1.18.0) using the six cell-type-specific co-expression networks from the Zhou et al. 2020[11] human PFC dataset. We downloaded a table of gene ontology associations for each gene from ensembl biomart, and formatted this table using the EGAD function make_annotations. We then ran the functional coherence test with EGAD using the function run_GBA, using the TOM as the input network, and we report the distributions of area under the receiver operating characteristic curve (AUC) values for each tested biological process in the six co-expression networks.

We used the xgboost R package[25] (version 1.7.3.1) to perform XGBoost regularized regression analysis to predict a given gene's expression based on the expression of the top ten module hub genes for the module each gene was assigned to. This analysis was done using the six cell-type-specific co-expression networks described in the iterative network analysis of major cell types in the human cortex section. We performed 5-fold cross validation, and measured the performance of the model as a test set root-mean-square error (RMSE) averaged across the 5-folds. We ran XGBoost for 100 iterations for each individual test, with a maximum tree depth of 3 and regularization alpha of 0.5.

Spatial co-expression network analysis in the mouse brain

We collected the publicly available 10× Genomics Visium mouse brain dataset using the SeuratData R package. This dataset consists of an anterior and a posterior slice from a sagittal brain section, which we merged into a single Seurat object comprising 6,049 ST spots and 31,053 genes. We processed this dataset using the standard Seurat pipeline by sequentially running the following commands: NormalizeData, FindVariableFeatures, ScaleData, RunPCA, FindNeighbors, FindClusters, and RunUMAP. The top thirty PCs were used for Louvain clustering[84] and UMAP. While ST spots were clustered based on transcriptomic information alone, we were able to annotate them based on anatomical features.

Neighboring ST spots were aggregated into metaspots in the anterior and posterior slices using the hdWGCNA function MetaspotsByGroups. We retained genes expressed in 5% of spots for downstream analysis, totaling 12,355 genes. We tested for the optimal soft-power threshold β based on the the fit to a scale-free topology using the hdWGCNA function TestSoftPowers. The co-expression network was constructed using all ST spots spanning both the anterior and posterior slices using the hdWGCNA function ConstructNetwork with the following parameters: networkType = "signed", TOMType = "signed", soft_power = 5, deepSplit = 4, detectCutHeight = 0.995, minModuleSize = 50, mergeCutHeight = 0.2. Module eigengenes and eigengene-based connectivities were computed using the ModuleEigengenes and ModuleConnectivity functions respectively. This approach identified 12 spatial co-expression modules, and we visualized the spatial distributions of these modules by plotting their MEs directly onto the biological coordinates for each spot. The co-expression network was projected into two dimensions using UMAP with the hdWGCNA function RunModuleUMAP, and we used the top five hub genes (ranked by kMEs) as the input features for UMAP. We used the R package enrichR[85] (version 3.0) to perform enrichment analysis on the top 100 genes in each module ranked by kME using the following databases: GO_Biological_Process_2021, GO_Cellular_Component_2021, GO_Molecular_Function_2021, WikiPathway_2021_Mouse, and KEGG_2021_Mouse. We assessed the overlap between genes from these spatial co-expression modules and differentially expressed genes in each cluster from a recent snRNA-seq study of the whole mouse brain using Fisher's exact test implemented in the R package GeneOverlap (version 1.26.0). Finally, we performed a separate network analysis on a subset of the ST dataset only containing the cortical layers 2–6, and we followed an identical hdWGCNA analysis pipeline to the full ST dataset for the cortical analysis.

Isoform co-expression network analysis in the mouse hippocampus

We performed isoform co-expression network analysis in radial glia lineage cells (radial glia, astrocytes, ependymal cells, and neural intermediate progenitor cells) from mouse hippocampus ScISOrSeq dataset from Joglekar et al.[8] using the hdWGCNA R package. The gene-level counts matrix for this dataset was obtained from the Gene Expression Omnibus database (GEO: GSE15845), and the isoform-level counts matrix was obtained directly from the authors of the original study. We formatted this dataset as a Seurat object with an isoform-level expression assay and a gene-level expression assay. The standard Seurat processing pipeline was used on the gene-level expression assay, where we sequentially ran the functions NormalizeData, FindVariableFeatures, ScaleData, and RunPCA with default parameters. The dataset was projected into two dimensions by running UMAP on the PCA matrix with 30 components using the RunUMAP function. For all downstream purposes, the cell-type annotations from the original study were used.

Radial glia cells were selected for network analysis, and isoforms expressed in fewer than 1% of these cells were excluded, yielding a set of 2,190 cells and 10,375 isoforms from 4,770 genes. We constructed metacells separately for each cell type on the isoform-level expression assay using the hdWGCNA function MetacellsByGroups with $k = 30$. We performed a parameter sweep for the soft-power threshold β using the function TestSoftPowers. The isoform co-expression network was constructed using the ConstructNetwork function with the following parameters: networkType = "signed", TOMType = "signed", soft_power = 5, deepSplit = 4, detectCutHeight = 0.995, minModuleSize = 50, mergeCutHeight = 0.5. This approach identified 11 isoform co-expression modules. Isoform-level module eigenisoforms were computed using the ModuleEigengenes function, and eigenisoform-based connectivity was computed using the ModuleConnectivity function with default parameters. We computed a semi-supervised UMAP projection of the co-expression network using the hdWGCNA function RunModuleUMAP, with the module labels and the top six

CellPress
OPEN ACCESS

hub isoforms (by kMEiso) per module as the input features. We used the enrichR to identify enriched pathways in each module ranked by using the following databases: GO_Biological_Process_2021, GO_Cellular_Component_2021, GO_Molecular_Function_2021, WikiPathway_2021_Mouse, and KEGG_2021_Mouse.

To assess isoform co-expression network dynamics throughout the cellular trajectories within the radial glia lineage, we performed pseudotime analysis using Monocle 3[36] (version 1.0.0). We computed a UMAP of just radial glia lineage cells using the Monocle 3 function run_umap. A trajectory graph was built on this UMAP representation using the function learn_graph, and pseudotime was calculated with the function order_cells using the radial glia cells as the starting point. We split the pseudotime trajectory into three lineages based on the distinct cell fates (astrocyte, neuronal, and ependymal). We grouped cells into 50 evenly-sized bins throughout each trajectory, and we applied loess regression to the average module eigenisoform of each module in these bins to inspect the dynamics of each module throughout development. We wrote a custom script to generate a GTF of isoform models output from the ScISOrSeq pipeline. To visualize expressed isoforms, we plotted isoforms from this GTF on the UCSC genome browser as well as in Swan.[38]

Co-expression analysis network of inhibitory neurons in autism spectrum disorder

We selected inhibitory neurons from the Velmeshev et al.[9] human autism spectrum disorder (ASD) snRNA-seq dataset for co-expression network analysis. Of the 121,451 cells in this dataset, 20,249 were labeled as inhibitory neurons based on marker gene expression profiles. We retained 11,194 genes which were expressed in at least 10% of cells from any cluster, and had non-zero variance in the inhibitory neuron population. Metacell transcriptomic profiles were constructed separately for each of the 54 samples and each cell type using the hdWGCNA function MetacellsByGroups, aggregating 50 cells into one metacell. We selected a soft-power threshold $\beta = 9$ based on the parameter sweep performed with the TestSoftPowers function. The co-expression network was computed with the ConstructNetwork function with the following parameters: networkType = "signed", TOMType = "signed", soft_power = 9, deepSplit = 4, detectCutHeight = 0.995, minModuleSize = 50, mergeCutHeight = 0.2. Module eigengenes were computed using the ModuleEigengenes function, and we applied Harmony[22] to correct MEs based on sequencing batch. Eigengene-based connectivity for each gene was computed using ModuleConnectivity. The co-expression network was embedded in two dimensions using UMAP with the RunModuleUMAP function with the top five genes (ranked by kMEs) per module as the input features. Distributions of MEs were compared between ASD and control samples for each inhibitory neuron subpopulation using a two-sided Wilcoxon rank-sum test with the R function wilcox.test. We used the enrichR[85] to perform enrichment analysis on the top 100 genes in each module ranked by kME using the following databases: GO_Biological_Process_2021, GO_Cellular_Component_2021, GO_Molecular_Function_2021, WikiPathway_2021_Human, and KEGG_2021_Human. Furthermore, we computed the overlap between co-expression modules and ASD-associated genes from the SFARI Gene database using the R package GeneOverlap, which calculates the overlap between sets of genes using Fisher's exact test.

Consensus co-expression network analysis of microglia in Alzheimer's disease

We performed consensus co-expression network analysis of microglia in Alzheimer's disease (AD) using three published snRNA-seq datasets.[10–12] The individually processed datasets were merged into a single Seurat object comprising 189,127 nuclei, and the datasets were integrated into a common dimensionally-reduced space using PCA and Harmony.[22] We retained all nuclei labeled microglia for network analysis based on expression of canonical marker genes such as *CSF1R* (9,904 nuclei), and genes expressed in at least 5% of microglia from any of the three studies were retained (7,900 genes). Metacells were constructed in groups of cells based on AD diagnosis status and study of origin, aggregating 25 cells per metacell. Within hdWGCNA, we used the SetMultiExpr function to create a list of expression matrices containing the selected genes and metacells for the three studies. We performed a separate parameter sweep for the three expression matrices using the hdWGCNA function TestSoftPowerConsensus, ensuring that we used an appropriate β value for each dataset (Mathys et al.: $\beta = 6$, Zhou et al.: $\beta = 8$, Morabito & Miyoshi et al.: $\beta = 6$). The consensus co-expression network was contructed using the hdWGCNA function ConstructNetwork using the consensus = TRUE option. Individual TOMs were computed for each dataset, and they were scaled based on the 80th percentile in order to alleviate different statistical properties specific to each dataset rather than the underlying biology. A consensus TOM was computed by taking the element-wise minimum of the individual TOMs from each dataset. Therefore, large topological overlap values between two genes, which indicate a strong co-expression relationship, are supported across all three datasets in the consensus TOM. We performed hierarchical clustering on the consensus TOM, and we used the Dynamic Tree Cut algorithm[3] was used to identify consensus co-expression modules based on the hierarchy. Module eigengenes were computed using the ModuleEigengenes function, and we applied Harmony[22] to correct MEs based on the dataset of origin. Eigengene-based connectivity for each gene was computed using ModuleConnectivity. We visualized the network using UMAP with the top ten hub genes (ranked by kMEs) per module as the input features, annotating the hub genes and known disease-associated microglia genes.[50] We used the enrichR[85] to perform enrichment analysis on the top 100 genes in each module ranked by kME using the following databases: GO_Biological_Process_2021, GO_Cellular_Component_2021, GO_Molecular_Function_2021, WikiPathway_2021_Human, and KEGG_2021_Human.

We sought to model the transcriptional dynamics governing the shift between homeostatic and activated microglia in AD, therefore we performed pseudotime analysis using Monocle 3[36] to build a continuous trajectory of microglia cell states. A trajectory graph was built on the microglia UMAP using the function learn_graph, and pseudotime was calculated with the function order_cells. We oriented the start of pseudotime based on the expression of homeostatic microglia marker genes, such as *P2RY12*, *CX3CR1*, and

CSF1R. We grouped cells into 50 evenly-sized bins throughout each trajectory, and we applied loess regression to the average module eigengene of each module in these bins to inspect the dynamics of each module throughout the microglia trajectory.

To link the integrated microglia snRNA-seq dataset with polygenic risk of disease for individual cells, we used the scDRS python package (version 1.0.0).[52] This pipeline takes 1) a set of putative disease genes derived from GWAS summary statistics and 2) a scRNA-seq dataset as inputs, and outputs disease enrichment statistics for a given disease (raw and normalized disease scores, cell-level scDRS p value, and *Z*-scores converted from the p values). GWAS summary statistics of 74 diseases and complex traits supplied by scDRS were utilized as gene sets, among which a gene set by Jansen et al.[46] provided the set of genes associated with AD. We then visualized the AD scDRS *Z*-scores in the integrated AD microglia trajectory, and we correlated the scDRS score with the trajectory using a Pearson correlation.

We performed module preservation[20] analysis in a variety of external datasets from human and mouse[11,12,28,56–58] to test for the reproducibility of the consensus AD microglia modules in the microglia population from each dataset. We used the hdWGCNA function ProjectModules to compute module eigengenes for the consensus AD microglia modules for each query dataset. The module preservation test was performed using the hdWGCNA function ModulePreservation with 100 permutations, and we reported the preservation *Z*-summary statistics in a heatmap. For the Morabito & Miyoshi et al. snATAC-seq dataset, we used the gene activity[59] representation as a gene-level summary of chromatin accessibility in order to assess the module preservation at the epigenomic level.

Analysis of bulk RNA-seq co-expression modules in single-cell data

We projected gene co-expression modules from two bulk RNA-seq studies of AD[56,63] into a published snRNA-seq study of AD to assess their expression patterns within various cell populations. While both of these studies used the samples from the same bulk RNA-seq cohort, the set of modules from Morabito et al. 2020[56] was based on a consensus network analysis across six brain regions while the other set of modules from the AMP-AD study[63] were constructed separately for seven different brain regions. Module eigengenes were computed for each of these bulk RNA-seq modules in the snRNA-seq dataset using the hdWGCNA function ProjectModules, using Harmony to correct MEs based on sequencing batch. We visualized the MEs of the projected modules in the snRNA-seq dataset using the Seurat function DotPlot.

Cell Reports Methods, Volume *3*

Supplemental information

hdWGCNA identifies co-expression networks in high-dimensional transcriptomics data

Samuel Morabito, Fairlie Reese, Negin Rahimzadeh, Emily Miyoshi, and Vivek Swarup

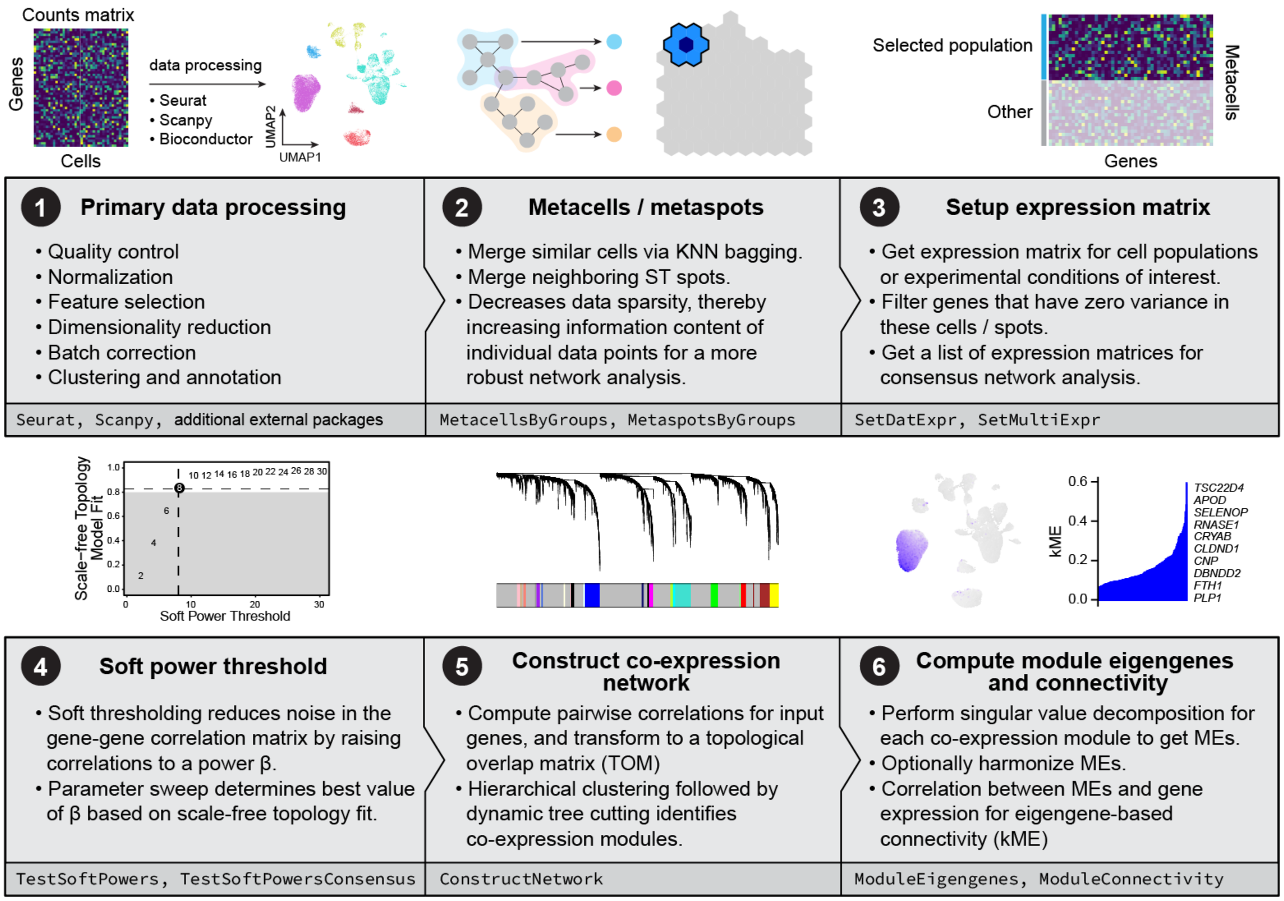

Figure S1. Schematic of the hdWGCNA workflow, related to Figure 1. 1. Prior to analysis with the hdWGCNA R package, the input single-cell or spatial dataset must be fully processed. This includes quality control, data normalization, feature selection, dimensionality reduction, batch correction (if needed), and clustering. These steps can be done using popular packages such as Seurat [1–3] or SCANPY [4]. Regardless of the pipeline used, the dataset must be formatted as a Seurat object prior to running hdWGCNA. **2.** The functions `MetacellsByGroups` and `MetaspotsByGroups` are used to aggregate transcriptomically similar cells into metacells and spatially proximal spots into metaspots respectively. **3.** hdWGCNA requires the user to explicitly specify the expression matrix that will be used for network analysis using the function `SetDatExpr`. For consensus network analysis, a list of expression matrices for each dataset or condition is specified with `SetMultiExpr`. **4.** Different values for the soft-power threshold β are tested using the functions `TestSoftPowers` and `TestSoftPowersConsensus`. The gene-gene correlation adjacency matrix is computed and raised to a power β as a soft threshold, and the degree distribution of this augmented is fit to a power law distribution to assess the scale-free topology. **5.** Co-expression network computation and gene module detection is performed in one step using the function `ConstructNetwork`. **6.** Module eigengenes (MEs) are computed using the `ModuleEigengene` function, optionally allowing for regression and harmonization of covariates such as sequencing batch. Eigengene-based connectivity (kME) is computed for each gene using the `ModuleConnectivity` function.

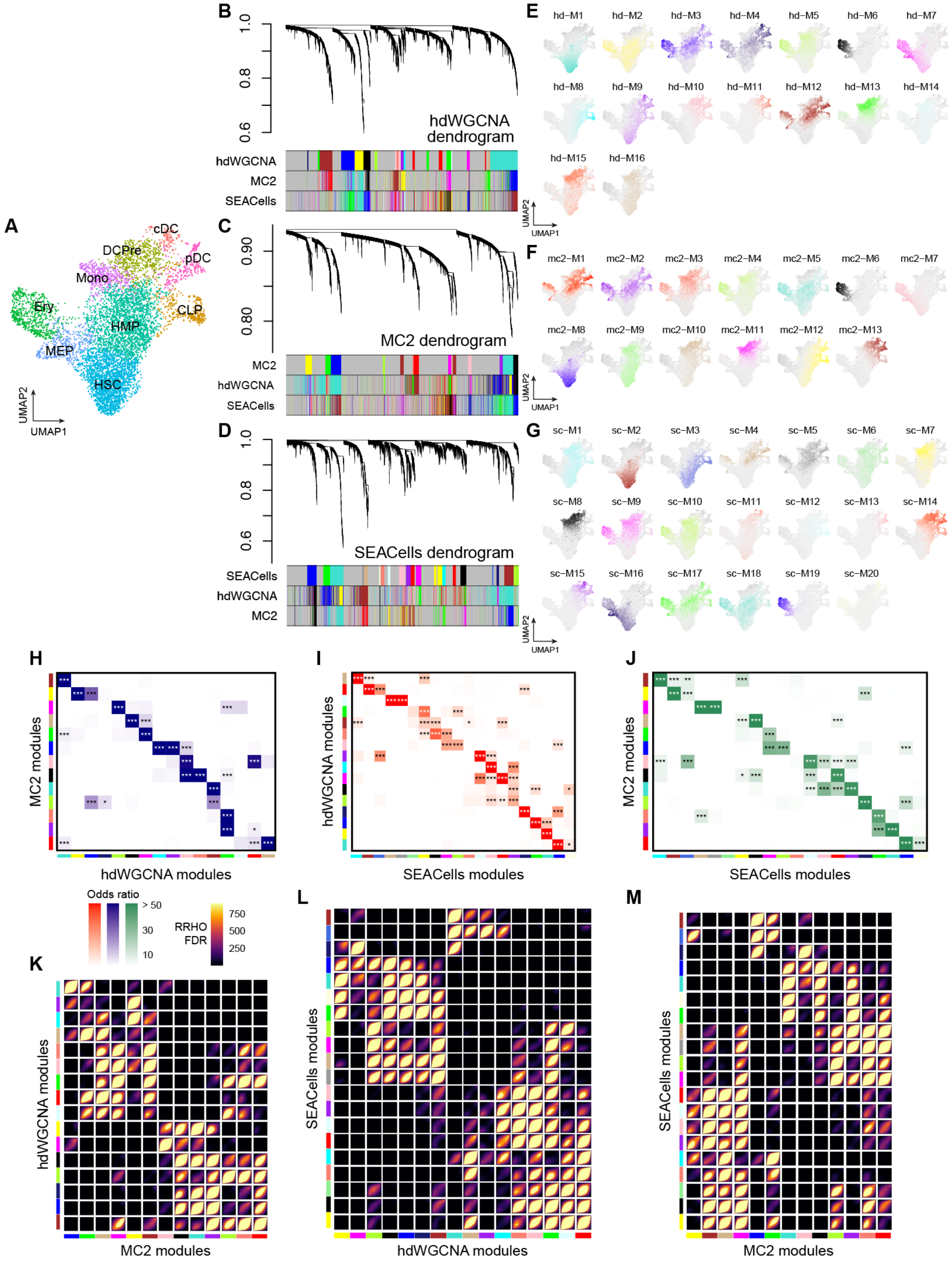

Figure S2. Comparison of metacell algorithms for co-expression network analysis. Caption on the next page ⟶

Figure S2. Comparison of metacell algorithms for co-expression network analysis, related to Figure 1. A. UMAP plot of the 6,800 CD34+ hematopoietic stem and progenitor stem cells scRNA-seq dataset[5] colored by annotations from the original study. **B-D.** hdWGCNA dendrograms for the co-expression networks constructed with the hdWGCNA (**B**), MC2 (**C**), and SEACells (**D**) algorithms. Module assignments are shown below the dendrograms **E-G.** UMAP plots as in (**A**) colored by MEs for the co-expression modules derived from the different metacell approaches. **H-J.** Module overlap comparisons between the different methods. Test was performed using Fisher's exact test, and we report the odds ratio and FDR corrected p-values. **K-M.** Rank-rank hypergeometric overlap (RRHO)[6] heatmaps comparing the ranks of kMEs for pairs of modules derived from the expression matrices from the different metacell algorithms.

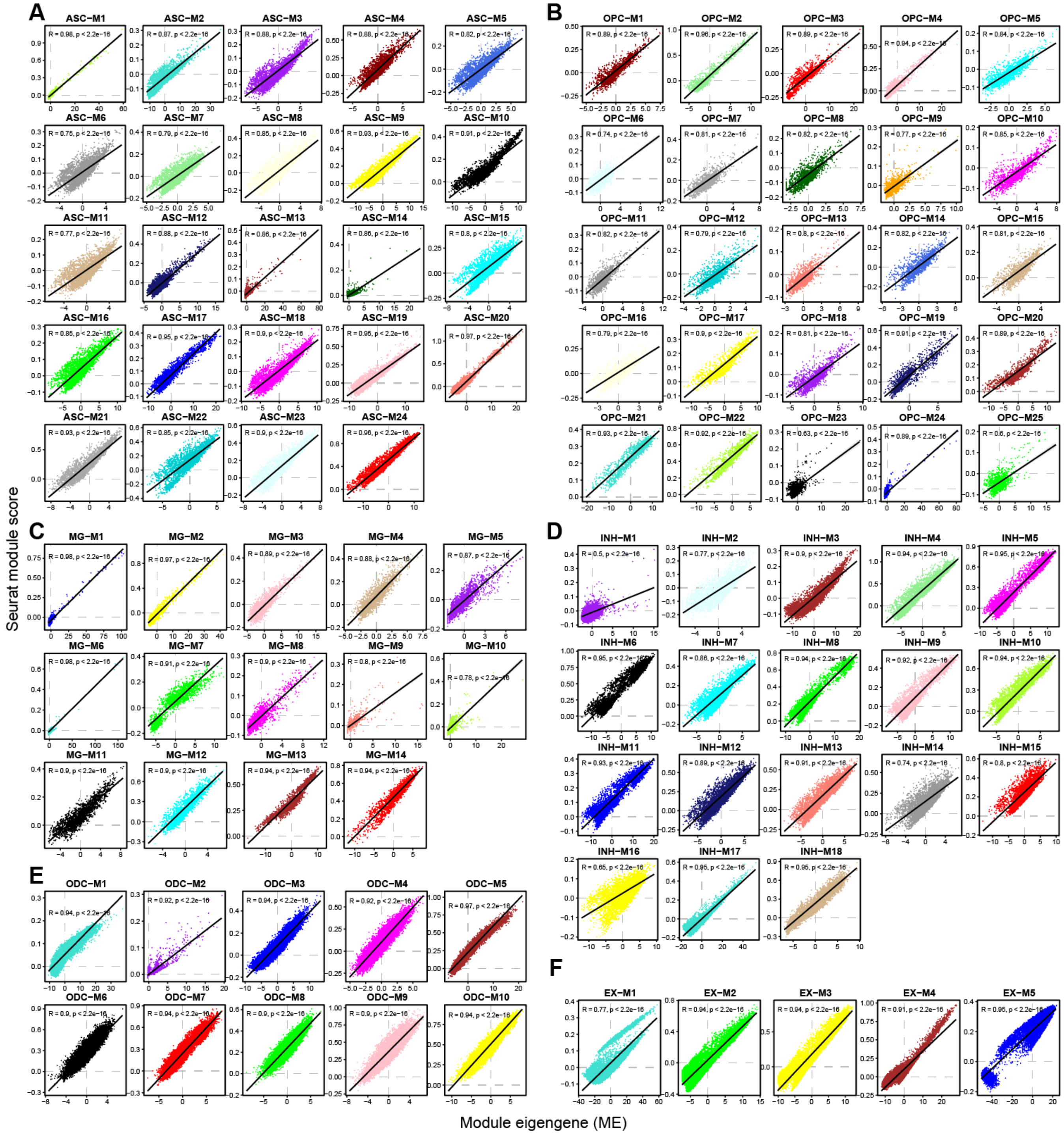

Figure S3. Correlation of module eigengenes and Seurat module scores, related to Figure 1. For each module in the human prefrontal cortex (PFC) snRNA-seq dataset[7], we computed Seurat module scores using the *AddModuleScore* function, and correlated with module eigengenes (MEs). We visualized the results as scatter plots with linear regression lines (95% confidence interval shown in grey) for ASC (**A**), OPC (**B**), MG (**C**), INH (**D**), ODC (**E**), and EX (**F**).

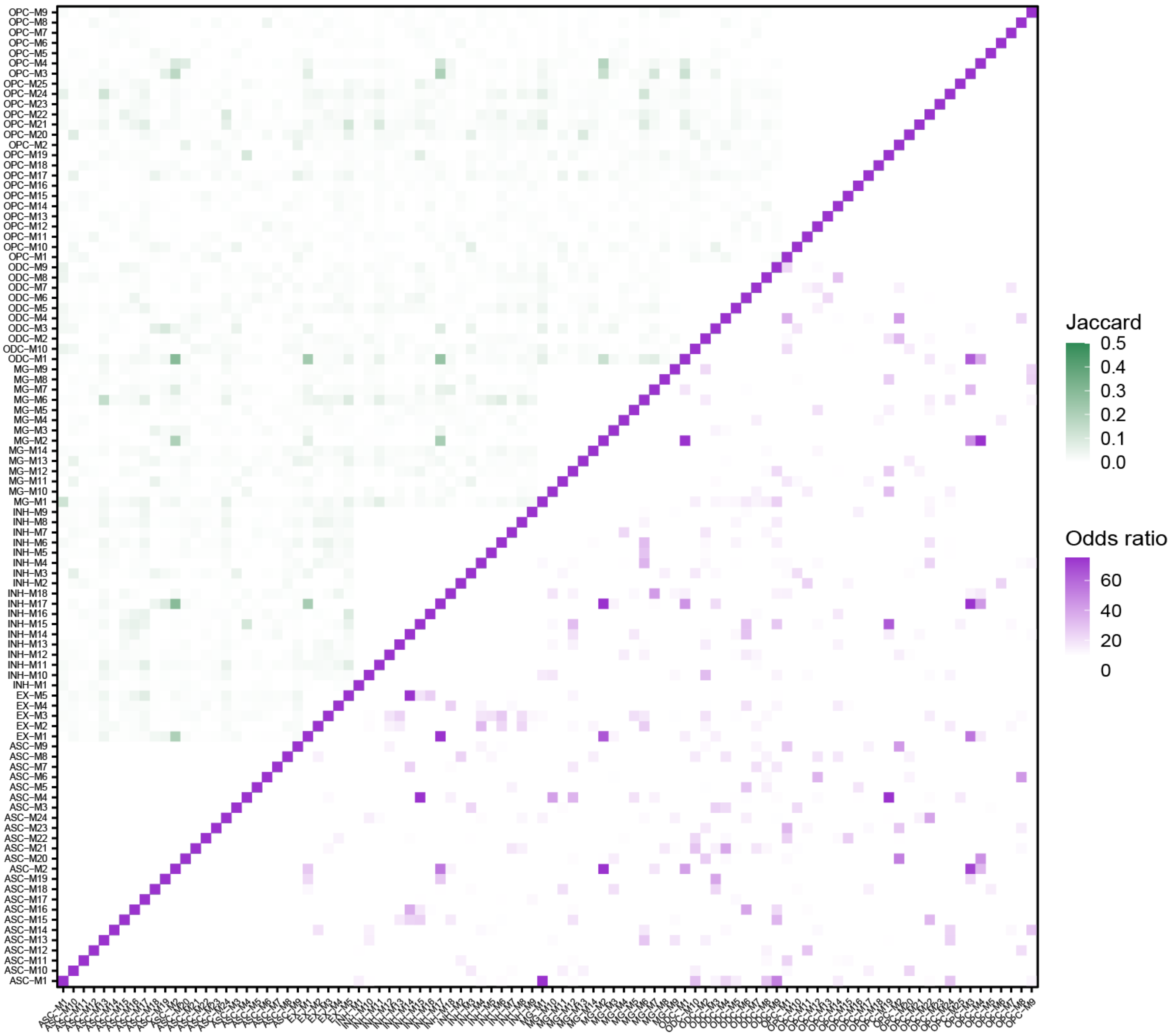

Figure S4. Module overlap analysis of cell-type-specific human PFC co-expression modules, related to Figure 1. We used Fisher's exact test to perform pairwise comparisons of gene sets from the 96 cell-type-specific co-expression modules from the Zhou *et al.* 2019 human prefrontal cortex (PFC) dataset[7]. We report the results of these module overlap tests using the Jaccard index in the upper triangle of the heatmap, and the odds ratio in the lower triangle. The diagonal of the heatmap represents the module overlapping with itself.

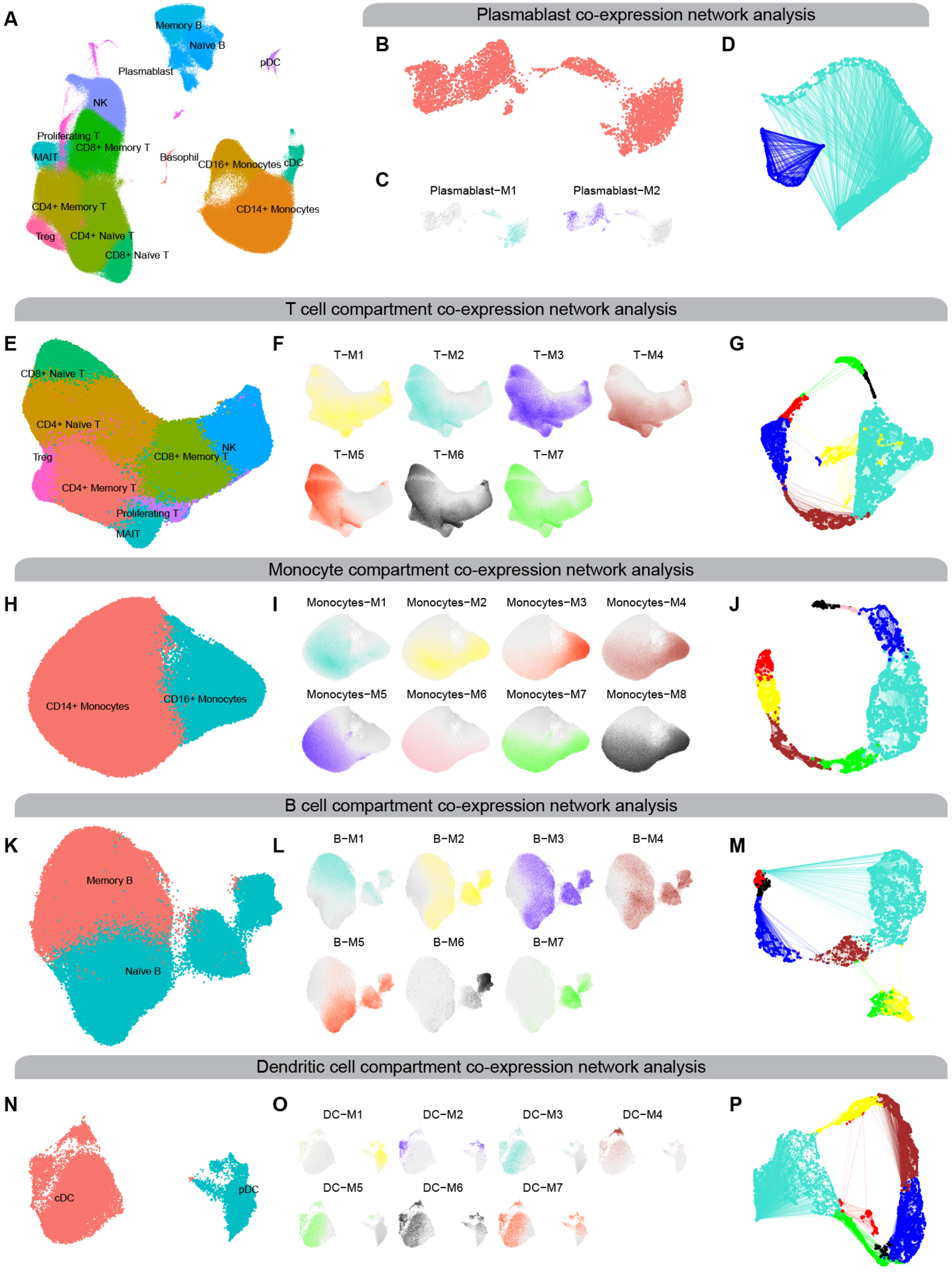

Figure S5. Iterative co-expression network analysis of major cell compartments in the Parse Biosciences 1M PBMC dataset, related to Figure 1. A. UMAP plot of 965,363 PBMCs from 12 healthy donors and 12 Type-1 diabetic donors profiled with the Parse Biosciences Evercode Whole Transcriptome Mega protocol. Cells are colored by cell-type annotations. **B,E,H,K,N.** Individual UMAP plots computed for each cell compartment separately for each cell compartment. **C,F,I,L,O.** UMAP plots for each cell compartment colored by module eigengenes (MEs). **D,G,J,M,P** UMAP plots of the co-expression networks for each cell compartment, colored by gene module assignment. Nodes represent genes and edges represent co-expression links. Network edges were downsampled for visual clarity.

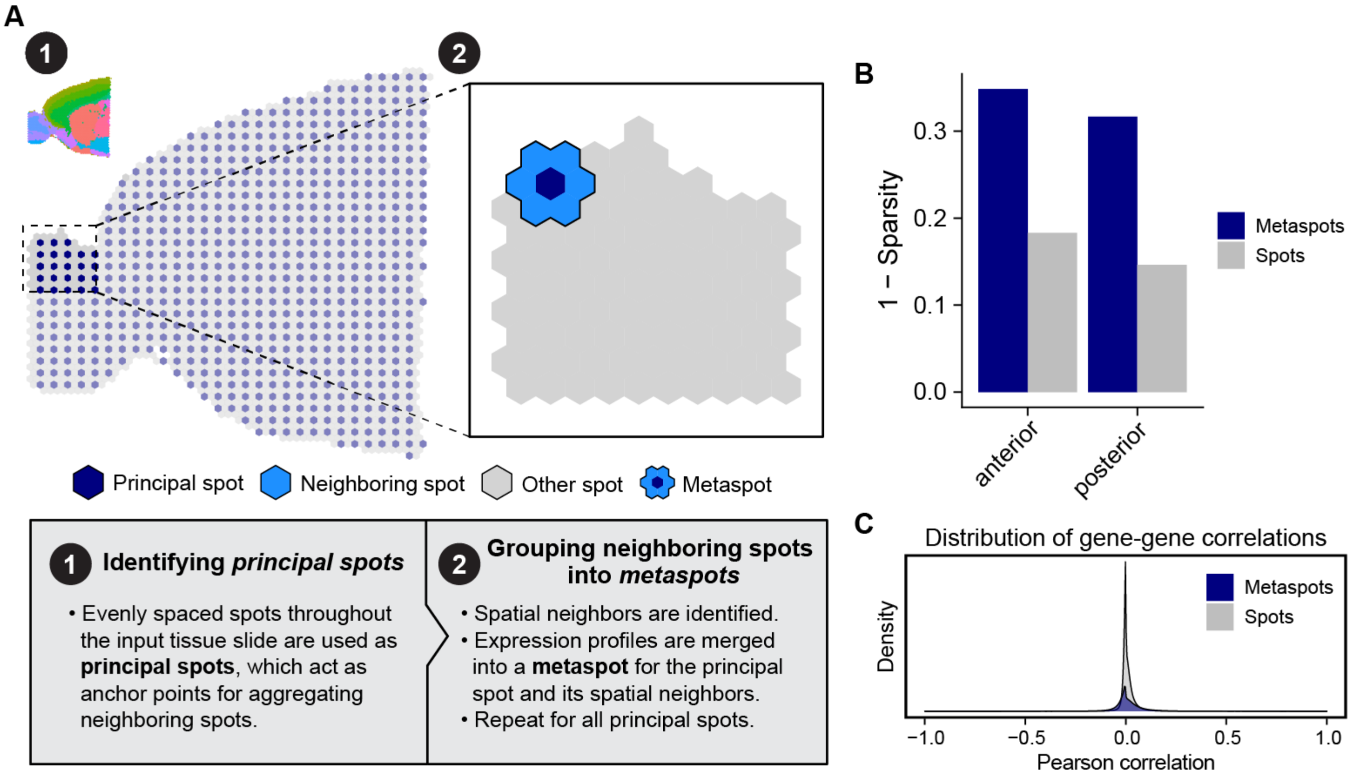

Figure S6. Metaspot aggregation for co-expression network analysis in spatial transcriptomics, related to Figure 3. A. Schematic representation of the metaspot construction process. A grid of evenly spaced *principal spots* are specified throughout the given input ST section. The expression values for each principal spot and its direct neighbors are merged into a single metaspot expression profile. This procedure yields a metaspot expression matrix for the given input ST section. **B.** Expression matrix density (1 - sparsity) for the ST and metaspot expression matrices in the anterior and posterior mouse brain samples. **C.** Density plot showing the distribution of pairwise Pearson correlations between genes from the ST expression matrix and the metaspot expression matrix.

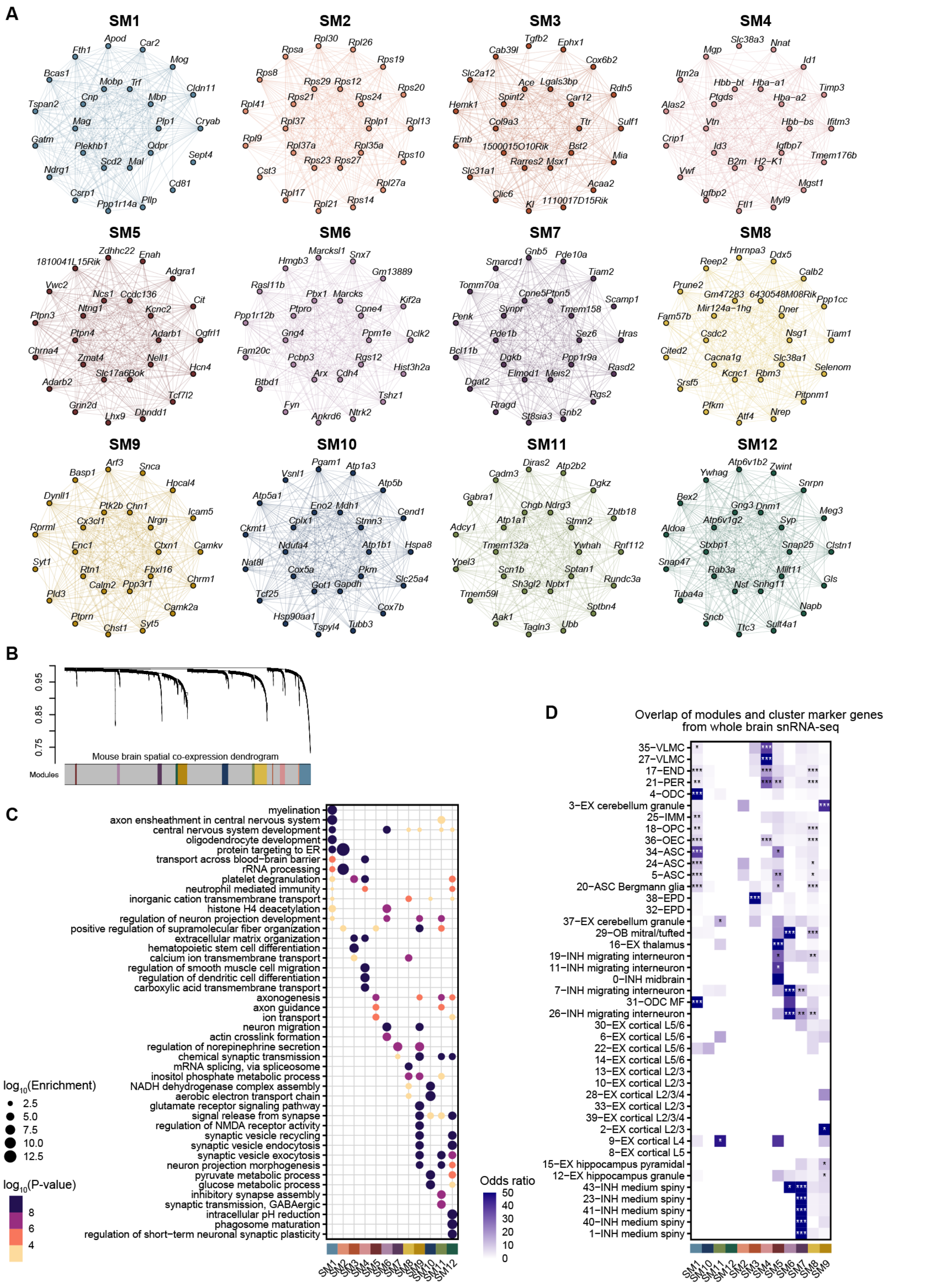

Figure S7. Mouse brain spatial transcriptomics co-expression network analysis, related to Figure 3. Caption on the next page ⟶ **A.** Hub gene networks for each spatial co-expression module. The top 25 hub genes ranked by kME are visualized. Nodes represent genes, and edges represent co-expression links. **B.** Dendrogram showing the hierarchical clustering of genes into co-expression modules based on the topological overlap matrix (TOM). **C.** Dot plot showing selected GO term enrichment results for each co-expression module. **D.** Heatmap showing gene overlap tests (Fisher's exact test) comparing the spatial co-expression modules to differentially expressed genes (DEGs) in each cluster from a whole mouse brain snRNA-seq dataset [8].

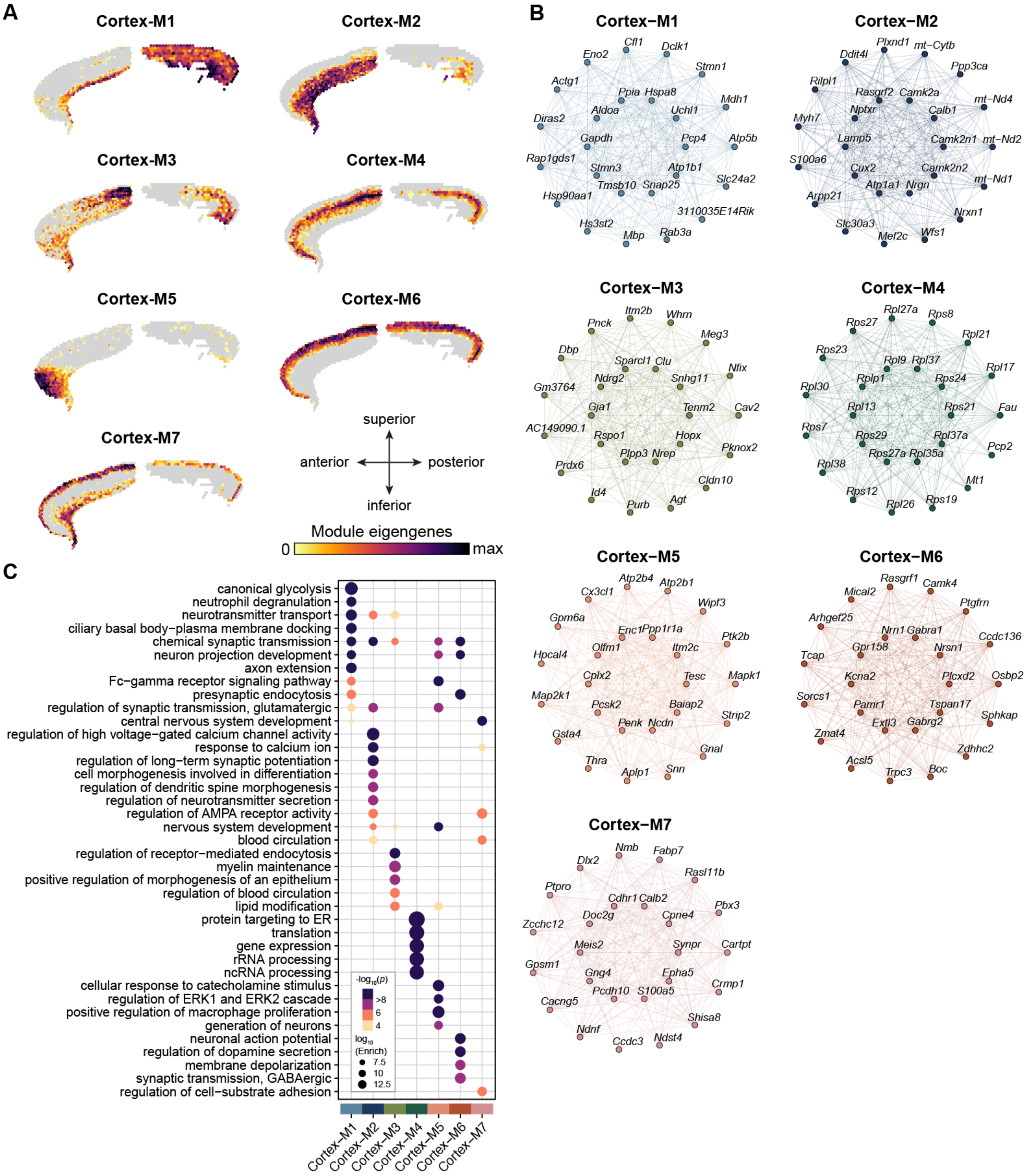

Figure S8. Mouse brain spatial transcriptomics co-expression network in cortical layers 2-6, related to Figure 3. A. ST samples colored by module eigengenes (MEs) for the seven cortical spatial co-expression modules. Grey color indicates a ME values less than zero. **B.** Hub gene networks for each cortical co-expression module. The top 25 hub genes ranked by kME are visualized. Nodes represent genes, and edges represent co-expression links. **C.** Dot plot showing selected GO term enrichment results for each co-expression module.

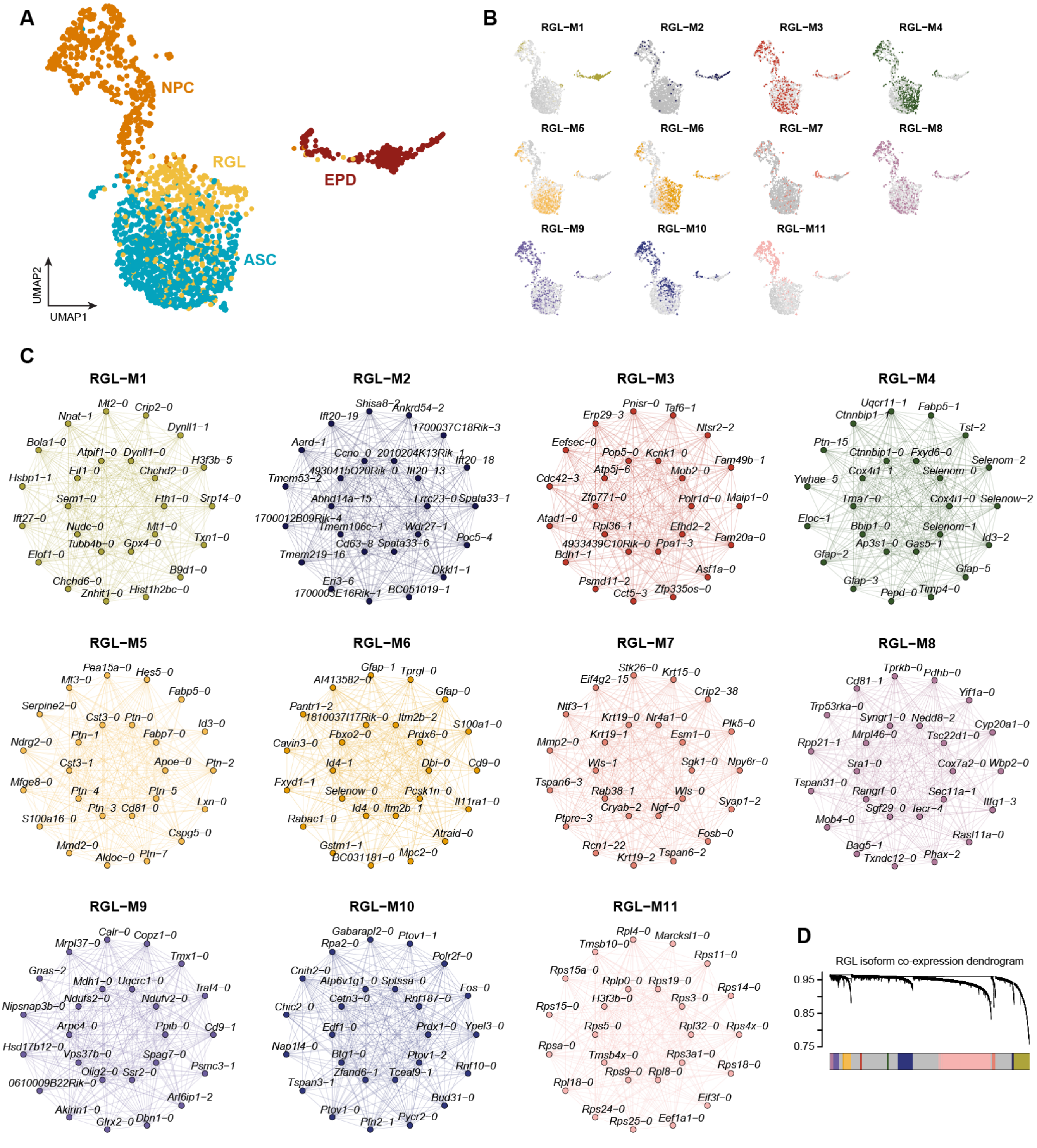

Figure S9. Isoform co-expression network analysis in the mouse hippocampus, related to Figure 4. A. UMAP plot of the radial glia lineage clusters from the mouse hippocampus ScISOrSeq dataset[9]. Cell type abbreviations are the following: ASC: astrocytes; EPD: ependymal cells; NPC: neuronal intermediate progenitor cells; RGL: radial glia. **B.** UMAP plots as in **a.** colored by MEiso for the eleven isoform co-expression modules. **C.** Hub isoform networks for each radial glia lineage co-expression modules. The top 25 hub genes ranked by kMEiso are visualized. Nodes represent isoforms, and edges represent co-expression links. **D.** Dendrogram showing the hierarchical clustering of isoforms into co-expression modules based on the topological overlap matrix (TOM).

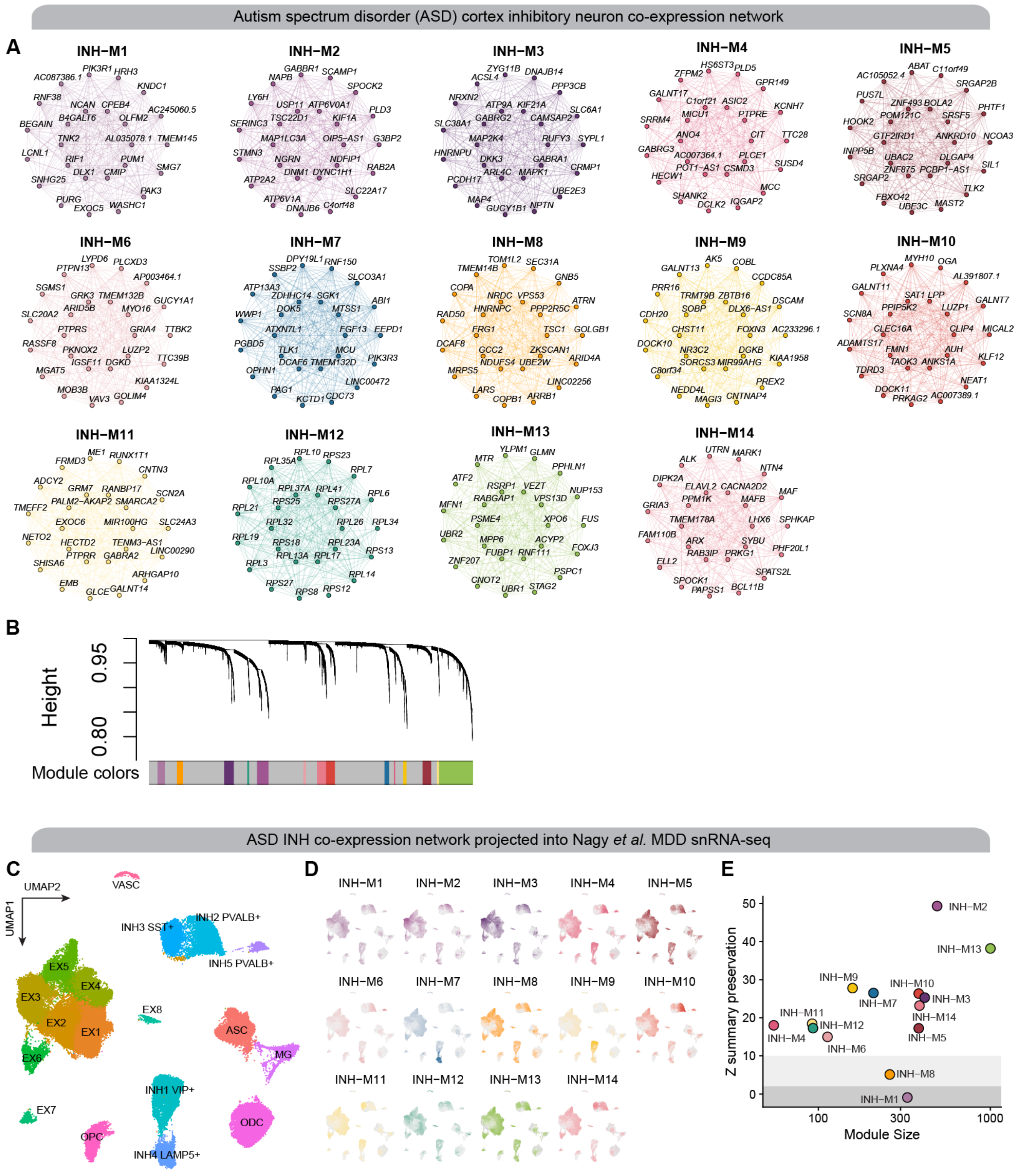

Figure S10. Co-expression network analysis of inhibitory neurons in Autism spectrum disorder, related to Figure 5. A. Hub gene networks for each inhibitory neuron co-expression modules. The top 25 hub genes ranked by kME are visualized. Nodes represent genes, and edges represent co-expression links. **B.** Dendrogram showing the hierarchical clustering of genes into co-expression modules based on the topological overlap matrix (TOM). **C.** UMAP plot of the snRNA-seq dataset of human major depressive disorder (MDD) [10]. Cells are colored by cluster annotations. Cell type abbreviations are the following: ASC: astrocytes; EX: excitatory neurons; INH: inhibitory neurons; MG: microglia; ODC: oligodendrocytes; OPC: oligodendrocyte progenitor cells; VASC: vascular cells. **D.** UMAP plots of the MDD dataset as in **C.** colored by the MEs projected from the ASD dataset. **E.** Module preservation statistics for the ASD inhibitory neuron modules in the inhibitory neuron population from the MDD dataset. Z-summary preservation < 2 indicates no evidence of module preservation, Z-summary preservation < 10 indicates moderate evidence of module preservation, and Z-summary preservation > 10 indicates high evidence of module preservation

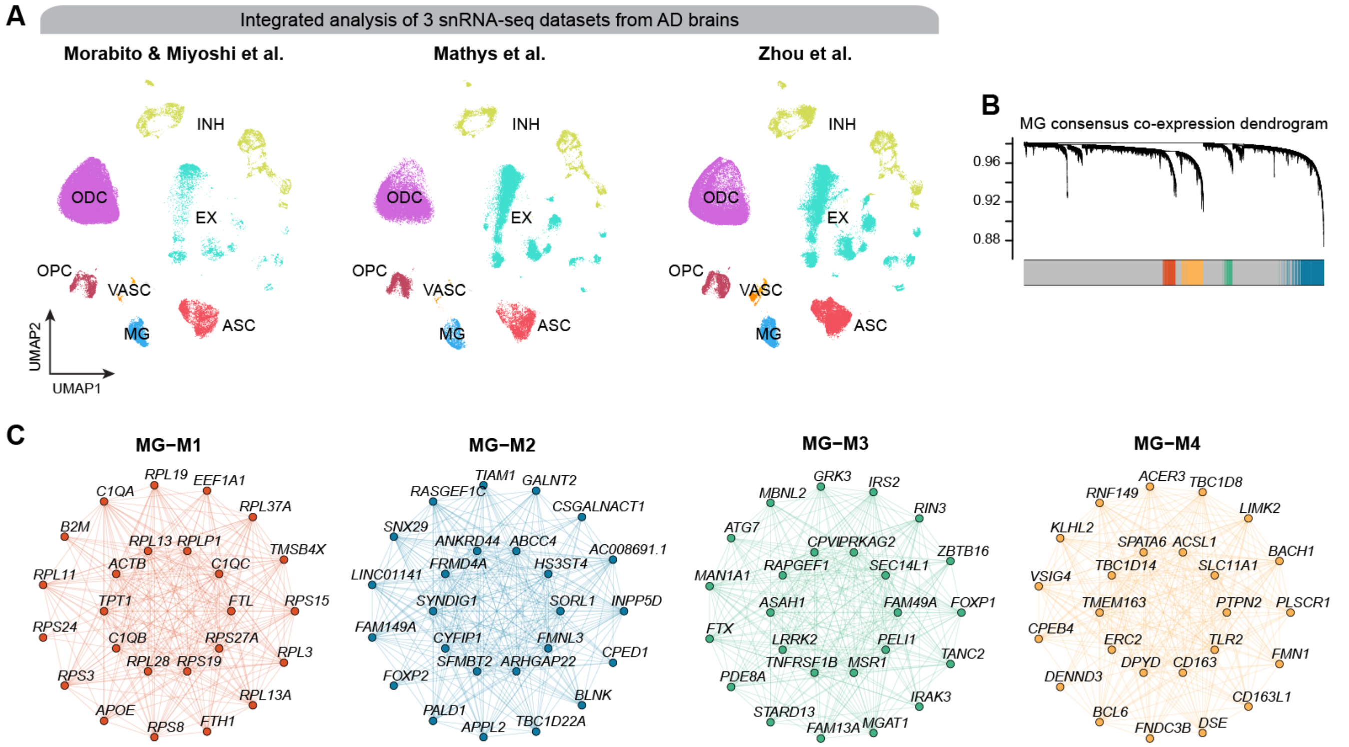

Figure S11. Consensus co-expression network analysis of microglia in Alzheimer's disease, related to Figure 6. A. UMAP plot of the integrated dataset from three AD snRNA-seq studies [7,11,12], colored by cell type assignment. Cell type abbreviations are the following: ASC: astrocytes; EX: excitatory neurons; INH: inhibitory neurons; MG: microglia; ODC: oligodendrocytes; OPC: oligodendrocyte progenitor cells; VASC: vascular cells. **B.** Dendrogram showing the hierarchical clustering of genes into co-expression modules based on the consensus topological overlap matrix (TOM) from the three snRNA-seq datasets. **C.** Hub gene networks for each microglia consensus co-expression modules. The top 25 hub genes ranked by kME are visualized. Nodes represent genes, and edges represent co-expression links.

References

1. Butler, A., Hoffman, P., Smibert, P., Papalexi, E., and Satija, R. (2018). Integrating single-cell transcriptomic data across different conditions, technologies, and species. Nature Biotechnology *36*, 411–420. doi:10.1038/nbt.4096.

2. Stuart, T., Butler, A., Hoffman, P., Hafemeister, C., Papalexi, E., Mauck, W. M., Hao, Y., Stoeckius, M., Smibert, P., and Satija, R. (2019). Comprehensive Integration of Single-Cell Data. Cell *177*, 1888–1902.e21. doi:10.1016/j.cell.2019.05.031.

3. Hao, Y., Hao, S., Andersen-Nissen, E., Mauck, W. M., Zheng, S., Butler, A., Lee, M. J., Wilk, A. J., Darby, C., Zager, M., Hoffman, P., Stoeckius, M., Papalexi, E., Mimitou, E. P., Jain, J., Srivastava, A., Stuart, T., Fleming, L. M., Yeung, B., Rogers, A. J., McElrath, J. M., Blish, C. A., Gottardo, R., Smibert, P., and Satija, R. (2021). Integrated analysis of multimodal single-cell data. Cell *184*, 3573–3587.e29. doi:10.1016/j.cell.2021.04.048.

4. Wolf, F. A., Angerer, P., and Theis, F. J. (2018). SCANPY: large-scale single-cell gene expression data analysis. Genome Biology *19*, 15. doi:10.1186/s13059-017-1382-0.

5. Persad, S., Choo, Z.-N., Dien, C., Sohail, N., Masilionis, I., Chaligné, R., Nawy, T., Brown, C. C., Sharma, R., Pe'er, I., Setty, M., and Pe'er, D. (2023). SEACells infers transcriptional and epigenomic cellular states from single-cell genomics data. Nature Biotechnology (1–12). doi:10.1038/s41587-023-01716-9.

6. Plaisier, S. B., Taschereau, R., Wong, J. A., and Graeber, T. G. (2010). Rank–rank hypergeometric overlap: identification of statistically significant overlap between gene-expression signatures. Nucleic Acids Research *38*, e169–e169. doi:10.1093/nar/gkq636.

7. Zhou, Y., Song, W. M., Andhey, P. S., Swain, A., Levy, T., Miller, K. R., Poliani, P. L., Cominelli, M., Grover, S., Gilfillan, S., Cella, M., Ulland, T. K., Zaitsev, K., Miyashita, A., Ikeuchi, T., Sainouchi, M., Kakita, A., Bennett, D. A., Schneider, J. A., Nichols, M. R., Beausoleil, S. A., Ulrich, J. D., Holtzman, D. M., Artyomov, M. N., and Colonna, M. (2020). Human and mouse single-nucleus transcriptomics reveal TREM2-dependent and TREM2-independent cellular responses in Alzheimer's disease. Nature Medicine *26*, 131–142. doi:10.1038/s41591-019-0695-9.

8. Shabestari, S. K., Morabito, S., Danhash, E. P., McQuade, A., Sanchez, J. R., Miyoshi, E., Chadarevian, J. P., Claes, C., Coburn, M. A., Hasselmann, J., Hidalgo, J., Tran, K. N., Martini, A. C., Rothermich, W. C., Pascual, J., Head, E., Hume, D. A., Pridans, C., Davtyan, H., Swarup, V., and Blurton-Jones, M. (2022). Absence of microglia promotes diverse pathologies and early lethality in Alzheimer's disease mice. Cell Reports *39*, 110961. doi:10.1016/j.celrep.2022.110961.

9. Joglekar, A., Prjibelski, A., Mahfouz, A., Collier, P., Lin, S., Schlusche, A. K., Marrocco, J., Williams, S. R., Haase, B., Hayes, A., Chew, J. G., Weisenfeld, N. I., Wong, M. Y., Stein, A. N., Hardwick, S. A., Hunt, T., Wang, Q., Dieterich, C., Bent, Z., Fedrigo, O., Sloan, S. A., Risso, D., Jarvis, E. D., Flicek, P., Luo, W., Pitt, G. S., Frankish, A., Smit, A. B., Ross, M. E., and Tilgner, H. U. (2021). A spatially resolved brain region- and cell type-specific isoform atlas of the postnatal mouse brain. Nature Communications *12*, 463. doi:10.1038/s41467-020-20343-5.

10. Nagy, C., Maitra, M., Tanti, A., Suderman, M., Théroux, J.-F., Davoli, M. A., Perlman, K., Yerko, V., Wang, Y. C., Tripathy, S. J., Pavlidis, P., Mechawar, N., Ragoussis, J., and Turecki, G. (2020). Single-nucleus transcriptomics of the prefrontal cortex in major depressive disorder implicates oligodendrocyte precursor cells and excitatory neurons. Nature Neuroscience *23*, 771–781. doi:10.1038/s41593-020-0621-y.

11. Morabito, S., Miyoshi, E., Michael, N., Shahin, S., Martini, A. C., Head, E., Silva, J., Leavy, K., Perez-Rosendahl, M., and Swarup, V. (2021). Single-nucleus chromatin accessibility and transcriptomic characterization of Alzheimer's disease. Nature Genetics *53*, 1143–1155. doi:10.1038/s41588-021-00894-z.

12. Mathys, H., Davila-Velderrain, J., Peng, Z., Gao, F., Mohammadi, S., Young, J. Z., Menon, M., He, L., Abdurrob, F., Jiang, X., Martorell, A. J., Ransohoff, R. M., Hafler, B. P., Bennett, D. A., Kellis, M., and Tsai, L.-H. (2019). Single-cell transcriptomic analysis of Alzheimer's disease. Nature *570*, 332–337. doi:10.1038/s41586-019-1195-2.
